# Precious Metal Free Hydrogen Evolution Catalyst Design and Application

**DOI:** 10.1021/acs.chemrev.3c00712

**Published:** 2024-04-25

**Authors:** Anders A. Feidenhans’l, Yagya N. Regmi, Chao Wei, Dong Xia, Jakob Kibsgaard, Laurie A. King

**Affiliations:** † Department of Physics, Technical University of Denmark, 2800 Kongens Lyngby, Denmark; ‡ Faculty of Science and Engineering, Manchester Metropolitan University, Manchester M1 5GD, U.K.; § Manchester Fuel Cell Innovation Centre, Manchester Metropolitan University, Manchester M1 5GD, U.K.

## Abstract

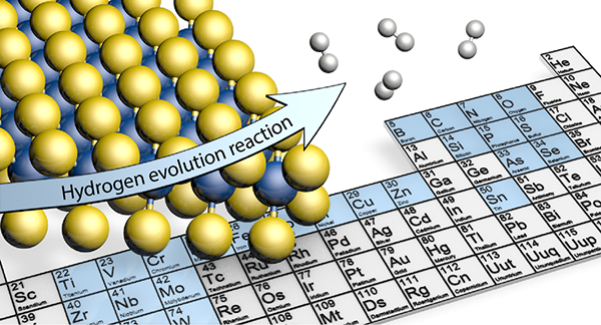

The quest to identify precious metal free hydrogen evolution reaction catalysts has received unprecedented attention in the past decade. In this Review, we focus our attention to recent developments in precious metal free hydrogen evolution reactions in acidic and alkaline electrolyte owing to their relevance to commercial and near-commercial low-temperature electrolyzers. We provide a detailed review and critical analysis of catalyst activity and stability performance measurements and metrics commonly deployed in the literature, as well as review best practices for experimental measurements (both in half-cell three-electrode configurations and in two-electrode device testing). In particular, we discuss the transition from laboratory-scale hydrogen evolution reaction (HER) catalyst measurements to those in single cells, which is a critical aspect crucial for scaling up from laboratory to industrial settings but often overlooked. Furthermore, we review the numerous catalyst design strategies deployed across the precious metal free HER literature. Subsequently, we showcase some of the most commonly investigated families of precious metal free HER catalysts; molybdenum disulfide-based, transition metal phosphides, and transition metal carbides for acidic electrolyte; nickel molybdenum and transition metal phosphides for alkaline. This includes a comprehensive analysis comparing the HER activity between several families of materials highlighting the recent stagnation with regards to enhancing the intrinsic activity of precious metal free hydrogen evolution reaction catalysts. Finally, we summarize future directions and provide recommendations for the field in this area of electrocatalysis.

## 1 Introduction

As we shift to a Net Zero emission landscape, hydrogen continues to gain significant traction as an enabling technology for this transition.^1−3^ Indeed globally, numerous policies, ambitions and projects have been announced relating to hydrogen. The continuing growth of this ambition is clearly evidenced in annual updates in recent IEA reports where the cumulative deployment for low-emission hydrogen production was 74 GW by 2030 in 2021, whereas in 2022 it is reported that this ambition has grown to 145–190 GW by 2030.^4^ It is currently estimated that this growth will reach 16–24 Mt per year of low-emission hydrogen, with 9–14 Mt from electrolysis and 7–10 Mt from fossil fuel technologies coupled to carbon capture utilization and storage. To meet this demand, 134–240 GW installed electrolyzer capacity is forecast for installation by 2030, requiring huge expansion of electrolyzer manufacturing capacity.

While many components of an electrolyzer plays critical roles that dictates the performance (e.g., efficiency, longevity, cost, etc.), it is at the catalyst surfaces where the electrochemical reactions proceed. In current commercial water electrolyzers, the relevant electrochemical reactions are the cathodic hydrogen evolution reaction (HER) and the anodic oxygen evolution reaction (OER). Predominantly, precious metal (platinum, iridium and ruthenium) catalysts are utilized to lower the overpotentials of HER and OER in contemporary proton exchange membrane (PEM-WE) commercial water electrolyzers.^5,6^ Conversely, commercial alkaline water electrolyzers (A-WE) typically deploy precious metal free catalysts (nickel, iron). Due to the scarcity and costs of precious metals, there have been a tremendous number of reports on the efficacy of precious metal free catalysts for water electrolysis.

In this review we provide a comprehensive review of precious metal free (sometimes called nonprecious metal, or nonplatinum group) catalyst development for the HER under both acidic and alkaline electrolyte for low-temperature electrolysis. The review is split into three sections. In the first section, we describe current commercial electrolyzers to give the reader context for the environment in which electrocatalysts must operate. Specifically, we provide a succinct overview of A-WE, PEM-WE and anion exchange membrane electrolyzer (AEM-WE) technologies. Subsequently, we provide an overview of the fundamentals of the HER, including an introduction to the mechanisms of the HER. In the second section of this review, we describe benchmarking and best practices which have been widely discussed for assessing HER catalysts in the literature. This section reviews the various reviews, publications, perspectives and editorials that provide best practices for screening HER catalysts in three electrode (half-cell) measurements. Additionally, we discuss the challenges and limitations of translating performance and durability from half-cells to devices, including fabricating membrane electrode assemblies, providing a review of testing methods as well as examples of precious metal free catalysts that have been translated into such device testing. In the third section of the review, we provide an overview of the various design strategies for precious metal free HER metal catalysts, highlighting the key motivations for each (e.g., higher density of active sites, higher surface area, increased conductivity, enhanced stability). Subsequently, we showcase some of the most popular catalyst families deployed for the HER in acidic (molybdenum disulfide-based, transition metal phosphides and transition metal carbides) as well as alkaline (nickel molybdenum and transition metal phosphides) catalysts. For each, we provide a comprehensive review of the key approaches and advances developed. Owing to the vast quantity of publications, the review largely focuses on paper published within the last five years that are highly cited, or hot papers (as denoted by Web of Science). However, numerous additional historical references are also cited to provide a more holistic and comprehensive background to the development of precious metal free HER catalysts.

### 1.1 Commercial and near-Commercial Electrolyzers: HER Catalysts in Context

Two technologies dominate the low-temperature electrolyzer market today: A-WE and PEM-WE.^7,8^ Installed global capacity is currently estimated at 60% A-WE and 34% PEM-WE (the balance either unknown or alternative technologies such as solid oxide electrolysis).^9^ Typical operating conditions and components are provided in Table 1, highlighting some of the stark differences between these technologies. It is widely acknowledged that in addition to A-WE and PEM-WE, AEM-WE will also contribute to the portfolio of low-temperature hydrogen production technologies in the coming decade(s).^10−12^ Beyond low-temperature hydrogen production technologies, solid oxide water electrolyzers (SO-WE) technologies which operate at elevated temperatures (∼800 °C) are also anticipated to play a role in the future of green hydrogen production.^13−16^

There exist many differences between A-WE, PEM-WE and AEM-WE technologies including the chemical environment, components and device designs. As highlighted by their names, obvious distinctions among A-WE, PEM-WE and AEM-WE are the electrolytes deployed. Critically, A-WE deploy a liquid electrolyte. Conversely, PEM-WE and AEM-WE both deploy a solid polymer electrolyte. The solid electrolyte is a proton exchange membrane for PEM-WE, and an anion exchange membrane for AEM-WE. As a consequence of the electrolyte, the electrocatalysts (and other cell components) are required to withstand either a highly acidic (PEM-WE) or alkaline (A-WE and AEM-WE) pH. Accordingly, screening of potential HER catalyst for A-WE and AEM-WE technologies must ensure they are active and stable under highly alkaline conditions, and conversely, PEM-WE electrocatalysts must be active and stable under highly acidic condition.

**Figure 1:**
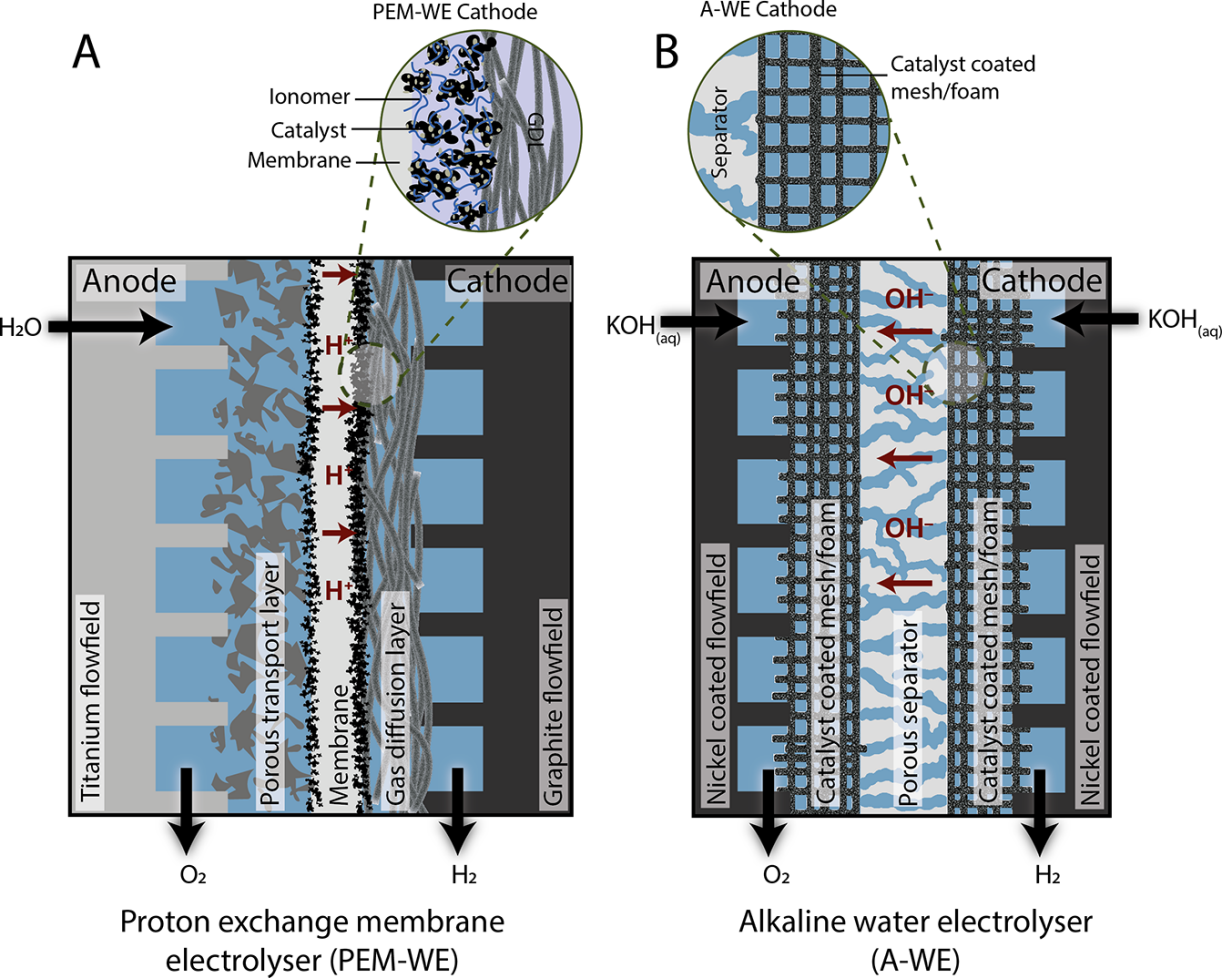
Schematic of zero-gap cell architecture and cathode zoom in to show the HER catalyst environment for typical (A) PEM-WE and (B) A-WE.

Beyond differences in the pH, A-WE, PEM-WE and AEM-WE can each have very different cell architectures. For example, the cell architecture can be a “gap” or “zero-gap” cell. This terminology relates to whether a physical space exists between the electrodes and separator/membrane. Specifically, “zero-gap” refers to the cell architecture whereby the membrane/diaphragm is directly sandwiched between the two electrodes (e.g., Figure 1). Conversely, a “gap” cell is where there exists a space between the membrane/diaphragm and the electrodes. Industrial A-WE were previously “gap” cells, however the majority of commercial A-WE are now “zero-gap”. PEM-WE and AEM-WE designs are also “zero-gap”. Typical cell configurations for A-WE and PEM-WE are shown in Figure 1.

A-WE utilize approximately 20–40 wt % KOH as a conductive electrolyte, operating at approximately 50–90 °C (Table 1). The A-WE uses a separator (or permeable diaphragm) between the anode and cathode to prevent the mixing of O_2_ and H_2_ within the electrolyzer while enabling conduction of hydroxide anions. Commercial A-WE are today zero-gap cells, typically utilizing a porous diapraghm of ZrO_2_ particles on polyphenylene sulfide. A benefit of this design is the rapid degassing of the evolved hydrogen and oxygen, which otherwise would form a supersaturated solution in the electrolyte. As the bubbles adhere to the electrode, the electrolyte saturation is kept relatively low compared to PEM-WE, which minimizes the crossover of gases across the diaphragm.^18^ Detailed reviews focused on A-WE technology are published elsewhere.^19−21^ Owing to the alkaline environment in an A-WE, stainless steel and precious metal free nickel components are deployed. At the cathode, Raney Ni and Ni alloy electrodes are commonly deployed as high-surface area electrodes for the HER. The cathode and anode catalysts are typically prepared directly onto a mesh or foam structure (typically nickel or nickel coated). Commercial A-WEs have been deployed at industrial scale for over 90 years, with significant advances in the 1920s and 1930s leading to industrial sites with production at 10,000 N m^3^ h^–1^.^6^

Membrane-based water electrolyzers (PEM-WE and AEM-WE) utilize an electrically insulating solid membrane as an electrolyte which is sandwiched between the anode and cathode. Collectively, the anode, cathode and membrane make up a membrane electrode assembly (MEA). When coupled to thin polymer electrolytes with relatively low internal resistances, this zero-gap cell architecture enables low gas crossover. Utilizing an acidic electrolyte membrane, PEM-WEs typically operate between 50–80 °C. PEM-WE have been reviewed in detail elsewhere.^6,22−24^ PEM-WEs are fed a pure water supply for water splitting. Owing to the PEM-WE membrane chemistry (Nafion, fumapem, perfluorosulfonate polymers), protons are selectively transported between the anode and cathode and hence the catalysts deployed in a PEM-WE are exposed to a highly acidic environment. This is owing to the polyfluorosulfonic acid (PFSA) polymer materials (e.g., Nafion) used as membranes, reviewed elsewhere.^25^ Nafion is a copolymer, with an electrically neutral polytetrafluoroethylene polymeric backbone polymers, with side-chains that end with sulfonic acid (−SO_3_H) functional groups that loosely bind protons. The side chains are randomly distributed along the fluorinated backbone and facilitate proton transfer across the membrane. Such PFSA materials are commonly deployed as membranes as well as ionomers deployed within the catalyst layer (Figure 1). In commercial PEM-WEs, platinum catalysts on carbon supports are deployed to turn over the cathodic HER. At the anode, iridium and ruthenium oxide-based catalysts are deployed. PEM-based electrolyzers were first developed at General Electric in 1955, and later used for oxygen generation in space and submarine applications.^6^

**Table 1:** Typical Operating Conditions and Device Components of Alkaline Water Electrolyzers, Proton Exchange Membrane Electrolyzer, and Anion Exchange Membrane Electrolyzers^7,8,17^

	Alkaline (A-WE)	Proton exchange membrane (PEM-WE)	Anion exchange membrane (AEM-WE)
**Electrolyte**	Aqueous KOH (20–40 wt %)	Proton exchange membranes	Anion exchange membranes
**Cathode catalysts**	Ni, Ni based alloys, Ni-coated stainless steel, Ni_*x*_P_*y*_, Ni_*x*_S_*y*_, Ru	Pt–C, Pt-black	Ni and Ni alloys, Pt-alloys, Ru-alloys
**Anode catalysts**	NiFeOOH, LaNiO_3_, Ni–Co alloy, Co_3_O_4_	IrO_*x*_, IrRu-oxides	Ni, Ni alloys, Ni-based oxides, IrO_2_
**Operating temperature (°C)**	50–90	50–80	40–60
**Operating pressure (bar)**	1–35	<70	<35
**Water source**	Deionized water (into aqueous 20–40 wt % KOH)	Deionized water	Water or alkaline water (0.1–1 M KOH)
**Current density**(A cm^–2^)**at 2.0 V**	0.2–1.2	>2	<2

Currently considered precommercial, zero-gap AEM-WEs are far less mature compared to A-WE and PEM-WE technologies. However, PEM-WE and AEM-WEs deploy an MEA cell architecture whereby a membrane separates the anode and cathode.^26^ AEM-WE have been reviewed in detail elsewhere.^11,12,27^ AEM-WE membranes conduct hydroxide ions, utilizing polymers that contain immobilized, positively charged functional groups on their backbone or pendant size chains. It is typically the durability and relatively low conductivity of AEM-WE membranes that is thought to limit the realization of this technology at scale.^27,28^

For AEM-WE, a clear advantage pertaining to the alkaline environment is the suitability of precious metal free catalysts, as well as the replacement of expensive titanium bipolar plates/flow fields. At the cathode, Ni and Ni alloy-based HER electrocatalysts (e.g., NiMo^29^) are considered the highest performing precious metal free catalysts. At the anode, various Ni, Fe and Co oxides are commonly utilized. It is important to note that Pt-based electrocatalysts still outperform precious metal free HER electrocatalysts under alkaline conditions.^30^ One important distinction between current PEM-WEs and AEM-WE literature is that AEM systems are frequently tested with liquid electrolyte circulation (e.g., KOH, or K_2_CO_3_) in addition to the polymer membrane electrolyte (rather than pure water as used in PEM-WE). The role that such electrolyte circulation vs pure water has on AEM-WE durability has recently been reviewed elsewhere.^27^ In brief, it is speculated that the circulation of electrolyte solutions significantly enhances catalyst utilization and improves ohmic resistance owing to the higher hydroxide ion transport, consequently improving intrinsic kinetics of the catalyst compared to a pure water feed.

While precious metal free catalysts are commonly touted as a significant advantage of AEM-WEs, a significant portion of publications (particularly those reporting durability >100 h) still utilize PGM catalysts at the cathode, anode, or both electrodes.^11,31^ It is important to acknowledge that this is likely due to a combination of factors, including; convenience, (commercially available PGM catalysts, inks and manufacturing methods already optimized for PEM-WE) lack of precious metal free benchmarks for AEM components, and the higher intrinsic activity of HER PGM-catalysts under alkaline conditions which therefore reduces the overall cell potential and water splitting efficiency. Furthermore, the complex interplay among the numerous components of an electrolyzer collectively influences the electrolyzer performance. Therefore, it can be extremely challenging to optimize each individual components in isolation without deploying precious metal containing components.

Beyond A-WE, PEM-WE and AEM-WE, it is important to note that the technologies of today may be replaced with alternative cell architectures and device designs beyond these three technologies. For example, capillary-fed electrolyzer cell,^32^ membrane-free electrolyzers,^33−36^ involving soluble (redox) mediators,^37^ photoelectrochemical water splitting^14,15^ which have been more broadly reviewed elsewhere.^38,39^ However, in this review, we focus on catalysts tested under conditions that align with A-WE, AEM-WE and PEM-WE technologies, therefore focusing on catalyst screened for the HER under either alkaline or acidic electrolyte environments.

### 1.2 Water Splitting Fundamentals

Electrochemical water splitting utilizes electricity to split water (H_2_O) into hydrogen (H_2_) and oxygen (O_2_) (eq 1). The choice of electricity source (i.e., renewable, or not) and device efficiency therefore control the emissions associated with the hydrogen produced.
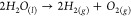
1

Water splitting is a nonspontaneous reaction with a standard Gibbs free energy of 237.2 kJ mol^–1^ which equates to an applied potential of 1.229 V. While the electrical work required to drive eq 1 is critical to understanding the efficiency of an electrolyzer, the reaction is endothermic, therefore requiring an additional energy contribution (48.6 kJ mol^–1^) as heat. This minimum requirement to split water translates to a total energy input of 285.8 kJ mol^–1^ for a water electrolyzer, corresponding to a cell voltage of 1.481 V.^40^

As with any electrochemical device, a water splitting electrolyzer is based on two half-cell reactions: an oxidative and a reductive reaction. The two half-cell reactions of water splitting are the cathodic HER (eqs 2 and 3) and the anodic OER (eqs 4 and 5).

**Hydrogen evolution reaction (HER)**

Acidic medium:
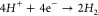
2

Basic medium:
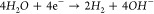
3

**Oxygen evolution reaction (OER)**

Acidic medium:
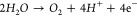
4

Basic medium:
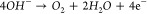
5

The HER is one of the simplest electrochemical reactions, requiring just two electrons to drive the reduction reaction. Indeed, owing to this relatively simple nature, the hydrogen evolution and oxidation, reactions have been used as a platform for the verification of fundamental mechanistic relationships and theories such as the Butler–Volmer equation,^41^ and Sabatier’s principle.^42^

### 1.3 The HER Mechanisms

Under acidic conditions, the HER is generally thought to occur by three different mechanistic steps: the reduction of a proton resulting in an adsorbed hydrogen atom (Volmer reaction), followed by one of two possible pathways, either; (a) molecular hydrogen is formed in a chemical step by the combination of two adsorbed hydrogen atoms, (the Tafel reaction), or (b) a second proton is reduced, which reacts with an adsorbed hydrogen atom to form molecular hydrogen (the Heyrovsky reaction). In both proposed mechanisms, molecular H_2_ is subsequently desorbed from the surface. Note, * denotes an active site on the catalysts.

Volmer reaction
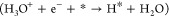
6

Tafel reaction
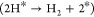
7

Heyrovsky reaction
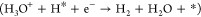
8

Under alkaline conditions, the concentration of hydronium ions is significantly depleted compared to acidic media. Accordingly, for the HER to proceed under alkaline conditions, the catalyst must further facilitate water dissociation in addition to catalyzing the HER. Therefore, contrary to acidic media (where the HER mechanism sources protons from a weakly bound H_3_O^+^), alkaline HER sources protons from water molecules, and thus the Volmer and Heyrovsky steps are rewritten as below:

Volmer reaction
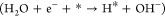
9

Heyrovsky reaction
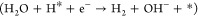
10

Independent of pH, the Tafel step remains constant (eq 7).

Several reviews have been published focused on the HER mechanisms.^43−45^ Several other reviews specifically focus on alkaline HER and contain dedicated sections detailing the alkaline HER mechanism.^46,47^

Experimental reaction rates in alkaline media are typically reported as 1 to 3 orders of magnitude lower than in acidic media for the HER.^48,49^ Water dissociation is generally acknowledged as a contributor to this reduction in activity, and thus the barrier to dissociate water is considered a necessary second descriptor for HER activity in alkaline media.^50^ This was evidenced experimentally by Danilovic et al., in 2013.^51^ Specifically, they measured the HER activity of a selection of metal electrodes, and constructed volcano plots in both acid and base electrolyte. As a function of the selected metal, their position on the volcano varied depending of the pH. However, after mixing the various metals with Ni(OH) nanoparticles as efficient adjacent water dissociation sites, their relative positions in base shifted. Conversely, in acidic media, the relative positions remained constant. Thus, the overall trends in activity as a function of composition were the same for both the base and acidic volcano plots.

**Figure 2:**
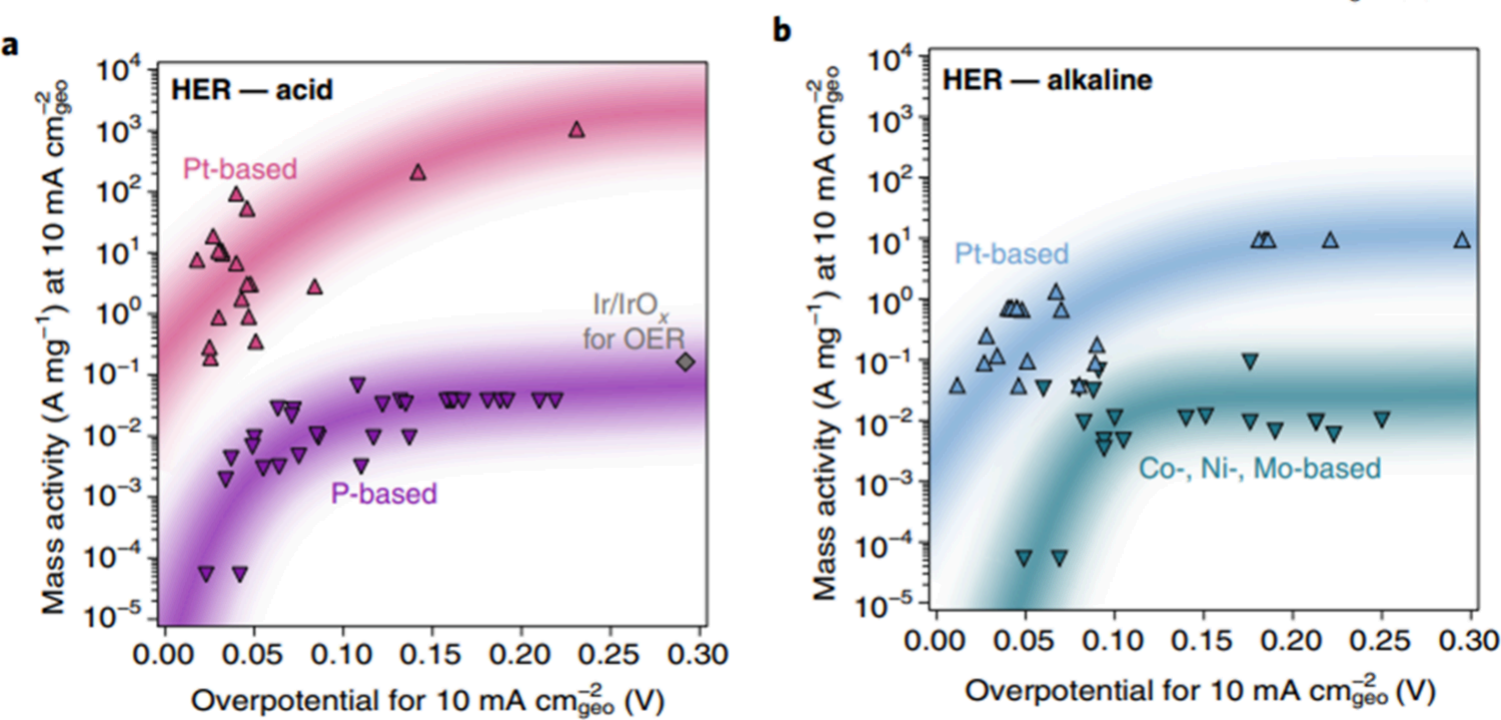
Comparison plots between mass activity (10 mA cm^–2^_geo_) and overpotential (10 mA cm^–2^_geo_) under (A) acidic and (B) alkaline electrolytes for various Pt-based and precious metal free catalysts for the HER. Reproduced with permissions from ref (49). Copyright 2019 Springer Nature.

Despite a plethora of precious metal free catalyst discoveries for the HER, the intrinsic activity of platinum for the HER exceeds that of any alternative precious metal free catalysts by at least 3 orders of magnitude (see Section 3.3.4. for further discussions).^52^ Under acidic conditions, the HER kinetics of a platinum cathode are particularly fast.^52^ Indeed, the intrinsic activity of Pt is so fast, that even when measured in a rotating disk electrode configuration, mass-transport limitations can impede the accurate determination of the intrinsic activity of Pt.^52,53^ Under alkaline conditions, platinum electrodes also outperform precious metal free based electrodes. However, owing to unfavorable kinetics under alkaline conditions, the activity of Pt-based catalysts in acidic media is significantly higher than those recorded in alkaline solution (Figure 2).^49,52,54−58^ Interestingly, when the highest performing precious metal free HER catalysts in acid and alkaline electrolyte are compared by mass activity, these precious metal free catalysts all have similar mass activities (10^–2^–10^–1^ A mg^–1^ at 10 mA cm^–2^_geo_).

**Figure 3:**
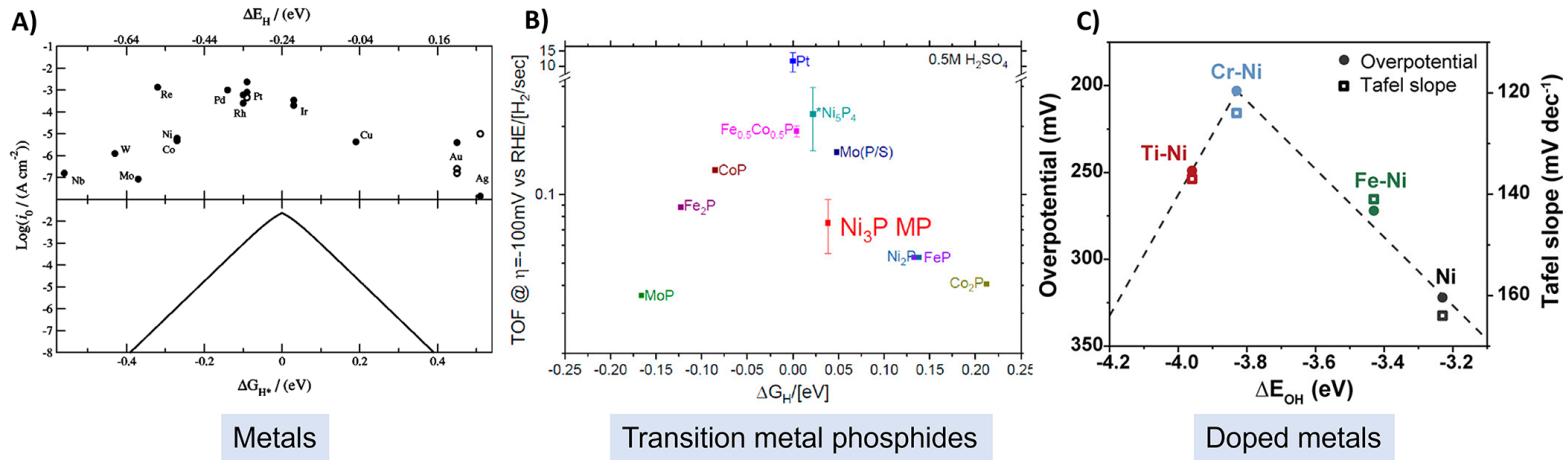
Example volcano plots for various HER catalysts whereby experimental data is plotted against calculated binding energies. (A) Various metallic electrodes (exchange currents plotted against calculated hydrogen adsorption energies) (acidic electrolyte), (B) Numerous transition metal phosphides (experimentally determined TOF at 100 mV overpotential plotted against hydrogen adsorption energies) (acidic electrolyte), (C) Various M-doped Ni electrode surfaces (M = Fe, Cr, Ti) (overpotentials at 10 mA cm^–2^ and Tafel slopes plotted against theoretically calculated OH binding energies) (alkaline electrolyte). Reproduced with permissions from ref (61). Copyright 2005 The Electrochemical Society; ref (63). Copyright 2018 American Chemical Society; ref (77). Copyright 2021 American Chemical Society.

Sabatier’s principle states that there exists a maximum overall rate of reaction when the interaction between reactants and catalysts (binding energy) is neither too strong nor too weak. It was thus proposed for the HER that the overall rate of reaction will largely depend on the free energy of hydrogen adsorption (Δ*G*_H_).^59−61^ Accordingly, where a catalyst Δ*G*_H_ is “too strong”, the desorption of reaction intermediates becomes limiting, blocking catalytically active sites and thus preventing the reaction from proceeding to completion (i.e., molecular hydrogen for the HER). Conversely, where the Δ*G*_H_ is “too weak”, the adsorption of hydrogen atoms will limit the reaction rate. The optimal hydrogen adsorption energy for HER catalysts has typically been considered where Δ*G*_H_ = 0. This relationship leads to a volcano-shaped plot when the catalyst activity is plotted against density function theory (DFT) calculated Δ*G*_H_.^61^ Such trends have been reported for metallic electrodes (Figure 3a),^61,62^ and more recently shown to translate to a wide range of precious metal free HER catalysts including transition metal phosphides (Figure 3b),^63,64^ transition metal carbides,^65−67^ and transition metal nitrides,^68^ deploying a combined theory and experimental approach throughout. Beyond hydrogen binding energies, under alkaline conditions a volcano relationship has been demonstrated for experimentally determined HER activities (overpotentials and Tafel slopes) as a function of OH binding energies (Figure 3c).^69^ These proposed DFT models provide a leading framework for the design of heterogeneous electrocatalysts, and are widely accepted and deployed throughout electrocatalysis materials discovery investigations. Alternative predictors have also been identified as descriptors for HER electrocatalytic activity, including d-band position in relation to the Fermi level,^70^ and surface energies.^71^

It is of note that these volcano-plots are solely based on a thermodynamic assessment, mitigating the kinetically determined barrier energetics.^58,72−74^ Furthermore, while the hydrogen binding energy activity descriptor has been widely demonstrated as an accessible and widely applicable theory broadly for electrocatalysts, it is important to recognize that as a function of pH and electrolyte composition, the correlation of activity with hydrogen binding energies are inconsistent in some reports. Thus, Δ*G*_H_ = 0 can be considered a necessary but insufficient condition for predicting good HER catalysts. Sometimes referred to as “beyond adsorption” descriptors, kinetic parameters (e.g., solvent reorganization energetics) and interfacial electric field strengths, have been highlighted as significant parameters for assessing electrocatalysts,^74^ in particular for the sluggish alkaline HER.^58^ Other reviews have provided discussions relating the limitations and challenges of applying conventional volcano plots to describe HER and other electrocatalyst activities.^70,75,76^

## 2 Assessing Catalyst Performance: Benchmarking and Best Practices

**Figure 4:**
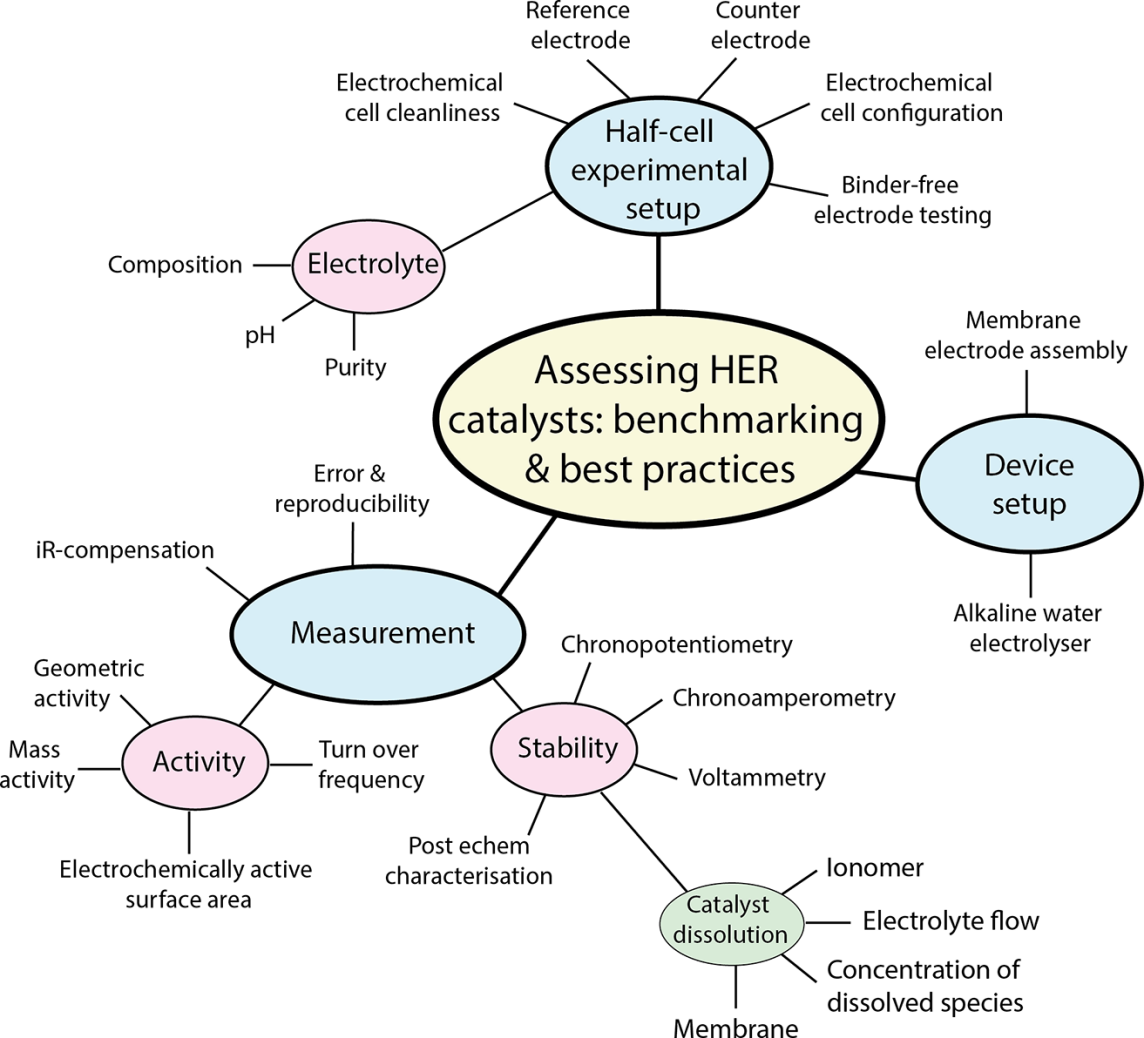
Schematic to highlight the key considerations that a researcher should factor into the design of their HER catalyst performance measurements.

The most commonly reported and compared metric for HER activity from half-cell measurements are *i–V* response (e.g., cyclic voltammetry or linear sweep voltammetry), overpotentials or onset potentials at a given current density. Electrochemical active surface area (ECSA) normalized current densities, turnover frequency (TOF), Tafel slope, exchange current density and short-term durabilities determined by chronoamperometry (CA), chronopotentiometry (CP) or cyclic voltammetry (CV) are also regularly reported and discussed. These measurements are often accompanied by *ex situ* or *in situ* materials characterization including electron microscopy, X-ray diffraction, dissolved ion quantification, and other spectroscopic methods. In this section we give a brief overview of these electrochemical methods. Subsequently, we review best practices for half-cell measurements and stability measurements. Here, our intention is that through this review of recent reviews, publications, perspectives, and editorials we can provide a summary of the various best practices and considerations for data acquisition and analysis for half-cell based HER catalysts. Finally, we discuss device testing, including membrane electrode assemblies and alkaline water electrolyzer testing protocols, providing a review of testing methods as well as examples of precious metal free catalysts that have been translated into such device tests. Figure 4 is a schematic to highlight the most critical factors which should be considered for benchmarking HER catalysts, each of which are reviewed in this section.

### 2.1 Activity Metrics

**Figure 5:**
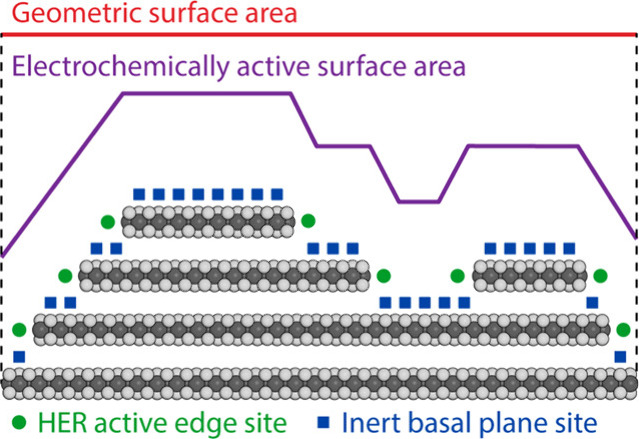
Cross-sectional schematic representing a MoS_2_ electrocatalyst highlighting the difference between geometric surface area and electrochemically active surface areas. The active sites of the catalyst (green dot) are also shown to highlight that such surface area normalizations do not necessarily allow for assessment of intrinsic activities between electrodes. Reproduced with permission from ref (79). Copyright 2014 American Chemical Society.

Several different metrics can be used for assessing and comparing electrocatalyst activities which have been reviewed elsewhere.^78−82^ Geometric area normalized current densities at given overpotentials are the most frequently reported performance metrices in half-cell measurements whereby the current collected in a CV or linear sweep voltammogram (LSV) is normalized to the surface area calculated by the geometrical dimensions of the electrode (Figure 5).^78^ Typically, geometric area current densities are reported with units of mA cm^–2^_geo_. In an attempt to enable comparison between different electrocatalysts and testing modes, the applied potential (or overpotential) to reach 10 mA cm^–2^_geo_ is also widely reported.^83^ While such geometric area normalized activities may be most commonly reported, the metric itself has very little scientific meaning in terms of actual activity of a material unless the catalyst has an atomically perfectly flat surface. Most precious metal free, (and state-of-the-art Pt/C), catalysts are heterogeneous structures with complex surfaces including steps, pores, defects, and interfaces. Ideally, one should strive to normalize the current with the electrochemically active surface area (ECSA) for each catalyst so that electrochemically inert surfaces, volumes, and materials can be excluded (Figure 5). However, experimental determination of ECSA for precious metal free catalysts is neither trivial nor has any consensus been agreed on methods for best estimates. For Pt based HER catalysts hydrogen adsorption/desorption can be used to accurately measure the ECSA. Such measurements are neither reproducible nor universally applicable in precious metal free catalysts owing to their varying binding energies toward hydrogen and protons. The most commonly utilized and optimized methods for precious metal free electrocatalyst ECSA measurements require the underpotential deposition of a metal (Cu or Pb), CO stripping, redox peak integration, double layer capacitance measurements, or, using electrochemical impedance spectroscopy (EIS). Each of these techniques have limitations and none are universally applicable. However, we recommend reporting ECSA measurements provided the methodologies are clearly described so that geometric area normalized current densities can be contextualized against active site densities to some extent. In particular, we highlight the potential advantages of utilizing EIS which can enable assessment of ECSA under the relevant applied electrochemical potential for the HER.

It Is also essential that the experimental section of published literature should clearly describe the physical properties of the working electrode for the readers to understand how the current densities were computed. For example, if an RDE is utilized one should clearly state the material (graphite, gold or other) and the radius of the disc. In the case of catalysts grown on high surface are substrates (e.g., foams or mesh electrodes), it is quite uncommon to quantify catalyst surface areas. We suggest that every effort should be made to isolate and quantify the electrocatalyst surface areas. It is also essential to describe how the electrodes were conditioned, activated and which CV or LSV cycles are presented since the activities will potentially fluctuate over time.

While the precious metal-based literature regularly reports mass activities (current normalized to mass of catalyst, with units of A g^–1^), this is rather uncommon in the precious metal free literature. However, we recommend that analogous to area normalized activities (ECSA and geometric) mass activities should also be accurately determined wherever possible. These measurements are essential for developing insight regarding the intrinsic activity of catalysts, or to provide like-for-like comparisons between different electrodes prepared within a study. In the case of RDE where a catalyst ink is usually deposited onto the working electrode, catalyst mass reporting is relatively straightforward, provided that the publications report all the relevant data (e.g., ink recipe, volume deposited etc.). In the case of self-supported or substrate grown catalysts, more advanced *ex situ* techniques such as energy dispersive X-ray spectroscopy (EDS) and X-ray fluorescence (XRF) can be utilized to determine the mass of active material if nondestructive methods are a must.^84,85^ Simple mass balance before and after the durability tests also can be utilized if highly accurate balances are accessible. The most accurate catalyst mass determination is perhaps through ICP-MS measurements of dissolved catalysts/electrodes. However, such analysis requires reliable duplicate sample preparations so that one can be utilized for electrocatalysis and the other for catalyst loading analysis. It is also recommended that effects of mass loading on the catalytic activities be carried out to determine optimal catalyst layer thickness on the working electrode. A common argument for higher loadings of precious metal free catalysts compared to precious metal catalysts is that materials costs are insignificant in comparison to PGM catalysts. While this is the case for some materials, we postulate that this may not always be a correct assumption. Furthermore, high catalyst loadings exhibit higher apparent HER activity, but can potentially suffer from mass transport limitations when incorporated into a working device.^52^ Similarly, if any binders such as Nafion are utilized to affix HER catalysts on the working electrode, it is recommended that the effects of Nafion content in the catalyst ink also be investigated. It is well established that Nafion and other binders significantly affect the conductivity and mass activities of catalyst layers.^86−88^

To understand the kinetics and HER reaction pathways when utilizing various electrocatalysts in different electrochemical environments researchers most commonly determine the turnover frequencies (TOF) and Tafel slopes.^52,81^ TOF and Tafel analysis require elucidation of mass-transport limitations and electrical resistance from activities. The potentials should be *iR* corrected as discussed in Section 2.2.6 and methodologies clearly described as per recommendations from the research community.^78,85,89−91^ Tafel slopes are computed by plotting the overpotentials (η) against log values of current densities (*j*). The Tafel slope (2.303*RT*/α*nF*) in eq 11 (where *R* is the gas constant, *T* is the temperature in Kelvins, α is the charge transfer coefficient, *n* is the number of electrons transferred to generate one product molecule and *F* is the Faraday constant) is commonly then used to elucidate reaction kinetics of the associated electrochemical reaction. The current density at equilibrium potential (exchange current density, *J*_0_) are the most useful and most often used parameters in describing reaction kinetics. Anantharaj et al., discuss appropriate methods to report and utilize Tafel slopes,^89^ and we urge the research community to use appropriate rigor to report and caution to utilize the matrices for analysis:
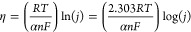
11

Tafel slope analysis should always be contextualized with other activity and kinetics metrices including TOF, exchange current densities, ECSA and other measurements that help articulate reaction mechanisms and reaction kinetics. If used on their own, Tafel slopes become ambiguous and often lead to erroneous conclusions.^92,93^ For example, symmetry factors must be used to effectively correct Tafel slopes, but accurately assigning the symmetry factor is inherently challenging. Thus, it is important to contextualize Tafel slope discussions to methods used and other kinetics parameters. In 2014 Kichigin and Shein presented a theoretical derivation on how EIS is used to probe the rate-determining step and reaction mechanism for the HER at different electrochemical conditions.^94^ Their method also enables an extraction of transfer coefficients, rate constants, and hydrogen coverages. An example of the synergy between Tafel analysis and EIS is presented by Galyamin et al., where they show direct correlation between the Tafel slopes determined from voltametric techniques and the equivalent circuit elements from the EIS analysis.^92^ Such cross-examining approaches are highly encouraged to find methodologies that can be universally adopted across various families of electrocatalysts. However, rigorous validations need to be presented and detailed methodologies reported to test the added value of such methods toward more accurately determining Tafel Slopes and ECSAs.

Similar to Tafel Slopes and ECSA determinations, TOF calculations also require accurate and detailed knowledge of catalyst structure. For crystalline electrocatalysts, one should first accurately determine the crystal structure complemented with other materials characterization tools that enable determination of structural and stoichiometric properties. Typically, assumptions must then be made regarding which site (e.g., metal atom or nonmetal atom) should be considered active. For example, on the surface of a CoP nanoparticle, one might calculate the average surface area, and use the crystallographic structure to estimate the number of Co atoms present on the surface of the particle. Knowing the catalyst loading on the electrode, one can therefore estimate the total number of Co atoms exposed to electrolyte. It is critical that such calculations are explained fully (either within the methodology or within the Supporting Information), and that any assumptions made in the calculation are fully discussed. Finally, it is critical that such estimates provide the most conservative estimate (i.e., the highest possible number of active sites) to calculate the TOF.^81,90,95^ As Anantharaj et al. discuss TOF calculations for various types of HER catalysts, the most common equations used are eqs 12-14 where *j* is current density, *N*_A_ is the Avogadro constant, *F* is Faraday constant, *n* is the number of electrons transferred to generate one molecule of the product, Γ is the surface concentration of active sites, *i* is the current, *A* is the area of the electrodes, and *x* is the number of moles of active sites available for catalysis.^81^ It is acknowledged that accurate determination of these intrinsic activity parameters is difficult and often requires estimations, but we encourage the community to carefully make conservative estimates to report TOF values:
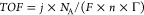
12
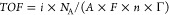
13
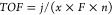
14

Like half-cell measurements, due care should be taken to report the MEA performance metrices. Detailed information on how polarization curves were generated should be provided in the experimental section. Potentiostatic or amperometric holds at regular intervals should be used and average values from >30 data points utilized after the performance stabilizes to generate pole curves.^96−99^ Such practice ensures that the electrolyzer reaches steady state performance at each potential (or load) hold. Where possible, one should also strive to assess *iR* free potentials through high frequency resistance (HFR) measurements. More details on best practices and recommendations regarding equipment assembly and hardware selections for half-cell and MEA measurements are discussed in subsequent sections.

### 2.2 Electrochemical Testing Setup: Half-Cell

Electrochemical measurements are inherently sensitive to contaminations and unintended side reactions. The effects are especially pronounced when assessing low catalyst loadings (μg–mg) and ultralow activities (μA–mA) as is typical for half-cell measurements. A range of methodologies have been utilized and protocols have been proposed over the years to ensure that due care is taken in ensuring the analytical equipment is ultraclean and methodologies are comparable when assessing electrochemical performances and durabilities.^100,101^ Indeed, the choice of test vessels, electrolytes, electrodes and configuration of these components into the analytical system need to be carefully considered when assessing activities and durabilities in different cell configurations, electrochemical environments and modes of assessments.^78^ In this section we review these various proposed best practices of relevance to HER catalyst screening.

#### 2.2.1 Electrochemical Cell and Cleaning

With regards to a rotating disk electrode (RDE) setup for HER catalyst activity measurements, Wei et al. provide discussion regarding the rationales for the choice of various components (e.g., choice of appropriate reference and counter electrodes, and the purity of the electrolyte) suitable for assessments of precious metal free electrocatalyst activities.^78^ There are also recent editorials and perspectives discussing rationale behind the choice of components and conditions while assessing HER.^85,91,102−104^ Much of this discussion is focused on PGM catalysts, but these good practices can be equally applicable to precious metal free HER catalysts as well. The consensus regarding the choice of vessels is that ultraclean glassware should be used for electroanalysis in acidic electrolyte and chemically resistant polymer vessels (e.g., Teflon or PEEK) for analysis in basic electrolytes. Glassware is preferred under acidic conditions because the homogeneous surface that is resistant to abrasion and chemical corrosion. Additionally, transparent glass allows direct observation into the reaction volume during set up and while electrochemical reactions are taking place which can help with bubble management. However, ions of constituent elements such as Si, Al, B, Ca, and Fe leach out from glass when exposed to an alkaline environment which can contaminate the electrocatalyst.^105^ Using polymeric materials mitigates the possible contaminant issues but polymers are opaque and also prone to abrasion. It is likely that the resulting rough surface can trap contaminants during cleaning or evolved gases during electrocatalysis.^103^ Thus, it is essential to ensure all reaction vessels are abrasion free and clean when setting up HER electrochemical testing.

Alia and Danilovic provide a detailed protocol on cleaning glassware for acidic half-cell reactions for water electrolyzer and hydrogen/oxygen fuel cell reactions.^100^ They adapted the procedure developed by Garsany et al. for fuel cell half-reactions,^106^ and such glassware cleaning protocol has also been recommended for cleaning polymer electrolyte vessels to be used for alkaline HER with due consideration for compatibility of the polymer in the cleaning solutions.^78^ The general protocol consists of soaking reaction vessels and components consecutively in strong acid and strong base solutions overnight followed by boiling in fresh DI water several times.^78,100^ Additionally, all reaction vessels and components are recommended to be stored submerged in ultrapure DI water to avoid contamination from air borne impurities.^100^

#### 2.2.2 Electrolyte Purity

The purity of electrolyte itself is critical, especially for alkaline HER. Marquez et al. discuss the importance of ensuring appropriately high electrolyte purities are used throughout electrochemical analysis and methodologies are provided to purify alkaline electrolytes.^107^ In general, the highest possible grades of acid and alkaline electrolytes should be used, and where necessary, appropriate cleaning procedures need to be followed to remove critical contaminants such as Fe in alkaline and precious metals in acid electrolytes prior to commencing electroanalysis.^108^

#### 2.2.3 H_2_ Gas Saturation

When evaluating the HER activity, it is crucial to saturate the electrolyte with H_2_ gas. Otherwise, the partial pressure of H_2_ entered into the Nernst equation for HER and can thus shift the potential of the H_2_-redox couple.^78^ Assessing the activity of a HER catalyst in an H_2_ under-saturated electrolyte or even an Ar-saturated one can cause HER currents to start anodically of 0 V versus RHE.^109^ Similarly, when calibrating the reference electrode to the RHE scale using Pt-electrodes, it is essential to keep the electrolyte saturated with H_2_ gas to ensure the equilibrium potential is measured in accordance with the Nernst equation under standard conditions.^49,78^

#### 2.2.4 Counter and Reference Electrodes

**Figure 6:**
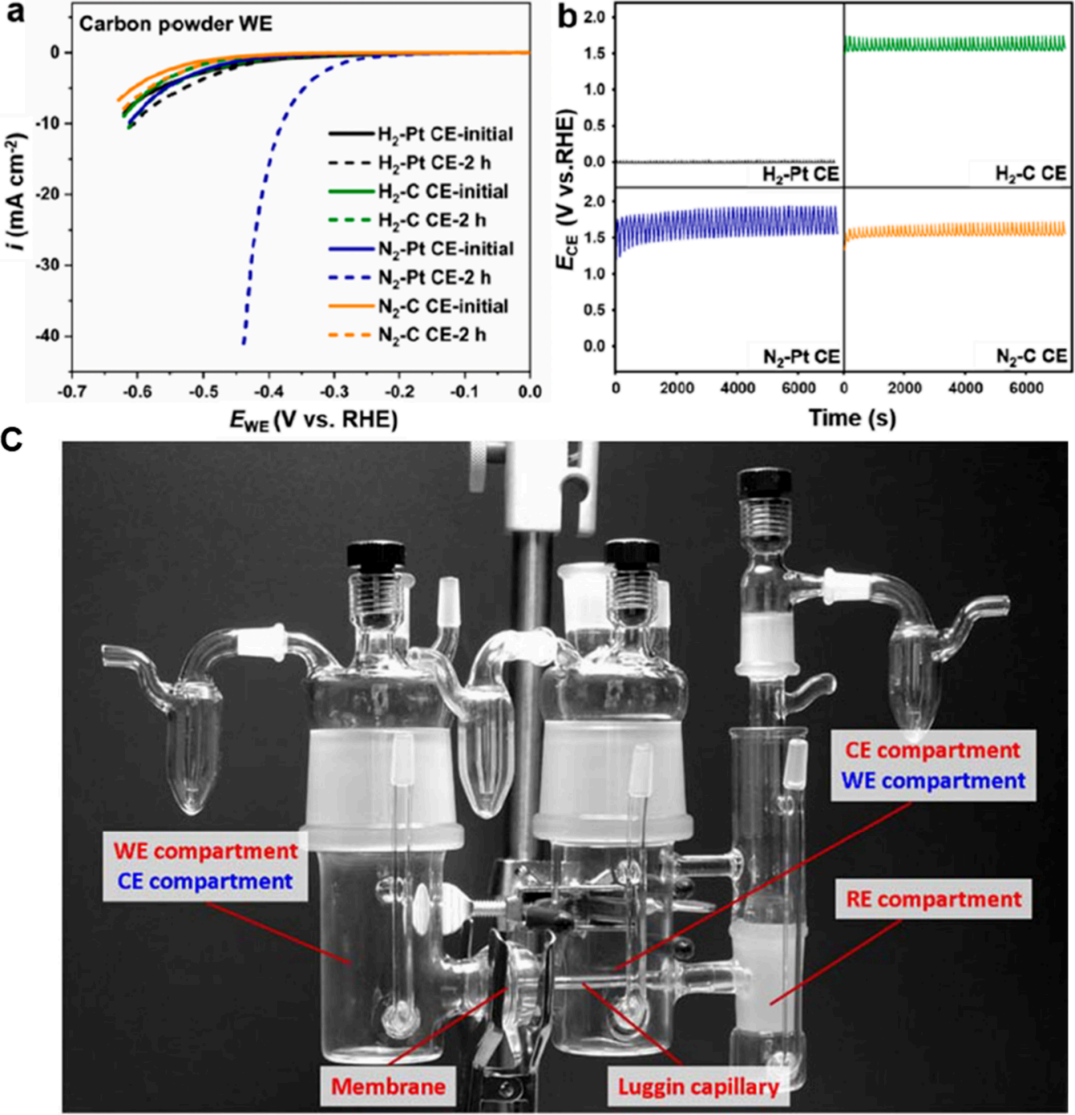
(A) Influence of various counter electrodes (Pt, carbon) and saturating gas (N_2_, H_2_) on the HER activity of carbon powders working electrodes in an undivided cell. The HER activity remains almost the same after 2 h of CV cycling at 10 mV s^–1^ when the electrolyte is saturated with H_2_ irrespective of whether the counter electrode is graphite or platinum, but HER activity dramatically improves when platinum counter electrode is utilized under N_2_ saturated electrolyte.^113^ (B) The counter electrode potential for each experiment. Note at potentials >1.5 V OER is taking place at the counter electrode.^113^ (C) A divided RDE cell that separates working, reference and counter electrode.^111^ Reproduced with permissions from ref (113). Copyright 2023 American Chemical Society; ref (111). Copyright 2016 American Chemical Society.

Counter and reference electrodes are the most common sources of electrolyte contaminants during HER if improperly selected and deployed. Pt, Au, Ni and graphite are the most common counter electrodes deployed during HER. Metallic counter electrodes are generally not recommended for either undivided or compartmentalized cells. Jerkiewicz discusses in detail the complexities of Pt as a counter electrode for electroanalysis^110^ and discusses earlier work to demonstrate the dissolution and redeposition on the working electrode under HER conditions.^111^ Such effects are known to lead to erroneous HER activities and long-term durabilities, especially when evaluating the activities of precious metal free HER electrocatalysts. To evidence this, a Pt counter electrode was deployed during the assessment of precious metal free carbon powder HER catalysts. Although the precious metal free catalyst activity was shown to be relatively stable during chronoamperometric, chronopotentiometric or during CV measurements, it was found that this was due to the deposition of dissolved Pt ions onto the working electrode which counteracted the precious metal free HER catalyst degradation (Figure 6A). Beyond Pt, other metals such as Au and Ir counter electrodes also dissolve and redeposit at the working electrode. The apparent effect on HER activity may not be as drastic as Pt, but the dissolution and redeposition can still interfere with the electroanalysis. Using high surface area carbon based counter electrodes can mitigate the effect of HER active precious metal counter electrodes. However, the generation of carbon monoxide and other carbon species at the counter electrode can influence the performance of the working electrode if such species migrate to the working electrode. This effect is likely to be especially pronounced at high current densities and long-term durability tests. Recent reports have demonstrated that the optimal configuration is to separate carbon-based counter electrodes (graphite or glassy carbon) from the working electrode chamber with a glass frit (Figure 6C).^91,111,112^

The dissolution of the counter electrode and possible contamination on the working electrode is known to accelerate under inert gas saturated electrolyte.^113^ Under such inert environments, the electrochemical reaction occurring at the counter electrode is the oxygen evolution reaction (OER) requiring high potentials (Figure 6b). At these high potentials, metal oxides or oxides of carbon form at the counter electrode which undergo dissolution and can therefore potentially be electrodeposited at the working electrode.^114^ Conversely, a hydrogen saturated electrolyte can enable the hydrogen oxidation reaction (HOR) at the counter electrode which occurs close to 0 V vs RHE (Figure 6b) in the case of Pt counter electrodes, mitigating the dissolution of counter electrode and the resultant cross-contamination of the working electrodes. Cui and Sheng have recently demonstrated the effect of saturating the electrolyte with hydrogen gas and the relative position of counter electrode to working electrode within the electrochemical vessel (Figure 6).^113^ They demonstrate that saturating electrolyte with hydrogen prevents counter electrode dissolution and separation of working electrode chamber from counter electrode chamber, as shown in Figure 6c, can mitigate the migration of dissolved species to the working electrodes. It must be emphasized, despite commonly accepted notion regarding kinetics being extremely fast rendering mass transport limitations in HER/HOR reactions insignificant, one must ensure the electrolyte is sufficiently saturated prior to electrochemical measurements such that equilibrium potentials are achieved within the Nernst equation. Zheng et al., have demonstrated that mass transport limitations can indeed influence even HER/HOR reactions due to sluggish H_2_ diffusion away from the electrodes into the bulk electrolyte.^78,109^

Reference electrodes should also be carefully selected taking into consideration the electrolyte composition and pH. A reversible hydrogen electrode has been recommended where possible.^78,100,103^ However, Oshchepkov cautions that even reversible hydrogen electrodes potentials can also substantially change if the concentration of hydrogen is not sufficiently maintained and/or if dissolved species from either working or counter electrodes reach the reference electrode; Pt especially can further deviate from the original reference electrode potential.^103^ Wei et al. provide a detailed rationale for selecting mercury based reference electrodes, Hg/HgO or Hg/Hg_2_SO_4_ for acidic systems and Hg/HgO for alkaline electrolyte, and converting all potentials to RHE.^78^ Anantharaj et al. have very recently demonstrated the need to use appropriate reference electrodes for different pH regimes, and the need to regularly calibrate the reference electrodes as they drift away from ideal scale over time.^80^ They observe that across the HER literature, silver–silver chloride (Ag/AgCl) reference electrodes are most widely used as well as responsible for the majority of erroneous reports deviating as much as 70 mV from the thermodynamic potentials. Similarly, Zamora et al. also show that the reference electrode needs to be calibrated periodically; the frequency of calibration depends on frequency and mode of usage (duration and types of experiments) but they recommend calibration every time new electrolyte is used in experiments.^115^

#### 2.2.5 Binder-Free Electrode Testing

Half-cell tests that are not based on RDE, in most-cases where the catalyst is either grown on a substrate or as self-supported electrodes, should also follow the recommendations discussed above. Additionally, due care should be taken to ensure the substrates and connectors (e.g., alligator/crocodile clips for connection to the potentiostat) do not release contaminants during electrocatalysis. Zheng et al. have recently described best practices when deploying foam-based electrodes for HER in non-RDE half-cell measurements.^116^ Similarly, Jin et al. have reviewed strategies deployed to assess HER electrocatalysts for high current densities in a half-cell setup.^117^ It is generally not advisible to use RDE measurements for assessing activities at large current densities (>200 mA cm^–2^) owing to the severe mass transport limitations. Instead, in recent years other innovative architectures such as inverted RDE,^118−120^ microelectrodes,^121,122^ floating electrodes^123^ and gas diffusion electrodes have been proposed to bridge the gap between RDE and MEA.^124^

Given the variety of precious metal free HER catalysts that have been reported in recent years, it is almost an impossible task to develop a universal methodology to assess the entire spectrum. As an exercise to develop a uniform method, McCrory et al. analyzed thin film catalysts prepared via electrodeposition and/or sputter deposition on glassy carbon electrodes for both acid and base toward HER.^83^ They identified multiple precious metal free transition metal based HER catalysts that exhibited promising activity and short-term durabilities. A similar benchmarking exercise has not been undertaken for the plethora of powder catalysts. Despite the enormity of the task to analyze and compare the activities of myriad types of precious metal free catalysts, reproducibility of the data and comparison of catalytic properties can be facilitated if the community ensures recommended good practices are followed strictly (refs (78, 80, 85, 91, 100−103, 107, 110−113, 115, 116, and 125)).

#### 2.2.6 *iR* Compensation

**Figure 7:**
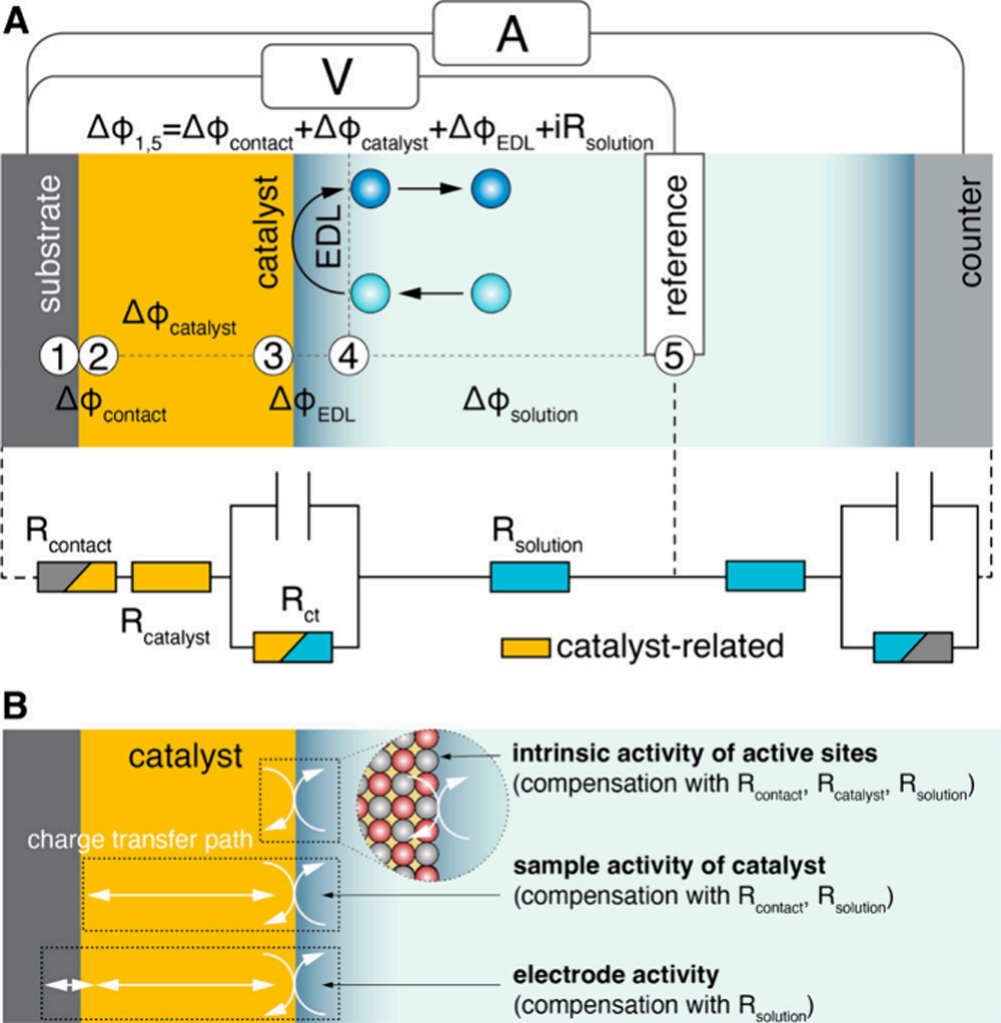
A) A schematic of the resistances that contribute to potential drops in a simplified three-electrode half-cell electrochemical cell with a catalyst-loaded working electrode, together with its equivalent circuit diagram. The numbers (1–4) indicate points of contact between distinct regions in the system (e.g., substrate-catalyst). B) Identifies three “regions of interest” as marked by the dotted boxes (e.g., catalyst-solution) highlighting the resistances of interest for intrinsic activity measurements, sample activity of catalysts, and electrode activity measurements. Critically, for catalyst optimizations the conductivity of the catalyst layer is also relevant to study, whereas for an electrode design, the substrate-catalyst contact resistance likewise demands attention. Reproduced with permission from ref (126). Copyright 2023 American Chemical Society.

*iR* compensation is another critical consideration when running half-cell experiments to facilitate HER catalyst activity comparison across different electrochemical setups and laboratories. The compensated data in principle eliminates variations emanating from the distance between electrodes and properties of the separators. However, complete elimination of such effects requires full compensation (100%) of the ohmic drop. Currently, standard practice is a partial compensation, typically 85%, of the measured value. An insightful recent perspective by Zheng on this topic proposes dividing electrocatalyst research into different “types of measurements” to allow more meaningful *iR* compensations.^126^ For example, assessing the intrinsic activity (RDE), electrode activity (single cell), and industrial activity (stacks) potentially each require different considerations with regards to cell configurations as well as *iR* compensation. Such distinctions help elucidate the different contributions to the *iR* drop from specific regions in the substrate-catalyst-electrolyte system. Proposed by Zheng, specific experiments enable compensation of specific and distinct *iR* drops. Figure 7 shows the regions contributing to the *iR* drop in the substrate-catalyst-electrolyte system in a three-electrode setup alongside the equivalent circuit diagram.

All but one of the resistances in Figure 7 are adequately quantified by EIS. Typically, EIS measurements are run between 100 kHz and 0.1 Hz, with a voltage perturbation of 10 mV.^78^ The ideal resistance to compensate is dictated by the magnitude of the impedance where the phase angle is closest to zero, (i.e., where the imaginary part is closest to zero). This can be read directly from the high frequency range in a Nyquist plot. Critically, the measured high frequency resistance (R_HFR_) is a sum of multiple resistances, and not purely the electrolyte resistance (except in some RDE measurements where the stub and catalyst are one homogeneous material). This impedance is made up of three contributions: the electrolyte resistance R_solution_, the contact resistance between the catalyst and the support/substrate, R_contact_, and the resistance within the catalyst layer R_catalyst_.

Zheng has several suggestions to optimally utilize *iR* compensation in electrocatalysis. Here, we relate *iR* compensation in reference to the HER catalysts. Critically, the type of *iR* correction should depend on the type of measurements you are performing: (1) When studying the intrinsic activity of a catalyst, a 100% *iR* compensation should be used to eliminate any contribution from electrode-catalyst-electrolyte (R_contact_ + R_catalyst_ + R_solution_). This is done by compensating fully for R_HFR_ measured using the electrode-catalyst assembly. (2) Meanwhile, there is plethora of work focused on optimizing the overpotential of a catalyst-coated substrate (e.g., Ni-foam for A-WE and carbon cloth/paper for PEM-WE). For these studies, the interaction of the substrate and the catalyst is important and should be optimized instead of neglected. Thus, only the electrolyte resistance should be quantified and compensated. R_solution_ can be isolated and measured as the R_HFR_ using only the bare substrate, enabling one to compensate for 100% of the ohmic loss in the electrolyte. (3) For benchmarking catalyst materials but disregarding the electrode-catalyst interactions, 100% compensation for the electrolyte *iR* drop should be measured but in addition, a measurement of the substrate-catalyst contact resistance (R_contact_) should be performed, such as, using a Kelvin four-wire resistance measurement. Otherwise, compensating for the full R_HFR_ using the electrode-catalyst assembly may produce erroneous results. Some catalysts are efficient catalysts but poor conductors, and benefit heavily from a mixing with conductive material.^127^ A single compensation of R_catalyst_ on its own therefore may mask such effects.

### 2.3 Electrocatalyst Stability

To withstand the various operating conditions, such as fluctuations in operating potential, load (current densities), temperature, differential pressure between the cathode and anode, and shut down cycles, precious metal free HER catalysts should stably work without activity decay or dissolution. The field therefore requires rigorous protocols for evaluating the catalyst stability. This section discusses the methodologies for assessing catalyst in a 3-electrode setup. We start with activity decay/service time measurements, including CA, CP, which are effective methods for preliminary test but insufficient. Further, we discuss measuring catalyst dissolution by inductively coupled plasma (ICP) methods. Various cells for ICP measurements are reviewed, and then the factors that may influence the dissolution measurements, including the concentration of dissolved species, the use of ion-exchange membrane, ionomer and potential control, are discussed. Finally, we review the post-mortem analysis typically conducted alongside stability testing.

#### 2.3.1 Electrochemical Stability Measurements

Chronoamperometry (CA), chronopotentiometry (CP) and potential cycling serve as the most effective initial methods for evaluating the performance loss of a catalyst. Degradation of catalysts can, however, occur through a multitude of processes, including delamination of the catalyst or the poisoning of active sites, and, additionally, loss of activity can be either temporary or permanent.^128^ As Risch has exemplified while studying the OER, investigating degradation necessitates the coordination of multiple measuring techniques, including nonelectrochemical methods, such as gravimetric techniques, which can provide further insight into degradation mechanisms. Risch also advocates that degradation studies should focus on conducting measurements until catalyst end-of-life, providing a more thorough understanding than merely stating the stability of a catalyst in terms of the hours tested. This approach would enable researchers to extrapolate catalyst stability measurements to more accurately provide comparisons between catalysts, and help transition the field away from sweeping stability claims for X hours or Y cycles of stability.

The most popular electrochemical methods are CP and CA. Changes in the overpotential during CP and the current during CA measurements have been widely used to quantify stability. The service time measured by CP has also been used to reflect catalyst lifetime. We direct readers to a recently published review for more examples.^129^ Activity loss measured by CA and CP are essentially the same, but CP is visually more deceiving than CA, which is because current is exponentially related to potential (*i* ≈ exp (*V*)). Assuming a Tafel slope of 30 mV/dec, a potential increase of 30 mV might appear to be small in CP, but it is actually a 10-time decrease in activity.

Beyond CP or CA measurements, triangular or square wave cyclic voltammetry have been used to assess HER stability in 3-electrode half-cell measurements. However, this technique may not effectively capture time-dependent phenomena similar to the demonstration from Kneer et al. for platinum oxide in proton exchange membrane (PEM) fuel cells.^130^ Indeed it was shown that the degradation of the catalyst was not tied to the number of cycles but rather to the time spent at specific potentials. Therefore, the number of voltage cycles alone may be inadequate for assessing stability.

Beyond the more simplistic CP, CA and CV-based stability measurements, the stability number (S-number) represents an alternative metric for comparing catalyst stabilities. The S-number, which is typically deployed for assessing the OER electrocatalysts in 3-electrode measurements, normalizes the number of generated O_2_ gas molecules per atom of electrocatalyst dissolved. This metric has recently gained traction as a useful method to quantify stability during the operational phase of OER catalysts.^131^ The S-number is, however, rarely reported as a stability metric for the HER. We postulate that this metric could be widely used for a wide range of electrocatalytic measurements, including the HER.

In summary, each electrochemical method exerts a different electrochemical stress while probing the stability of catalysts. To recommend a superior one among all these techniques, a future study on the stability of the same catalyst measured by different methods is needed. Note the mentioned methods are insufficient methods that can be only used as a preliminary test for assessing catalyst stability. For example, the observed “stable” signal in such measurements could be simply due to an increase in roughness factor, caused by the corrosion of catalyst, especially with a thick catalyst layer or high catalyst loadings. Only once the catalyst layer has completely corroded away, might the electrode fail by such metrics. For a rigorous stability test, we recommend a low catalyst loading, or a better practice is to use ICP for quantifying the corroded products in electrolyte, which is discussed in the following section.

#### 2.3.2 Inductively Coupled Plasma Dissolution Studies

ICP is the most commonly deployed technique to measure catalyst dissolution during electrocatalytic stability measurements. ICP allows for multielement analysis, while electrochemical quartz crystal microbalance (EQCM) only measures total dissolution of catalyst/catalyst layer, not element-specific. Kasian et al. and Cherevko et al.^132,133^ have reviewed the typical ICP instruments and electrochemical cells deployed for measuring the concentration of dissolved catalyst species in electrolyte, and their applications for various reactions. Here we briefly discuss those setups, and some exemplary measurements of HER catalyst dissolution.

ICP has a high sensitivity (down to ppt level). Two ICP instrument configurations are typically used for such measurements based on the detector: optical emission spectrometry (ICP-OES) and mass spectrometry (ICP-MS). In general, ICP-MS is more sensitive than ICP-OES. Depending on the element, the detection limit of ICP-MS can be as low as ppt; in contrast, ICP-OES is ppb. The tolerance for total dissolved solids (TDS) of ICP-MS is up to 0.2%, lower than ICP-OES (up to 30%). This limits ICP-MS to electrolytes of lower concentrations (such as 0.1 M HClO_4_, 0.05 M H_2_SO_4_) compared to ICP-OES, which might be suitable for studies in aggressive electrolytes, such as such as 1 M KOH and seawater (TDS ≈ 3.5%).^134,135^

**Figure 8:**
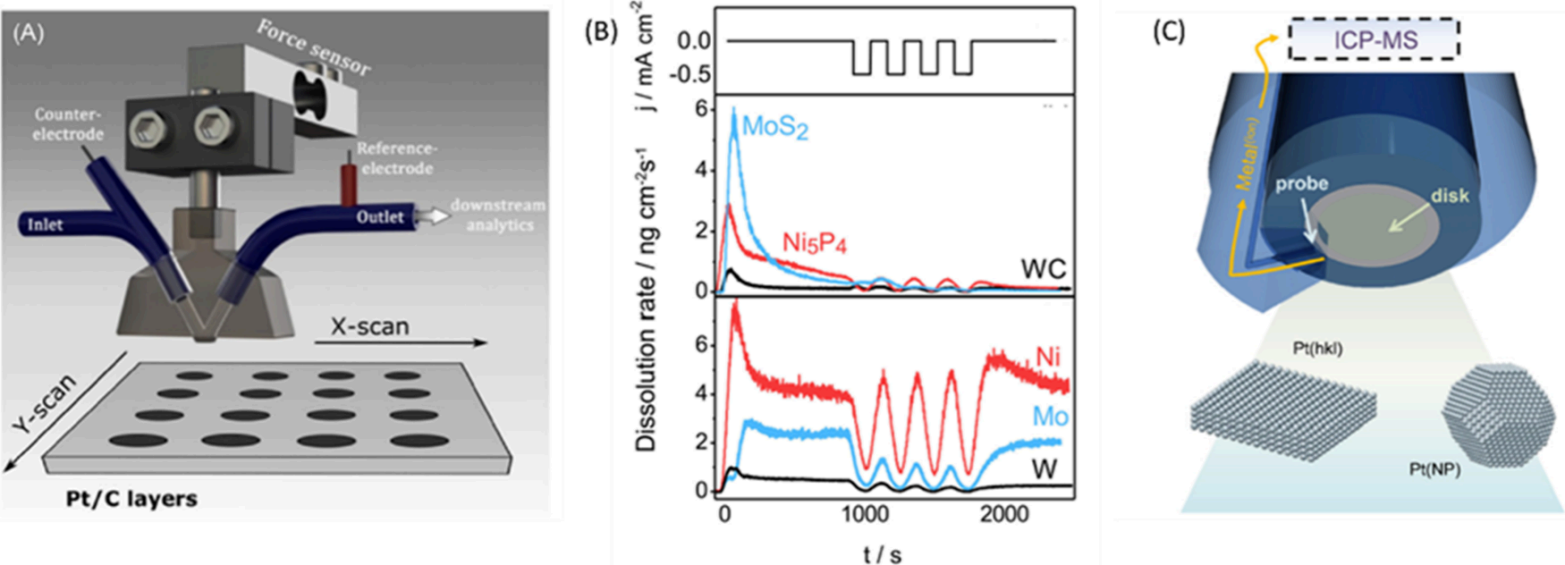
(A) Schematic illustration of the *in situ* SFC-ICP-MS setup. (B) The dissolution profiles of WC, MoS_2_, Ni_5_P_4_ HER catalysts recorded applying electrochemical protocol imitating shutdown conditions of an electrolyzer. (C) Schematic of an ICP-MS probe positioned in vicinity to the electrode surface, for *in situ* ICP measurement. Reproduced with permissions from ref (132). Copyright 2019 Wiley-VCH; ref (133). Copyright 2022 Elsevier.

There are three popular electrochemical setups for measuring catalyst dissolution: *in situ* ICP measurement coupled with a flow cell (Figure 8A), *in situ* ICP measurement coupled with a stationary cell (Figure 8C), and *ex situ* ICP measurement in a stationary cell.

Various *in situ* ICP measurements coupled with a flow cell have been developed.^136−140^ An example is the scanning flow cell ICP-MS (SFC-ICP-MS) in Figure 8A.^141,142^ This cell has a V-shaped flow channel, through which fresh electrolyte is continuously pumped from an electrolyte reservoir to the ICP-MS, to achieve a 100% collection efficiency of dissolved products. The ratio of one-way electrolyte flow rate to working volume is maximized, and the flow channel is optimized to allow for a uniform flow profile. The dissolved species are considered instantly removed away from electrode surface, without any accumulation within the electrolyte.^141−143^ Ledendecker et al. studied the dissolution (W, Ni, Mo, Co) of metal carbides (WC), sulfides (MoS_2_), phosphides (Ni_5_P_4_, Co_2_P) for acidic HER by performing start–stop cycles, between open circuit potential (OCP) and −0.5 mA cm^2^_geo_, mimicking on–off conditions of an electrolyzer (Figure 8B).^144,145^ It was found these materials undergo dissolution at OCP, but negligible dissolution under HER relevant potentials. Alternating between OCP and HER relevant potentials was also used for monitoring WC, WO_3_ and W HER catalysts in acid, which also only dissolved under OCP.^145^ Holzapfel et al. monitored the dissolution of [Mo_3_S_13_]^2–^ clusters in acid by applying 25 CV cycles from 0 to −0.25 V vs RHE at 100 mV s^–1^ scan rate, followed by start/stop current holds, shifting from HER currents of −55.55 mA mg^–1^_cat_ (−4.7 mA cm^–2^_geo_) and −111.11 mA mg^–1^_cat_ (−9.2 mA cm^–2^_geo_) and 0 V vs RHE voltage holds, respectively.^146^ Schalenbach et al. observed the selective leaching of Mo at NiMo alloy catalysts for alkaline HER, by using the following two procedures: (1) a constant potential of −0.2 V vs RHE, followed by two slow CVs at 2 mV s^–1^ in a range from −0.35 to 0.4 V, and afterward OCP for 500 s. (2) accelerated stress test (AST) protocol consisting of 3 CVs in the same potential range at 10 mV s^–1^ before and after 50 AST cycles at 200 mV s^–1^.^147^ Escalera-López et al. employed the combinational procedures of CV cycles and start–stop cycles from −1 mA cm^–2^_geo_ to 0 V vs RHE to measure the phase-dependent dissolution of MoS_2_ catalysts for acidic HER.^148^ By quantifying the dissolved catalysts, they calculated the HER stability number (the number of produced H_2_ gas per atom of dissolved catalyst) to provide a quantitative stability comparison. Göhl et al. assessed the stability of TiC, VC, NbC, TaC and WC in acid using potential cycling over a broad potential window (−0.2–1.5 V vs RHE at 3 mV s^–1^) and TiC, NbC, and TaC showed dissolution within the HER potential window.^149^

*In situ* ICP measurements coupled with a stationary cell can be performed by positioning the ICP inlet probe in the vicinity of working electrode (∼1 mm away from electrode surface), in a conventional 3-eletrode cell, as developed by Lopes et al. (Figure 8C).^150,151^ This setup conceptually resembles the classic rotating ring disk electrode (RRDE) setup, where the ring is replaced by a ICP probe. Since the collection efficiency is not 100%, calibration of the collection efficiency is required to perform quantitative analysis. Furthermore, owing to the stationary nature of this nonflow cell, the dissolved species accumulate over time (the potential consequences of which are discussed below). To date, this setup has predominantly been deployed for monitoring OER catalysts.

Owing to the complex and expensive setup required to perform *in situ* ICP measurements, *ex situ* ICP measurement in a stationary cell is the most commonly deployed approach for studying catalyst dissolution (i.e., does not require a bespoke ICP instrument and setup). The measurement can be performed with a RDE setup or stationary working electrode in an H-cell, where the working and counter compartment are separated by an ion-exchange membrane. During such measurements, an electrochemical method is run (e.g., chronoamperometry hold) and aliquots of electrolyte are collected during the experiment (e.g., before electrochemical testing and at 30 min intervals). In such conventional 3-electrode cells, the dissolved catalyst accumulates in the electrolyte, which is collected as aliquots for ICP measurement. This method provides discrete time-resolved information, and the dissolved species inherently accumulate during the experiment. Zhang et al. measured the concentration of Co and P after immersing Co_2_P catalyst in electrolyte for 5 h and after 2000 linear sweep voltammetry (LSV) cycles between −0.15 V and 0/0.05 V vs RHE.^135^ With an H-cell containing 30 mL 0.5 M H_2_SO_4_ in working compartment, Wang et al. recorded the dissolved concentration of MoS_2_, MoP, and CoP after holding at a designated potential (from 0 to −0.6 V vs RHE, with 0.1 V interval) for 5 min, during which 1 mL electrolyte was collected for ICP-MS measurements.^152^ Interestingly, to show the potential-dependent stability, Wang et al. estimated the HER stability numbers of MoS_2_ (10^5^), MoP (10^4^), and CoP (10^2^). Goryachev et al. evaluated the temporal evolution of CoP_*x*_ dissolution by CA at −0.12 V vs RHE, during which the electrolyte was sampled after 2.5 h, 5.0 and 7.5 h of electrolysis.^153^ Huang et al. monitored the evolution of Co-MoS_2_@CoS_2_ dissolution by sampling the electrolyte at 1, 3, 5 10, 30, 50, 80, 120 h during the CA at −0.1 V vs RHE.^154^ Wang et al. measured the dissolution of Co_0.6_(MnNiZn)_0.4_PS_3_ after a 12 h CP test at 25 and 65 °C, respectively.^155^ Beyond analyzing the concentration of dissolved species in electrolyte, ICP has also been used to determine the residue catalyst loading after HER. For example, King et al. quantified the CoP loading on the Vulcan carbon support and carbon paper GDE, after these electrodes were digested using aqua regia for 24 h.^156^

The measurement of catalyst dissolution by ICP can be influenced by numerous experimental factors (e.g., ionomer content, presence of ion exchange membranes), which are discussed below.

##### 2.3.2.1 Concentration of Dissolved Species

According to the Nernst equation, a higher concentration of dissolved species gives a better stability against subsequent dissolution. Although this has not been directly investigated for HER electrocatalysts, it has been observed in ORR and OER, and thus we postulate the Nernst shift is also applicable to dissolution measurements of HER catalysts.^157−159^ Given that the concentration of a dissolved species is determined by the electrolyte volume, it is clear that the electrochemical cell volume can influence the catalyst dissolution. Indeed, it is possible that different dissolution rates and concentrations may be recorded when measured by the previously mentioned distinct 3 cell designs for ICP (Figure 8). As an example of *in situ* ICP measurement coupled with a flow cell, SFC-ICP-MS is an ideal cell configuration whereby the continuous electrolyte flow mitigates the accumulation of dissolved species.^132,133,141−143^ Therefore, the as-measured result is considered as the closest to the intrinsic stability. To justify the stability estimated by a flow cell, readers are reminded to caution the flow rate/working volume ratio, and the shape of flow channel, which determines the uniformity of flow profile relating to the local saturation. In the case of a stationary nonflow cell (for both *in situ* and *ex situ* measurements), product accumulation is present, which may lead to a better estimation of stability. It is therefore recommended to benchmark results acquired in a stationary cell against those collected in a SFC-ICP-MS configuration to understand how consistent these measurements are and elucidate any deviations. While the measurement in a flow cell is best for an intrinsic stability measurement, it may underestimate dissolution in a real electrolyzer.

##### 2.3.2.2 Ion-Exchange Membrane

The use of ion-exchange membranes between the working and counter electrodes is sometimes deployed to prevent dissolution and redeposition of ions between the two electrodes (discussed in Section 2.2.3.). However, dissolved species can be absorbed and even permeate the membrane. In the cases where membranes are utilized, quantification of the absorbed species in the membrane will be necessary. For example, after immersing a 8 cm^2^ Nafion membrane in a 500 ppb Ir solution (2.6 × 10^–6^ M) for 1 h, ∼8% of the total Ir cations in solution were taken up by the membrane.^160^ Readers are advised to be careful of the potential influence of membrane, especially in long-term measurements, where the membrane has more time to absorb dissolved ions.

##### 2.3.2.3 Ionomer

Nafion or alternative ionomer dispersions are commonly used for preparing powdered catalyst inks which are subsequently deposited onto electrodes to attach powdered catalysts to the electrode surface. The network formed by Nafion macromolecules at least partially covers the catalyst surface. In ORR and OER studies, it has been postulated that this Nafion network can act as a sink and accumulate dissolved species from the electrolyte (e.g., from catalyst dissolution), which can increase the local concentration of such ions and therefore suppresses further catalyst dissolution.^157−159^ With IrO_*x*_ as a model catalyst for OER in 3-electrode cell, increasing the Nafion binder content in the catalyst ink from 5 to 33 wt % gives a 8-fold increase in the measured catalyst stability.^159^ We postulate this ionomer effect might also be present for HER electrocatalysts. Thus, a powder catalyst bound in Nafion could present enhanced stability when compared to the same catalyst composition in the forms of, for example, thin films, which do not involve the use of Nafion binder.

##### 2.3.2.4 Potential Control

As discussed in Section 3.3, transition-metal-based carbides, sulfides and phosphides have shown promising geometric activity in acidic electrolyte, in some cases approaching the performance of commercial Pt/C.^49,156,161^ Alongside these high geometric activities, high stability is often also reported through short CA or CP measurements. As discussed previously, precious metal free HER catalysts often do not undergo dissolution at reducing potentials but are prone to corrosion at OCP upon uncontrolled immersion into the electrolyte. For example, WC, MoS_2_, Ni_5_P_4_, Co_2_P in 0.1 M HClO_4_, measured by an *in situ* ICP flow cell (Figure 8B);^144^ MoS_2_, MoP, and CoP in 0.5 M H_2_SO_4_, measured by an *ex situ* ICP stationary H-cell;^152^ NiMo alloy in 0.1 M KOH, measured by an *in situ* ICP flow cell show such anomalous behaviors.^147^ Although OCP-induced dissolution is a potential barrier to the deployment of precious metal free catalysts in electrolyzer technologies, a protection potential could be applied during start-up/shut-down cycles to avoid catalyst dissolution.

When running an electrochemical measurements, there are two ways to bring the working electrode in contact with electrolyte: 1) the working electrode is immersed into electrolyte solution under OCV (without applying any potential), which is usually accompanied by catalyst dissolution, even for precious-metal-catalyst, such as IrO_*x*_.^159^ 2) the working electrode is immersed into electrolyte under an applied reducing potential, which cathodically protects the catalyst from dissolution at OCV.^162^ Introducing the working electrode into the electrolyte under potential control is therefore an excellent approach to alleviate catalyst dissolution. Although introducing electrodes under potential control might enable the catalyst to appear more stable, we propose that this should be used in addition to measurements that quantify catalyst dissolution when the electrode is held at OCP. This should be reported within the methods section of the paper to ensure transparency. This would enable more precise reporting of catalyst stability as a function of potential.

#### 2.3.3 Post-Mortem Analysis for Monitoring Structural Change

Stability measurements are typically complemented with *ex situ* post-mortem analysis, to probe changes to the catalyst structure, oxidation state, or stoichiometry post electrochemical testing. For example, after 2000 LSV scans with Co_2_P as the HER catalyst in acid, SEM showed no morphology changes; the bulk structure also remained unchanged, as measured by XRD; and XPS revealed the same valence state of Co before and after LSVs.^135^ In contrast, XPS was used to show that on Mo_3_S_13_ clusters, a combination of ∼20 CV cycles and 5 segments of CA measurements increased the valence state of Mo.^146^ XPS on NiMo alloy showed that after 50 CV cycles, Mo was leached out and Ni valence state increased.^147^ In another study, the surface composition of Mo_3_S_13_ clusters remained unchanged after CP, as evidenced by XPS measurement. XPS studies of MoS_2_, Co_2_P, Ni_5_P_4_ and WC revealed that after 4 start–stop CP cycles, the metal to heteroatom ratio of Ni_5_P_4_ remained unchanged, while that of the ratio was altered for the other catalysts.^144^ Similarly, after LSV and CA measurements on CoP*_x_*, AFM showed that the film initially developed a pitted surface, while XPS showed the composition did not change during CA.^163^ In contrast, during LSV most of CoP_*x*_ underwent dissolution. The knowledge acquired from such post-mortem analyses can provide significant information regarding the stability–structure relationships. Of course, operando electrochemical investigations coupled to materials characterization techniques are superior to such *ex situ* characterizations with regards to elucidating such structure–activity relationships. However, these typically require significantly more complex setups and are typically less accessible.

### 2.4 Device Testing

**Figure 9:**
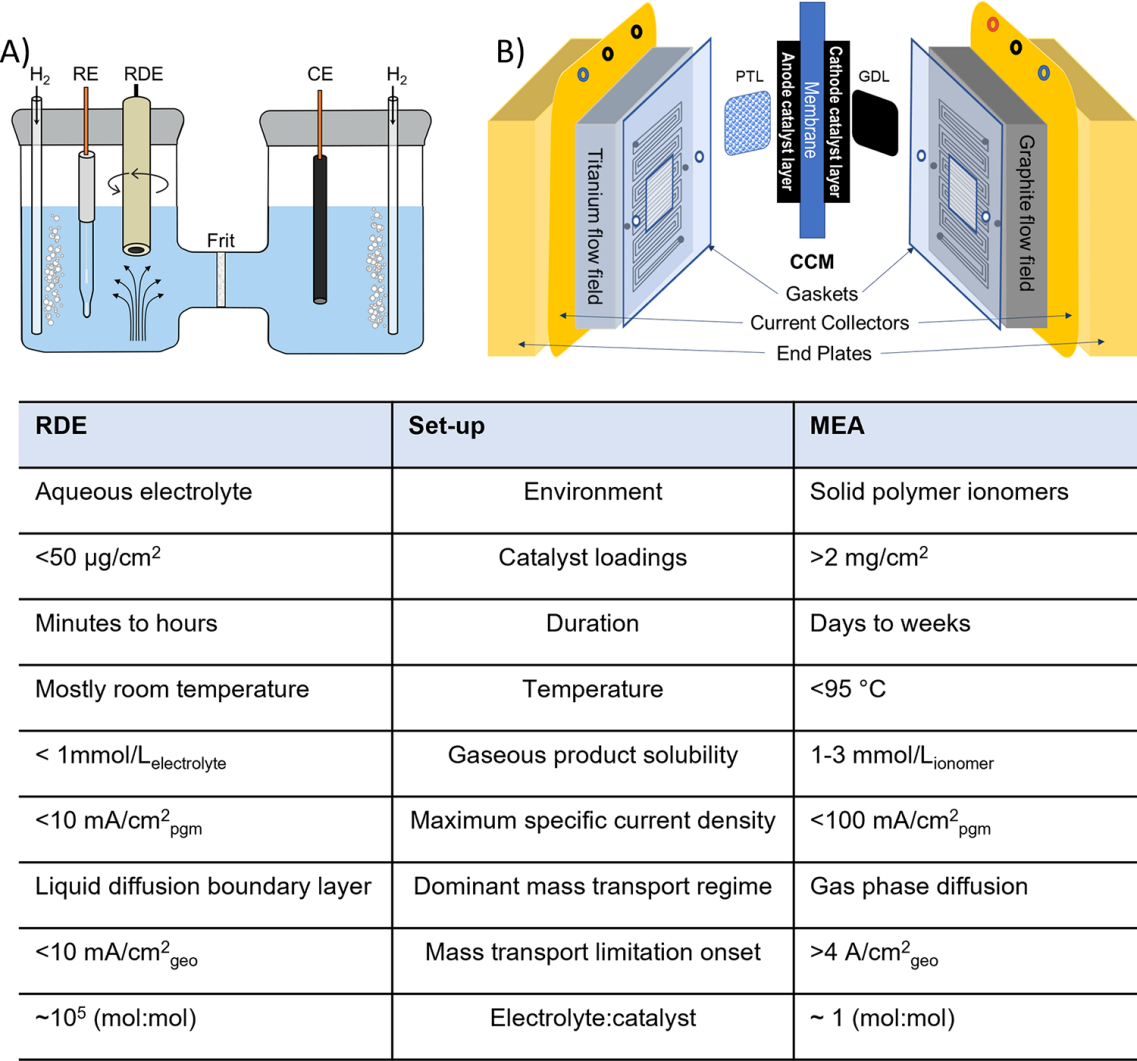
Schematics of H-cell with a rotating disk electrode working electrode (A) and membrane electrode assembly (B). Table to highlight the differences between these two cell geometries given below. The performance and conditions in the table are adapted and reproduced with permissions from ref (164). Copyright 2022 Springer Nature; ref (173). Copyright 2021 Springer Nature.

Translating electrocatalysts characterized in half-cell setups into device (electrolyzer) performances have neither been predictive nor efficient for electrolyzer catalysts (Figure 9) and it is still unusual to see full MEA or AWE testing in the primary literature. Lazaridis et al. discuss the benefits and limitations of RDE testing to conclude that RDE is an important preselecting platform for electrocatalysts, but MEA performance and durability are required to confirm catalyst relevance to commercially relevant devices.^164^ We discuss the recommendations, practices and feasibility of precious metal free catalysts from half-cell to MEA. We also review the benchmarking and best practices for electrolyzer testing from literature. Additionally, in view of the paucity of MEA testing published for precious metal free HER catalysts for PEM electrolyzers, we discuss best practices literature from PEM electrolyzers utilizing Pt and other PGM cathodes where appropriate.

#### 2.4.1 Membrane Electrode Assembly

Du et al. have recently reviewed AEM electrolyzer MEA fabrication, cell assembly and performance evaluation protocols.^11^ Tricker et al. have discussed the design and operating principals of AEM electrolyzers for high performance.^165^ There are also numerous other recent reviews, perspectives and articles that highlight the need to carefully consider choice of component, device and cell fabrication methods, and testing protocols to accurately assess performance and durability of AEM electrolyzer MEAs.^166−170^ In general, to assess the activity, durability and feasibility of PGM free HER catalysts in AEM electrolyzers, all the other components should be maintained identical. Where feasible, commercial half MEAs preintegrated with anode catalyst layers, AEMs and transport layers may be the direct pathway to ensure reproducibility. We acknowledge that finding a commercial MEA manufacturer for AEM is a challenge, and the most feasible route may be to ensure uniform materials and methods within the lab. The commercial half-MEAs can then be completed for electrolyzer tests using the cathode catalysts to be investigated. Once the precious metal free catalyst is assessed for HER performance and durability in half-cell, each catalyst may require optimization toward catalyst ink composition, catalyst layer fabrication methods and MEA pretreatment methods to establish bespoke overall strategy to maximize performance and durability.

As with the half-cell measurements and AEM electrolyzers, the enormous challenge of developing a universally applicable, accurate and reproducible protocol to assess precious metal free HER electrocatalysts in electrolyzer MEAs also permeates to PEM electrolyzers (Figure 9). Analogous to AEM electrolyzers, it is highly recommended to utilize commercially available half-MEAs, whenever feasible, to ensure uniformity and reproducibility. Unlike in the case of AEM, there are commercial suppliers for half and full MEAs for PEM-WEs. The cathode precious metal free catalyst may be integrated either as a catalyst coated membrane (CCM) where catalyst layers are fabricated directly on the membrane or gas diffusion electrode (GDE) where catalyst layers are engineered on the gas diffusion layers (GDLs). CCMs are preferred to GDEs for particulate powder catalysts because the architecture results in improved kinetics.^171^ However, GDEs facilitate the integration of self-supported or catalyst layers directly synthesized on GDLs into the MEA. Additionally, there is evidence that mass transport may be superior in GDE configuration in comparison to CCM, indicating that GDEs may in fact be preferred at high current densities.^172^ GDE fabrications are also easier to handle because the membranes tend to dehydrate and wrinkle during the MEA production, as Holzapfel et al. discuss in reference to fabricating a [Mo_3_S_13_]^2–^ nanoclusters supported on nitrogen-doped carbon nanotubes cathode catalyst layers.^146^

Irrespective of CCM or GDE, the fabrication of the catalyst layer for electrocatalysis should be carefully considered prior to commencing electrochemical measurements. The active area and the actual catalyst loadings should be accurately defined and reported because the activity is most commonly normalized to either area or catalyst mass.^79^ Both quantities can be challenging to report accurately in the case of precious metal free HER catalysts. Catalyst layer fabrication techniques themselves are also vital to achieve optimal kinetics and mass transport. For example, Peng et al. have recently demonstrated that “doctor bladed” catalyst layers in PEM water electrolyzers result in more open and straight pores that result in enhanced mass transport compared to spray coated catalyst layers.^174^ Thus, there is no consensus yet on best practices to assemble electrolyzer MEAs. However, there are some baseline recommendations regarding catalyst loadings, cell break in and testing protocols to improve reliability and reproducibility of performance metrices for PEM-WE, AEM-WE and A-WE as described below.

##### 2.4.1.1 Catalyst Loading

Catalyst loading has direct implications on the device performance in addition to the economic viability of the eventual device. Economic considerations may not be as stringent for precious metal free catalysts as they are for PGM catalysts, but catalyst layer thickness dictates mass transport, cell conductivity and durability of the MEA. Holzapfel et al. established that 3.0 mg cm^–2^ loading was optimal for their molybdenum sulfide cluster based precious metal free catalysts analogous to previous study from Ng et al., also with molybdenum sulfide based precious metal free catalysts.^146,175^ However, optimal catalyst loading (Table 1 and Figure 9) may differ for each precious metal free catalyst as King et al. show extended durability over 1700 h for CoP cathode catalyst layers at only 1.0 mg cm^–2^ loading.^156^

##### 2.4.1.2 Break in and Activity Measurements

For accurate performance analysis and comparison, MEA break in (also referred to as conditioning, incubation, preconditioning, activating and commissioning) should be achieved prior to reporting electrolyzer performance data. We will refer to the process as break in from here on to avoid confusion. Break in can be defined as reaching optimal performance conditions whereby the membrane and catalyst layers are fully hydrated, catalysts have evolved to the optimal and steady state, any detrimental impurities such as impurity cations and anions have been removed and transport of reactants and products have stabilized to a steady state.^176^ Wang et al. have explored the effects of break in on establishing a steady MEA performance by removing MEA impurities, activating the catalyst, optimal mass transport, and fully hydrating the membrane and catalyst layers.^177^ Break in requirements and profiles (voltage and current density cycling and/or holds) for AEM and PEM, and for each precious metal free HER catalyst are likely to be very different. There were significant variations even for relatively mature PEM electrolyzers using commercial PGM catalysts and uniform conditions across five laboratories as reported by Bender et al.^178^ For the study, the laboratories utilized the same commercial catalyst, uniform MEA fabrication methods, cell hardware and testing protocols, and compared the cell potentials required to achieve 1 A cm^–2^. The variations were lower when compared to literature at the time of publication, but the variations between laboratories were 2–3-times higher than within the same laboratory. For precious metal free catalysts and AEM electrolyzers the variations are likely to be significantly higher because the catalysts syntheses themselves have not been optimized unlike the current commercial catalysts, and thus, inherently one needs to compare different types of materials even for the seemingly the same catalyst. However, based on evidence thus far from published literature electrolyzer MEAs should be held at low current densities (about 0.2 A cm^–2^) first followed by high current densities (about 1 A cm^–2^) for 30 min each followed by potential hold at around 1.7 V until the current deviates less than 1% per hour prior to generating the performance polarization curves.^11,177−179^ To generate the polarization curves, voltage should be held for 3–5 min at 20–50 mV steps, and average current density values of the final >30 data points should be plotted against the given voltage.^99^

##### 2.4.1.3 Translation from Half-Cell to Device Testing

As reported in numerous earlier publications and discussed in this review, the overwhelming majority of precious metal free HER catalysts that have been reported in the literature are tested in a half-cell configuration. Indeed, of those tested in acidic electrolyte, only a handful have been integrated into MEAs for electrolyzer testing.^180^ The barriers to translating high performance electrocatalysts from half-cell to MEA (Figure 9) for water electrolyzers are similar to other electrochemical energy conversion technologies such as fuel cells and redox flow batteries. Fan et al. attribute the large discrepancies observed between RDE and MEA measurements to the large differences in current densities, mass transport and catalyst loadings in fuel cell testing.^173^ For the precious metal free HER catalysts, there is similar paucity of translating catalysts from RDE to MEA for PEM, but a substantially higher proportion demonstrated for AEM electrolyzers.

Recent reviews on integration of precious metal free cathode catalysts in electrolyzers show that the CoP cathodes show long-term MEA durabilities comparable to state-of-the-art Pt/C cathodes and highest active area demonstrated to date.^173,181^ Other cobalt catalysts that have been demonstrated in a PEM electrolyzer are cobalt–copper alloy in 1 cm^2^ active area^182^ and Co_3_O_4_ mixed with Vulcan carbon showed superior performance than platinum-black at cell potentials >2.3 V during short-term tests.^183^ King et al. showed the feasibility of replicating the RDE performance of CoP cathodes in an industrially relevant 86 cm^2^ MEA with durability for 1800 h.^156^ Similarly, other phosphides, sulfides, oxides and alloys of molybdenum, nickel, iron, copper and tungsten have also been integrated as PEM electrolyzer cathodes to demonstrate feasibility at single cell level in recent years despite the majority of catalyst research for PEM electrolysis correctly devoted toward lowering or replacing anodic iridium catalysts.^6,175,180,184^ Similarly, there have been single cell level demonstrations using alloys, oxides, phosphides and sulfides of nickel, cerium, lanthanum, molybdenum, iron, copper, and cobalt among others as cathode catalyst layers.^11,12,31,180,185−187^

There are multitude of factors that affect the performance and durability of complex devices like membrane-based electrolyzers (Figure 9). The choice of membrane, active area, gas diffusion layers, porous transport layers, catalyst layer fabrication techniques, catalyst ink formulations, cell structure, cell assembly methods, water pumping orientation (at anode only vs at both cathode and anode) and cell conditioning protocols can all drastically change the performance and durability of MEAs.^11,165,177−179,188,189^ As Bender et al. demonstrated recently there can be variations in reported performances up to three-times higher across different laboratories compared to within the same lab for PEM-WEs (even for the highly optimized commercial components and unified methods).^178^ For precious metal free HER catalyst integrated cathodes, the variations for the beginning of life are likely to be even higher. There are further variations in performance when end of life activities are compared instead of initial performance. A recent review by Tomic et al. compares various durability assessment protocols for PEM-EL reported in the literature, which show wide variety of degradation rates for even commercial catalysts at both the cathode and the anode.^190^

#### 2.4.2 A-WE Device Testing

To demonstrate relevance in the more mature field of diaphragm-based alkaline electrolyzers, one may also adopt various electrochemical methods or protocols pertinent to the harsh conditions in an industrial A-WE electrolyzer. A potential experiment to show industrial relevance could be to test prepared catalyst-coated electrodes for activity and durability under elevated temperatures (e.g., 60–100 °C), higher electrolyte concentrations (e.g., 8 M KOH), and high geometric current densities (e.g., >400 mA cm^–2^).^21^ It is not a common practice to report half-cell studies at elevated temperature for either acidic or alkaline water splitting electrocatalysis. It may be relevant and insightful to explore RDE studies at elevated temperatures to mimic conditions in commercial devices. Such studies may shed light on both performance and durability. Previous studies have shown that both mass transport and kinetics are significantly affected by elevated temperatures and pressures.^191−193^

**Figure 10:**
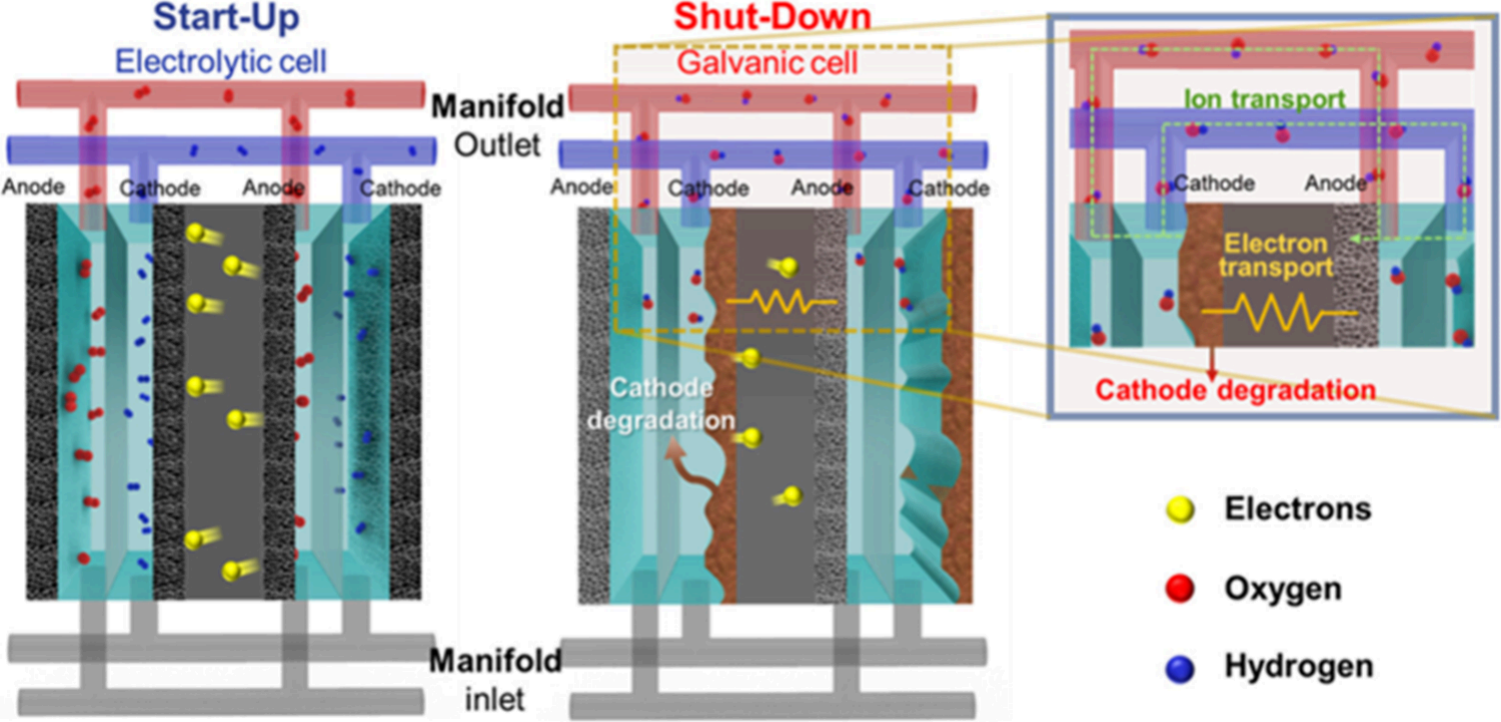
Representation of a bipolar plate electrolyzer during operation (left) and after shutdown (right) showing how reverse currents run, as the bipolar plate and the electrolyte in the manifolds form a galvanic cell that spontaneously discharges. Reproduced with permission from ref (202). Copyright 2022 American Chemical Society.

Beyond temperature and pressure, cell configurations also play critical role in the electrolyzer performance. Innovative laboratory cell setups are required to elucidate effects of various parameters. In a recent study, Leuaa and colleagues introduced an innovative zero-gap alkaline electrolyzer cell.^194^ This cell uniquely allows for simultaneous measurement of the half-cell potentials of both electrodes. They let a strip of the diaphragm protrude from the cell and act as a luggin capillary. This design enables the concurrent investigation of bubble effects on each electrode within the zero-gap configuration, a capability not previously explored in A-WE experiments. With this setup, the researchers identified the nickel-substrate morphology with the lowest *iR* drop at high current densities out of seven different morphologies for both the cathode and the anode. The setup may also help elucidate synergies or antagonisms between cathode and anode pairs. This advancement improves upon traditional two-electrode cell assemblies lacking reference electrodes and may help provide a stepping stone between half-cell measurements and larger-scale electrolyzer testing. However, we must recognize that industrial electrolyzers inherently differ from laboratory setups. Understanding these differences is key to effectively testing the stability and durability of electrocatalysts in real-world applications. A description of the inner workings of an electrolyzer stack (an assembly of electrochemical cells) is therefore necessary. Currently, most alkaline electrolyzers utilize a bipolar arrangement. In such an arrangement, all cathode compartments share a catholyte loop, and all anode compartments share the anolyte loop, a design which minimizes the sum of metal used for the structural parts of an electrolyzer stack. Nonetheless, it introduces an issue related to the ionic and electric connections in the cell. In the bipolar design, the electric current flows through multiple cell compartments linked in series. Adjacent compartments are connected through an electrically conductive plate, connected to an anode on one side and a cathode on the other, giving rise to the name “bipolar plate”. As the bipolar plates form electrical short-circuits and an ionic connection exists via the electrolyte loops, a closed circuit permits spontaneous electrode discharge, dubbed “reverse currents”. This phenomenon is illustrated in Figure 10 and is well-documented in alkaline water electrolysis.^195−200^ Accelerated end-of-life tests that simulate this phenomenon have also been published by Haleem et al. These industrially focused chemical procedures may demonstrate the catalyst’s potential for immediate impact.^201^

As reverse currents are unique to specific stack designs, one may argue that academic research which optimizes electrodes for durability toward this phenomenon is overfocused on existing industrial designs and may be less relevant for future electrolyzer designs. Regardless, accelerated degradation tests can assist in differentiating between stable and unstable regimes, providing crucial insights for intermittently operated water electrolyzers, regardless of the separator composition. Catalysts need to exhibit resilience to off-periods, as intermittent operation of industrial alkaline electrolyzers is necessary for the technology’s economic feasibility in future energy markets where higher fluctuations of energy prices are expected.

### 2.5 Errors and Reproducibility

Across the various different HER activity and stability metrics reported for half-cell and device-scale testing, we note that it is surprisingly uncommon for authors to report their work in replicate. Indeed, studies often report a single series of synthesized catalysts and a single set of experimental results (e.g., a series of LSVs for a series of catalysts with varying compositions). Furthermore, frequently, studies are published with stand-alone electrodes and data within the reported data set. While the costs of assembling device-scale setups might prohibit multiple replicates for some experiments (both time and consumables or components such as membranes, porous transport layers), it is critical that authors consider the uncertainty of their reported findings. At a minimum, as currently reported by some authors, we suggest that all half-cell electrochemical data should be repeated in triplicate and an error bar provided when describing the overpotential, or current density at a given applied potential. Similarly, simple stability tests (e.g., chronoamperometry holds) could also be reported in replicate. While reporting electrochemical data in replicate is a start to understanding the uncertainty of reported data, it is clear that one researcher performing experiments in one laboratory will not mitigate all sources of uncertainty. Indeed, round robin testing, where laboratory intercomparisons are conducted to provide comparisons have shown deviation in PEM-EL testing is 2–3-times higher than the lowest deviation observed at one single laboratory.^178^ While it can certainly be argued that conducting such replicate experiments can only go so far with regards to representing uncertainty, it is also clearly an unfeasible task to ask every research group to undergo such round robin testing. However, such testing should be encouraged for state-of-the-art materials as well as catalysts used for benchmarking. While not exclusive to the HER, a recent publication provides some recommended principles of methodology that relate to 3-electrode electrochemical measurements with application in energy materials characterization that should (1) minimize mistakes and error, (2) maximize reproducibility, and (3) assess measurement uncertainty.^125^

## 3 Precious Metal Free HER Catalysts

### 3.1 HER Electrocatalyst Design Strategies

**Figure 11:**
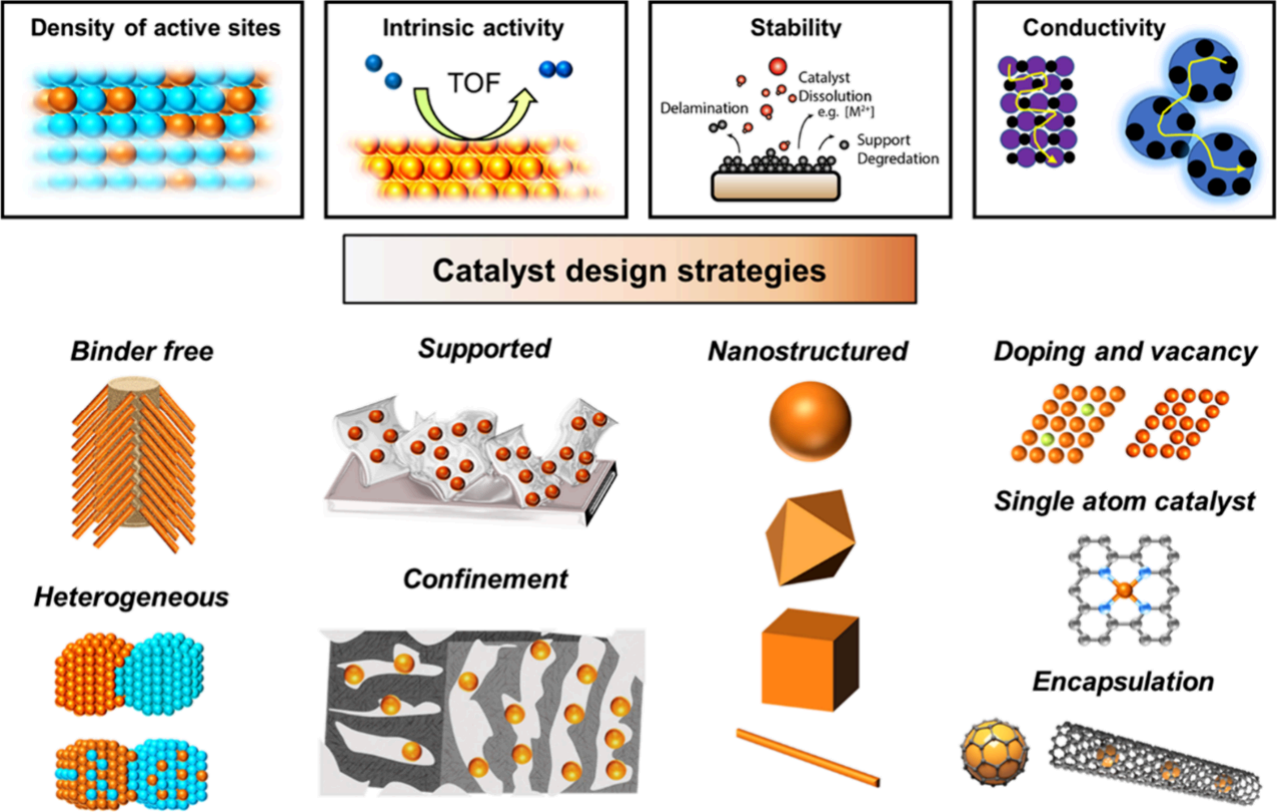
Summary of the various catalyst design strategies pursued toward increasing the density of active sites, increasing the intrinsic activity, improving the stability and enhancing the conductivity of HER catalysts.

In the past decade, a vast and diverse range of precious metal free materials have been designed, synthesized, and screened as electrocatalysts for the HER. In addition to tuning the catalyst performance through changing the catalyst composition, numerous different electrocatalyst morphologies have also been investigated as design strategies for modulating HER performance. Catalyst morphology, structure, and chemical composition each play an essential role in dictating the performance of an HER catalyst. Indeed, such nanoengineering can modulate the electrocatalytic HER performance through: (1) increasing the density of active sites. (2) enhancing the intrinsic activity of a catalyst, (3) improving the conductivity of the catalyst, and (4) increasing the catalyst stability. Each of these parameters is essential for the design of a high-performance HER electrocatalyst and require optimization through careful catalyst design. Typical nanoengineering approaches deployed for tuning the performance of HER catalysts is shown in Figure 11.

To increase the density of active sites, the most common approach is to investigate nanostructuring of the catalyst and electrode, therefore increasing the surface area and the geometric density of active sites. Conversely, enhancing the intrinsic activity of a catalysts is somewhat challenging as it requires detailed understanding of the identity of the catalyst active site and how to manipulate the chemistry or structure to improve the turnover frequency (TOF): for example, considering strategies such as confinement, defect engineering, modulation of active sites, heterostructured catalysts among others. Increasing the electrical conductivity of the catalyst is achieved through various strategies including incorporating conductive carbon support materials, catalyst doping, and other strategies such as binder-free growth of the catalysts directly onto high surface area supports. Finally, literature strategies to improve catalyst stability have included encapsulating catalysts with protective overlayers (e.g., carbon) as well as exploring self-supported catalyst designs.

While developing fundamental insight regarding the intrinsic HER activity and stability is of critical importance to the field, applied work can be equally important to the field, providing design strategies that take into consideration factors such as (1) scalability of the synthesis and (2) how to incorporate catalysts into a working electrolyzer. Critical to precious metal free HER catalyst development is the notion that while low-cost materials have obvious benefits with regards to perceived materials costs, it is still critical to assess the manufacturing required for synthesis of catalysts at scale, as well as manufacturing for incorporating catalysts into the device (e.g., ink formulations, processing conditions and manufacturing methods).

Pertinent to current industrial electrolyzer design strategies is the ability for the HER catalyst to reach and sustain high current densities (>1 A cm^–2^) for extended periods of time. Such high current densities therefore require high intrinsic electrocatalyst activity, but also demand the incorporation of electrocatalysts (electrode assemblies) that enable high mass transportation (removal of gas bubbles which otherwise block the electro-catalyst active site).^117,203^ In particular it is noteworthy that owing to the lower intrinsic activity of precious metal free HER catalysts, higher catalyst mass loadings are required to reach >1 A cm^–2^ compared to PGM-based catalysts. At such high catalyst loadings, ohmic resistances and mass transport can become limiting. Translating catalysts into electrolyzer technologies is an arduous task by nature as discussed in Section 2.4.1.3.

In this section we provide a succinct overview of some of the common approaches used in precious metal free catalyst design focusing on various nanostructured catalyst designs.

#### 3.1.1 Nanostructured HER Catalysts

The most frequently deployed design strategy for increased precious metal free cathode performance is catalyst nanostructuring. Through an expansive and diverse range of synthetic methodologies, a vast and diverse library of shapes, sizes and morphologies have been explored as catalysts for the HER. Various reviews have been published on nanostructuring HER catalysts, including reviews focused on nanostructured phosphides,^204^ nitrides,^205^ nickel-based electrocatalysts,^206,207^ molybdenum disulfide,^208^ and core–shell morphologies.^209^ More broad reviews on nanostructured HER catalysts have also been published.^210−212^ At the crux of nanostructuring catalysts is the notion that by increasing the catalyst surface area, the geometric density of active sites will also be increased. Accordingly, a seemingly higher electrode “activity” can be achieved when the current is normalized to the geometric surface area without increasing the catalyst loading. Thus, the “atomic economy” of the electrocatalyst is increased.

Beyond increasing the geometric density of active sites, nanostructuring catalysts can potentially impact the catalyst intrinsic activity as well as the catalyst stability. For example, through design of cubes or spherical particles, different crystal facets could be selectively exposed altering the catalyst intrinsic activity and/or stability. Comparisons between catalysts compositions with differing nanostructures therefore requires careful benchmarking and protocols that ensure fundamental understanding of any changes to catalyst intrinsic or extrinsic activity is reported appropriately (e.g., normalized to catalyst surface area). Section 3.3.1 discusses the various different nanostructures explored for MoS_2_-based catalysts. See Section 2.2 for discussions pertaining to such best practices.^85^

#### 3.1.2 Binder-Free, Self-Supported Electrocatalysts

It is the overall electrolyzer design (specifically the electrode manufacturing) that dictates how the HER electrocatalyst is best incorporated into the device. For example, for a typical MEA-based electrolyzers (PEM or AEM), the catalysts are synthesized as powders (nano- and micro- particles) which are subsequently formulated into an ink composed of the catalyst, a solvent (e.g., water and/or IPA) and binder (e.g., Nafion). The catalyst ink is subsequently deposited onto either the membrane or GDL, and finally assembled as a cathode for the MEA. Accordingly, for PEM water electrolyzers, catalysts are typically prepared as powders, enabling scaleup of the catalyst synthesis independent of a conductive substrate. Conversely, the binder-free electrode design, whereby the catalyst (or catalyst precursor) is synthesized directly onto conductive supports is commonly utilized in liquid A-WE. Binder-free electrodes mitigate the use of conductive additives and binders and therefore significantly minimize the number of processing steps and complexity required for assembling the electrode. Owing to this physical adhesion between the catalyst and substrate, it is also claimed that this design strategy may result in low interfacial resistances (high electrical conductivity at the substrate-catalysts and catalyst-electrolyte).^118,213^ Additionally, the anchored nature of the catalysts is thought to mitigate catalyst agglomeration and delamination under operation, which might enhance catalyst stability. This strategy is commonly deployed for commercial AWE cathodes. It is important to note that although beyond the scope of this review, binder-free HER catalysts designs have also gained significant attention for deployment in photoelectrochemical water splitting whereby direct contact between the semiconductor and catalysts is often required and requires careful design strategies to minimize photon absorbance in the catalyst layer (shadowing) resulting in a reduced current density.

It is critical to emphasize that binder-free self-supported electrocatalysts can also provide an important platform for fundamental HER research. For example, the study of catalysts prepared as thin films on flat substrates which offers a useful synthetic approach to preparing catalysts with well-defined surface areas.^64,69,214^

High surface area substrates are typically deployed for binder-free HER cathodes. Critically, substrates must be able to withstand the same electrochemical potentials and pH experienced by the catalyst (i.e., acidic for PEM or alkaline for AWE). Thus, typically, carbon-based substrates are deployed for PEM, whereas a wide range of materials (e.g., nickel, carbon, stainless steel, aluminum) have been explored for alkaline-based technologies. Binder-free, self-supported electrocatalysts have been reviewed elsewhere,^215−217^ for example, high surface area metallic 3D foams and meshes (e.g., nickel foams^218−220^), and cloths (carbon^221−224^). Planar metallic foils (e.g., stainless steel, titanium, aluminum),^69,95,225^ silicon wafers,^214^ have also been explored for the preparation of binder-free, self-supported catalysts.

#### 3.1.3 Catalyst Supports

There are many reasons why catalyst–supports are of interest to designing high performance and commercially relevant HER active catalyst motifs.^226^ Most commonly, an electrocatalyst support provides a conductive high surface area substrate for the attachment of a nanostructured catalyst.^227^ Thus, one of the primary reasons for utilizing a catalyst support is that similarly to the binder-free catalyst design, the support can provide a high surface area scaffold for achieving a high dispersion (scaffolding) of the catalysts across a cheap or abundant support. Indeed, commercial Pt-based HER catalysts are dispersed on high surface area carbon supports in PEM-WEs. Supported catalysts can also (in some cases) be easier to disperse, and therefore can improve ink formulations and the fabrication of MEAs. This is critical for the preparation of catalyst layers whereby porosity and conductivity networks are essential for tuning the mass transport and overall device performance.

The electrical conductivity is an essential parameter in catalyst layers, with significant impact on the overall operation of an electrolyzer. Specifically, there must be a conductive pathway to facilitate the delivery of electrons to the catalytic active site from the current collector plates in the electrolyzer. Conductive catalyst supports can therefore result in enhanced (or diminished) charge transfer from the catalyst to electrode. For the deployment of catalyst–supports, the conductivity of the support material must therefore be considered. Indeed, the moiety must be sufficiently conductive such that when it is integrated into a PEM-WE catalysts layer the *iR* drop does not significantly impede the device performance. Owing to their high stability and high conductivity under HER relevant potentials and conditions (e.g., acidic media) as well as being cost-effective, carbon materials are commonly deployed as catalyst–supports.^228^ Indeed, various forms of carbon (e.g., carbon nanotubes, graphene, fullerenes, reduced graphene oxide) as well as doped-carbon structures (e.g., N,^229−233^ P,^234−236^ or B-doped) are commonly deployed. Relatively few noncarbon supports have been explored as catalyst supports and the majority of these studies are for Pt-based catalysts.^228^

Electronic interactions between the catalyst and support can also be used to engineer or tune catalytic activity toward the HER, for example, transferring charge from the substrate to the catalyst and potentially tuning reaction intermediate adsorption energies.^226^ Tuning binding energies (Δ*G*_H*_) can result in improved catalytic activity for the same catalyst but dispersed on a different support. This has been shown as a promising route to enhancing the intrinsic activity of some HER catalyst. It is important to note that the catalyst–support electronic influence is only present within the first few nanometers of the catalyst. Thus, catalyst–support designs to exploit this effect require careful design and synthetic considerations. Such studies have included purely theoretical studies, as well as investigates that combine theory and experiment. Purely theoretical studies have largely focused on the role of catalyst supports on MoS_2._ For example, it was demonstrated that various single crystal facets (Ir(111), Pd(111), Ru(111)) are able to shift the hydrogen binding energy monolayer MoS_2_ by up to 0.4 eV.^237^ In subsequent studies, MoS_2_ overlayers on Au (111) and graphene were also conducted, highlighting the study of catalyst–support interactions as an important design strategy for tuning HER catalyst performance.^238^ Interfacial sites between Ni_3_N and a Ni support were also shown to have near optimal hydrogen binding energies when compared to Ni and Ni_3_N in isolation by DFT calculations.^239^ This phenomena was used to explain the high HER performance of these very high surface area Ni_3_N/Ni (12 mV overpotential required to reach 10 mA cm^–2^ in 1.0 M KOH). In acidic media, several combined experimental and theoretical investigations have shown the role of catalyst support on HER activity of transitional metal dichalcogenides. For example, the thiomolybdate nanocluster [Mo_3_S_13_]^2–^, which contains a structure that resembles the active edge site of MoS_2_, was deposited on various substrates (gold, silver, glassy carbon and copper rotating disk electrodes).^240^ A clear difference in the electrochemical overpotential and TOF was observed as a function of substrate, which through DFT calculations was shown to follow a volcano-type trend which is qualitatively similar to previously reported HER volcano plots. In a similar example, 2D TaS_2_ was grown by CVD on four different substrates (glassy carbon, carbon fibers, Mo and Au foils).^241^ The highest performance catalyst-substrate was found to be TaS_2_ on Au foil (101 mV to achieve 10 mA cm^–2^). Accredited to suitable lattice mismatch and charge injection between TaS_2_ and Au, an enhanced HER activity was reported when compared to the alternative substrates. Similar effects have been reported in relatively few nanostructure heterostructures, for example a W_*x*_C@WS_2_ catalysts.^242^

#### 3.1.4 Spatial Confinement

Catalysis confinement deploys constrictions to confine the catalyst activity within a microenvironment.^243,244^ Through careful synthetic design, the shape, size, and hydrophobicity of a catalyst can result in a distinct environment surrounding the catalyst active sites which can therefore tune the catalyst performance (e.g., activity or selectivity).^244^ The impact of confinement can indeed be either positive (improve the performance) or negative (reduce the performance). Confinement is broadly analogous to the structure of an enzyme whereby the active site is commonly found at the end of a channel and therefore provides a distinct local environment compared to the bulk. Accordingly, the geometric and electronic structure of the catalyst can be used to control catalyst functionality through control of reaction intermediate adsorption energetics (potentially providing additional binding sites that can tether intermediates, promoters or ligands in place during electrocatalysis at a nearby active site). Additionally, a confined microenvironment highly restricts mass transport, which can limit the diffusion of reagents, intermediates, or products to or from the catalytic active site as well as controlling the local electrolyte environment (e.g., pH, counterions). Finally, catalyst stability could potentially be enhanced through the confinement, slowing the diffusion of dissolved material, or preventing agglomeration.

Very few studies have explored confinement effects for the HER. However, one study investigated Co(OH)_2_ nanoparticles confined within few-layer MoS_2_ nanosheets as HER catalysts under alkaline conditions.^245^ The study showed that in combination, the confined Co(OH)_2_ nanoparticles enables both an acceleration of water dissociation kinetics (enhancing the HER kinetics) while also improving the nanoparticle stability (e.g., preventing agglomeration).

While limited work has explored confinement as a pathway to boosting HER activity, confinement has gained significantly more attention for more complex electrochemical reactions such as carbon dioxide reduction (CO_2_RR) and nitrogen reduction (N_2_RR) where hydrogen evolution is often a parasitic side reaction that consumes electrons and therefore leads to low catalytic selectivity. In one such study, designed to probe the role that mass transport can play in tuning CO_2_RR Faradaic efficiencies, a rotating ring disk electrode study utilizing a gold working electrode disk was used to monitor the Faradaic efficiency for CO formation under alkaline conditions as a function of rotation rates (i.e., under various mass transport conditions).^246^ It was shown that as the mass transport increased, removing the supply of OH- from the electrode surface and therefore decreasing the local alkalinity, resulting in a decrease in HER activity, favoring CO_2_ reduction to CO. In another study, well-defined mesostructured silver inverse opal electrodes were prepared.^247^ In 0.1 M KHCO_3_ electrolyte, the electrode structure mesostructure was found to significantly decrease the HER specific activity (10-fold) while dramatically increasing the specific activity for CO_2_ reduction to CO. Similarly, Ag@C catalysts were prepared whereby catalytically active silver resides on the inside of hollow carbon spheres.^248^ These electrocatalysts were reported to suppress the HER in acidic electrolyte owing to the confined local alkaline environment within the hollow catalyst structures under reaction conditions. In a slightly different approach, highly ordered 2D nanosheet lamella assemblies with controllable interlayer spacings (0.9–3.0 nm) were prepared. Specifically, thin titania nanosheets were assembled with adjustable interlayers. SnO_2_ nanoparticles were incorporated within the interlayer spacing. Similarly to previous studies, a strong dependence on interlayer spacing was observed, with an optimal spacing of 2.0 nm found to reduce the HER, reaching a CO_2_RR selectivity of 73% to formate in 0.1 M KHCO_3_.^249^ Similar enhancements have been observed through computational studies using quantum mechanics.^250^ Interestingly, this study suggested that space confinement stabilized the key CO_2_RR intermediates rather than impacting the key HER intermediate, *H.

In summary, confinement is commonly deployed as a design strategy to alter the selectivity of a catalyst. Interestingly, confinement has been most widely deployed for more complex electrochemical reactions (e.g., CO_2_RR) where HER represents a parasitic competitive reaction. These studies frequently report that confinement can suppress the HER.

#### 3.1.5 Encapsulated Catalysts

Ultrathin shells or overlayers encapsulating catalytically active nanoparticles have been explored as a design strategy for HER electrocatalysts. Closely related to the confined catalyst design strategy, here we distinguish the two based on whether the encapsulated material is in direct contact with electrolyte (confinement) or not (encapsulated). For both acidic and alkaline HER, encapsulated catalysts have been touted as a promising strategy to enhance the long-term durability of active electrocatalysts, typically exploiting carbon-based overlayers (doped or undoped) over transition metal nanostructures. Thus, the overlayers are considered physical barriers to prevent catalyst dissolution and agglomeration.^117,251^ Beyond stability, motivation for highly conductive overlayers (e.g., carbon-based catalyst encapsulation) has included improved catalyst electrical conductivity,^252,253^ as well as a route to regulating the hydrogen absorption and desorption and therefore improving the catalytic intrinsic activity.^254,255^ Confinement engineering of electrocatalysts has been reviewed elsewhere.^243,244,256^

Ultrathin metal oxide overlayers (e.g., SiO_*x*_) have also been explored as overlayers for Pt-based HER catalysts,^257^ however, the overwhelming majority of precious metal free encapsulated catalysts deploy carbon based overlayers. Various different carbon-based overlayers have been investigated for precious metal free HER catalysts, from single layer graphene overlayers,^258^ few layer graphitic structures, to encapsulation within carbon nanotubes.^259,260^ Typically, the thickness of the overlayer is critical, with the near single-layered carbon encapsulation typically showing the highest HER activity. Beyond pure carbon coated catalysts, doped (N, P, B) carbon structures have also shown promising strategies for stabilizing precious metal free HER catalysts. Such dopants have been accredited with the benefit of tuning intermediate binding energies for improved catalytic activity, as well as a pathway to further increasing catalyst conductivity. Numerous different nanostructured precious metal free materials have been explored as the core for such encapsulated catalyst design. For example, various different metallic (e.g., Co,^258,260^ Ni,^261^ Fe^262^), alloyed (e.g., NiCu,^263^ nickel FeCo, FeNi, CoNi, FeCoNi^258^), oxides (e.g., cobalt oxide^264^), phosphides (e.g., FeP,^235^ CoP,^265^ Co_2_P-CoN,^259^ Cu_*x*_Co_3–*x*_P,^266^ Cu_3_P,^236^ MoP^252^), carbide (e.g., WC,^267^ Mo_2_C^268^) nanoparticles have been prepared encapsulated with carbon overlayers for the HER.^256^ Catalyst encapsulation has been reviewed extensively elsewhere giving considerable details regarding the various catalysts and coatings that have been investigated.^256,269^

Pyrolysis of metal organic frameworks (MOFs), including zeolitic imidazolate frameworks (ZIF) has gained significant attention as a route to fabricating electrocatalysts for the HER and been reviewed elsewhere.^270−272^ In brief, upon pyrolysis, metal nodes within the MOF structure agglomerate to produce nanostructures (e.g., nanoparticles) which under some conditions become encapsulated within a carbon-based layer which originates from the organic-linkers within the MOF.

Encapsulation has certainly proven a popular approach. While the performance of these catalysts has indeed shown promise under certain testing conditions, it is important to acknowledge that synthetically it would be extremely challenging for the majority of these studies to prepare careful control samples with the same catalyst loading, composition and morphology without the encapsulating shell to convincingly establish the effect of encapsulation.

#### 3.1.6 Single Atom Catalysts (SAC)

**Figure 12:**
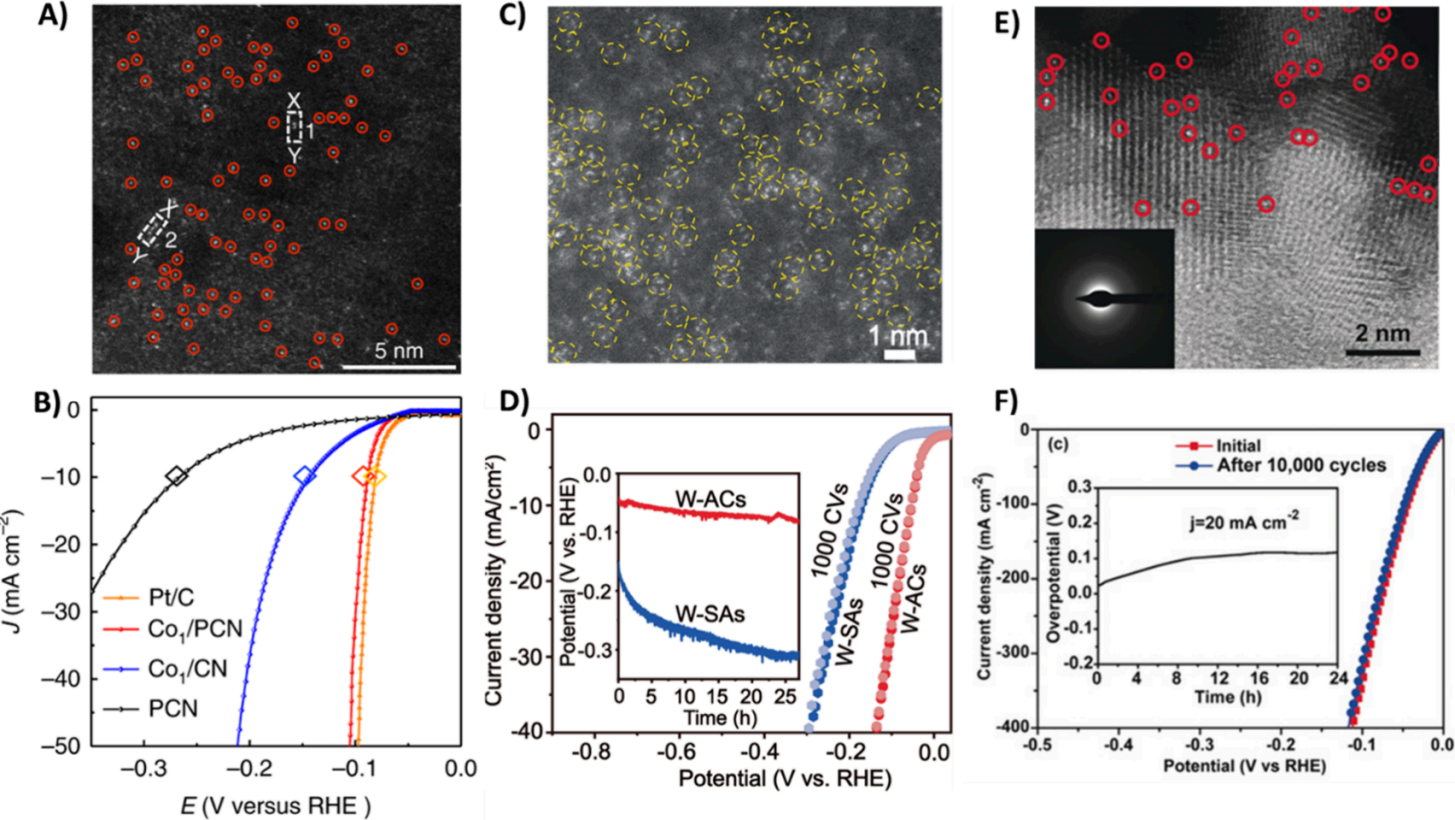
Examples of single atomic and atomic cluster catalysts for the HER. Single atomic Co supported on a phosphorized carbon nitride (PCN) electrocatalyst: (A) atomic resolution HAADF-STEM of Co/PCN (single Co atoms marked with a red circle), (B) electrochemical HER performance of Co/PCN and Co/CN single atomic catalysts. W-based atomic clusters (W-AC) and W single atomic catalysts (W-SAs), (C) STEM image of W-ACs with W–W structures indicated by yellow dashed circles, (D) linear sweep voltammetry of W-SAs and W-ACs before and after 1000 CVs with chronopotentiometry measured at current density of 10 mA cm^–2^ (inset). Single-atom nickel iodine (SANi-I) HER electrocatalysts, (E) HAADF-STEM image post 96 h of stability testing, (F) linear sweep voltammetry of SANi-I before and after 10,000 cycles with chronoamperometry measurement at 20 mA cm^–2^ (inset). All 3 HER catalysts were assessed in 1.0 M KOH. Reproduced with permissions from ref (288). Copyright 2022 Springer Nature; ref (289). Copyright 2019 Wiley-VCH; ref (286). Copyright 2019 Springer Nature.

Isolated single atom catalysts (SACs) have recently gained significant attention as heterogeneous catalysts.^273,274^ By design, SACs offer a catalyst design that improves atom utilization beyond that achievable through nanostructuring. However, conversely to most nanostructures, SACs must be anchored to a support and therefore, their host material influences the catalyst local environment, stability, and electronic properties (e.g., orbitals, electron density). SACs for the HER have been reviewed elsewhere.^275−277^ It is important to note that the majority of SAC studies for the HER deploy Pt or Ru single atoms and are therefore not within scope of this review. Examples of precious metal free based SAC catalysts for the HER include Fe,^278^ Ni,^278−281^ W,^233^ Co,^279,282−286^ and V^287^ SACs. It is particularly notable, that a significant number of SAC studies for the HER deploy Co. For example, a single atomic Co site catalyst dispersed on a phosphorized carbon nitride support (Figure 12A and B), prepared by an incipient wetness impregnation, was able to reach a TOF of 0.22 s^–1^ at 50 mV overpotential under alkaline conditions.^286^ Similarly, a MOF derived Co single site atom costabilized by P and N atoms under acidic media demonstrated 1.6 s^–1^ at overpotentials of 100 mV.^284^ Co-based SACs have also been prepared on graphitic carbon nitride combined with reduced graphene oxide supports.^283^ The obtained catalyst achieved a TOF of 10 s^–1^ at 450 mV overpotential. To probe the origin of Co SAC HER activity, a combined experiment and theory approach was utilized.^282^ DFT calculations were thus used to identify the active site motif, suggesting that the highest HER activity originated from edge modes (compared to plane models). Experimentally, methods were subsequently designed to increase the presence of such edge sites, which was shown to increase the HER performance, reaching TOF values of 0.1 s^–1^ at 100 mV overpotential.

The vast majority of HER SACs are anchored to carbon-based supports (e.g., graphene, graphite, nitrogen-doped carbons, carbon nanotubes). However, HER active MoS_2_ has also been explored as a support for HER Ni-based SACs in acidic electrolyte, with a 1.16 s^–1^ TOF at 40 mV overpotential and requiring only 85 mV to achieve 10 mA cm^–2^.^281^ Similarly, single-site vanadium substitutions into 1T-WS_2_ monolayers have achieve TOFs of 3.01 s^–1^ at 100 mV, requiring 185 mV to achieve 10 mA cm^–2^.^287^ Two-dimensional molybdenum carbide (MXene) has also demonstrated promising activity when substituting single Mo metal atoms with Co, achieving a TOF of 0.1 s^–1^ at 250 mV overpotential.^285^ While the overwhelming majority of SAC literature focused on metallic SACs, single iodine atomic catalysts dispersed on a nickel foam (Figure 12E and F) have also been synthesized and characterized for the HER, achieving 10 mA cm^–2^ at 60 mV overpotential.^289^

Beyond single-atom catalysts, atomic clusters and dual atomic structure have also been explored for the HER. For example, W-based atomic clusters (anchored on P-doped carbon materials, Figure 12C and D),^288^ and dual atom NiFe (supported on carbon nanotubes),^280^ and O-coordinated WMo heterodimers (embedded onto N-doped graphene)^290^ catalysts have been shown to be active for the HER.

#### 3.1.7 Heterostructured Catalysts

A wide range of heterostructured catalysts have been investigated for the HER. These multicomponent hybrids or heterostructures are composed of multiple compounds or crystalline phases in close contact. Such designs can enable enhanced catalytic performance through improvements to more than one parameter that contribute to the overall catalyst performance. For example, heterostructures can improve adsorption energetics of reaction intermediates while also improving electronic conductivity, stability or other such parameters in the catalyst performance. An alternative approach is whereby the close proximity of the two components provide a unique interaction (such as a catalyst–support interaction that tunes the adsorption energetics of the HER intermediates). Heterostructures strategies can therefore incorporate or assemble a diverse range of nanostructures or components.

In one example of heterointerfaced catalysts, a Ni_2_P-NiP_2_ catalyst structure was synthesized and examined for alkaline HER.^291^ Using a combined experimental and theoretical approach, DFT calculations indicated that the metallic-metalloid composite results in electronic transfer that tunes the adsorption energy of H as well as potentially enhancing electronic conductivity in the sample. In a similar strategy, Ni_2_P/Ni@C with the metallic Ni core was also investigated and shown to enhance the HER activity.^292^ Various other heterostructured catalysts such as NiS/Ni_2_P/carbon cloth,^224^ Co@CoFe-P nanoboxes,^293^ branched CoSe_2_@CoNi,^294^ NiCo nitride/NiCo_2_O_4_/graphite fibers^295^ and vertically aligned oxygenated CoS_2_–MoS_2_ heteronanosheets^296^ have also been explored toward enhanced catalytic activity. Beyond improved activities, lattice matching between two constituent electrocatalysts (iron, nickel and cobalt phosphide with molybdenum carbide) was found to correlate with the stability of the catalysts against dissolution in acidic electrolyte.^297^

#### 3.1.8 Defects: Doping and Vacancies

The introduction of defects in a catalyst through the insertion or substitution of a small fraction of “host atoms” with “dopant” atoms or “vacancies” disrupts the chemical bonding across the overall material and can thus have a significant impact on catalyst performance. Clearly, the dopant identity (atomic structure, size, electronegativity) alongside the host chemistry will result in modulation of the electronic structure of the catalyst. As a consequence of defect engineering, the electrocatalytic intrinsic activity can be increased if (1) the defect itself, or nearby sites that are altered as a consequence of the defect are the active site for the HER, or, (2) doping introduces an additional (second) active site which might result in a benefit owing to a tandem catalyst mechanism. Beyond altering the HER activity, defect engineering can also alter the conductivity of the catalyst through enhanced charge transfer to the catalyst active site, thereby benefiting catalyst electrode performance.

Various metallic dopants have been explored for the HER. For example, M-doped (M = Ni, Mn, Fe) CoP hollow polyhedron frames,^298^ Cu-doped CoP on nitrogen-doped carbon,^266^ Co doping into a host MXene matrix (Mo_2_CT_*x*_:Co),^285^ V-doped Ni_2_P,^222^ V-doped CoP_2_^299^ and CoP,^300^ S-doped NiFeP.^223^ In a systematic approach, DFT was combined with experiments to probe Ti, Cr, and Fe-doped nickel films for the HER and it was shown that the dopants modulate the binding energy of hydrogen and hydroxyl ions.^69^ Cr-incorporated Ni was found to optimize these binding energies for the HER. Examples of various nonmetallic dopants deployed to improve HER activity include; S-doped NiFe_2_O_4_/NF,^301^ N-doped NiMoO_4_ nanowires,^302^ N-doped CoP grown on a conductive carbon cloth,^303^ N-doped Co_2_P nanorods grown on a carbon cloth,^304^ P-doped Mo_2_C nanoparticles on carbon nanowires,^305^ and Β-Mo_2_C spheres with Co-doping and Mo-vacancies,^306^ Similarly, defect engineering such as surface S vacancies in 2H-phase MoS_2_ has shown to have a significant impact on catalyst performance.^307,308^ Beyond doping the catalyst, doping of carbon deployed in the catalyst structure, has also been explored, for example, N, P codoped carbon coating transition metal phosphides,^235^ Co_2_P embedded within a N, P codoped carbon,^234^ N-doped carbon-dots loaded with MoP nanoparticles,^231^ Fe–Co-oxide/Co-metal@N-doped carbon on Ni foam,^309^ CoP-nitrogen-doped carbon@NiFeP nanoflakes,^232^ P-doped g-C_3_N_4_ as a support for Co SAC.^286^

### 3.2 Representative Precious Metal Free HER Electrocatalysts

**Figure 13:**
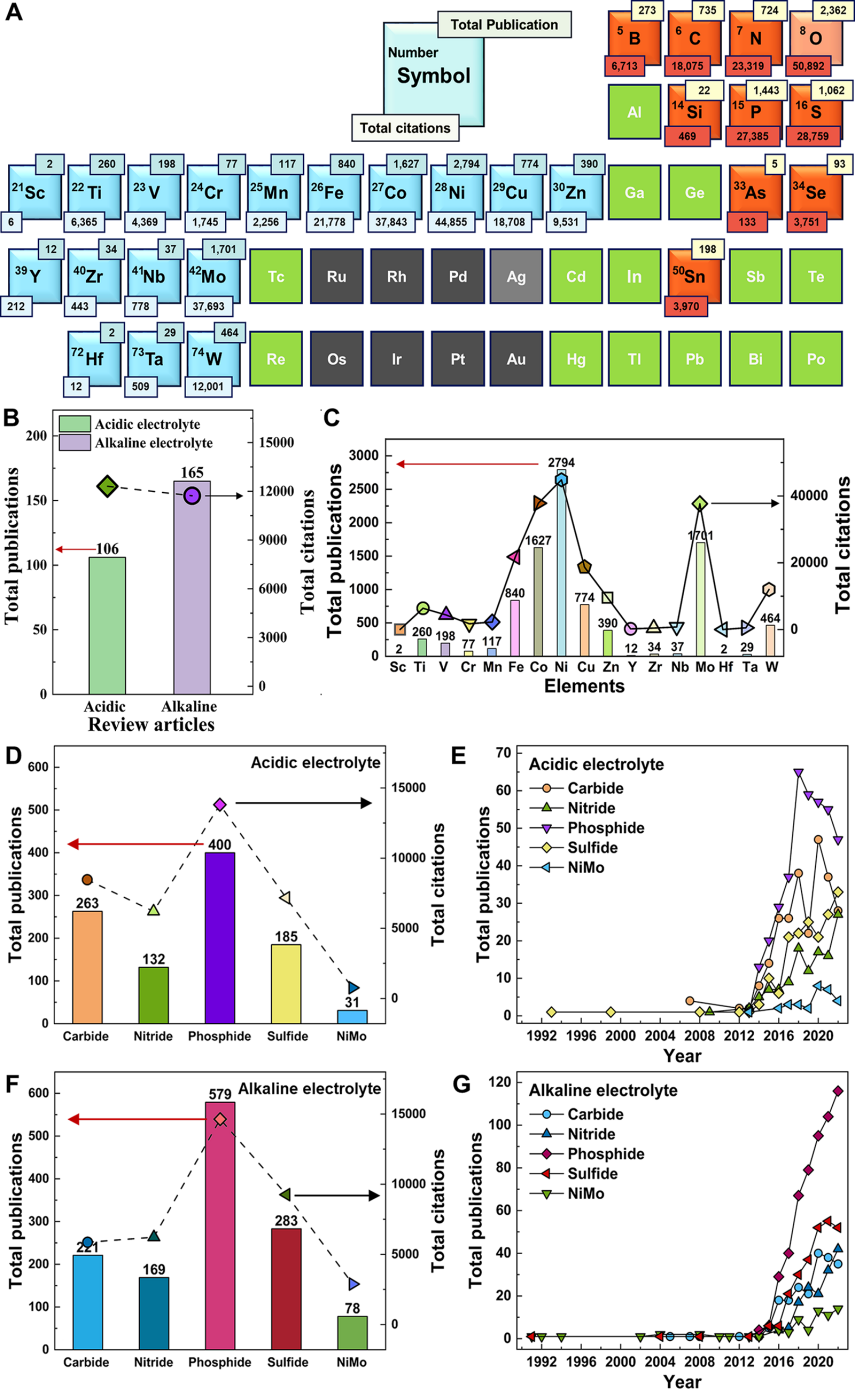
(A) Schematic in the shape of a fragment of the Periodic Table showing the total citations and publications (Web of Science) of the various commonly deployed nonprecious metal elements. (B) Total number of review articles (published and citations), on the topic of acidic and alkaline hydrogen evolution. Total publications and citations for HER catalysts containing (C) various *d*-block elements, (D) common families of materials studied for the HER under acidic electrolyte, and (F) common families of materials studied for the HER under alkaline electrolyte. Chronological trend for carbide, nitride, phosphide, sulfide and NiMo measured in (E) acidic electrolyte and (G) alkaline electrolyte. For the search of metal elements in Web of Science, (TS = (“hydrogen evolution reaction”)) AND (AB = (specified metal e.g., “cobalt”)) NOT (DT = (review)) is used. For the review articles, (TS = (“hydrogen evolution reaction”)) AND (AB = (review)) was used. For review articles regarding either acidic or alkaline/basic electrolytic, (TS = (“hydrogen evolution reaction”)) AND (AB = (review)) AND (AB = (ACID*)) and (TS = (“hydrogen evolution reaction”)) AND (AB = (review)) AND (AB = (Basic) OR AB = (alkaline)) were used, respectively. For the search of carbide, nitride, oxide, phosphide, sulfide, arsenide, selenium and tin, the input contexts used (TS = (“hydrogen evolution reaction”)) AND (AB = (specified catalyst, e.g., “Tin”)) AND (AB = (ACID*)) NOT (DT = (review)) for acidic electrolytes, while the alkaline electrolytes adopted (TS = (“hydrogen evolution reaction”)) AND (AB = (specified catalyst, e.g., “Tin”)) AND (AB = (basic) OR AB = (alkaline)) NOT (DT = (review)). All searches were completed in June 2023.

The majority of non-precious metals in the p- and d-blocks of the periodic table have (in some way) been explored as an electrocatalysts for the HER. This is illustrated clearly in Figure 13A which shows the total number of publications and total citations that include “hydrogen evolution reaction” and that particular element (e.g., “cobalt”) in the title, abstract and/or keywords of primary literature. As an example, in the search for “cobalt” containing electrocatalysts a Web of Science search was conducted, where the input context of (TS = (“hydrogen evolution reaction”)) AND (AB = (cobalt)) NOT (DT = (review)) was used. While the search of their HER in using acidic electrolyte or alkaline electrolyte, the input languages were (TS = (“hydrogen evolution reaction”)) AND (AB = (cobalt)) AND (AB = (acid*)) NOT (DT = (review)) or (TS = (“hydrogen evolution reaction”)) AND (AB = (cobalt)) AND (AB = (basic) OR AB = (alkaline)) NOT (DT = (review)). At the time of collection, there were a total of 51,586 publications published on the “hydrogen evolution reaction” (searched on 23rd August 2023). Reflective of this high number of primary literature publications, it is also notable that the number of review papers published in some capacity on the HER is extremely high (in total 271, Figure 13B). The number of citations for acid vs alkaline electrolyte suggest that non precious metal HER investigations in both pH regions receive a similar level of attention within the academic literature. To understand the most frequently deployed precious metal free, we display the total number of publications and citations for the nonprecious metal elements across the d-block and p-block of the periodic table. Furthermore, we plot the total publications for the transition metals in Figure 13C. Evidently, nickel, molybdenum, cobalt and iron are the most popular metals deployed in precious metal free electrocatalysts.

To assess which are the most commonly explored materials or families of materials within the two distinct electrolyte environments we also show the number of publications reported for 5 families of materials: carbide, nitride, phosphide, sulfide and NiMo (Figure 13D and F). Interestingly, under both acidic and alkaline conditions, phosphides, carbides and sulfides have (by this metric) received the most attention in the academic literature. In both electrolytes phosphides appear to be the most extensively studied in the academic literature. Finally, we track the number of publications for each of these families of materials (acid and alkaline, Figure 13E and G, respectively) each year. Again, it is clear that whereas research efforts in acidic electrolyte appear to have slowed, research focused on HER catalyst development in alkaline solution is continuing to increase for phosphides and nitrides. Given the overwhelming and extensive volume of literature on precious metal free HER catalysts, it is challenging to maintain concision, we have therefore selected a few materials and families of materials to deploy as case-studies for both acidic and alkaline electrolyte.

### 3.3 Acidic HER Electrocatalysts

For acidic HER catalysis, we focus on MoS_2_-based, transition metal phosphide and carbide catalysts and prioritize pioneering work and groundbreaking studies, particularly those that have received significant citations. We also highlight that there are numerous review articles that effectively summarize the detailed findings among specific material classes such as sulfides,^79,310−315^ phosphides,^84,310,311,316−320^ carbides,^310,321^ selenides,^310,311,314^ nitrides,^310,321,322^ and borides.^323,324^

#### 3.3.1 Molybdenum Sulfides

While several sulfide-based materials have been investigated for hydrogen evolution in acidic conditions, the vast majority are molybdenum sulfide-based. The following will focus on the development of such molybdenum sulfide-based catalysts. For other transition metal sulfides the reader is referred to recent reviews.^314,315^

**Figure 14:**
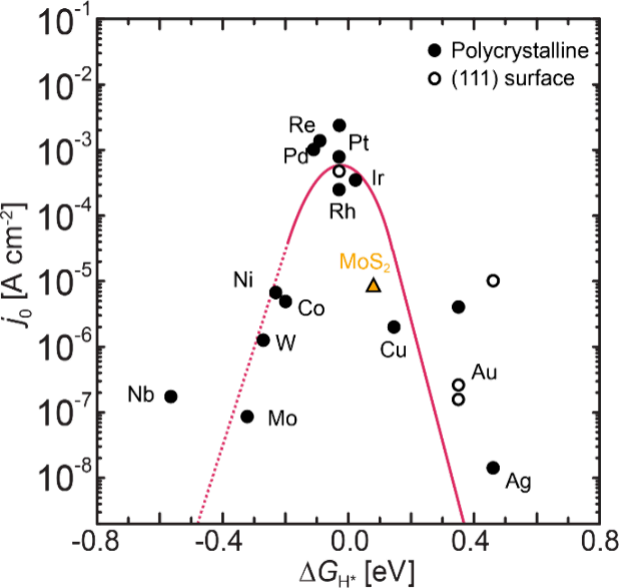
When the exchange current density for MoS_2_ and different metals is plotted against the hydrogen adsorption free energy, a volcano relationship emerges. Reproduced with permissions from ref (327). Copyright 1979 American Association for the Advancement of Science; ref (60). Copyright 2010 American Chemical Society.

The exploration of MoS_2_ as a potential hydrogen evolution reaction catalyst dates back to the 1970s, where Tributsch et al. showed that bulk MoS_2_ crystals were not active for HER.^325^ As a result, MoS_2_ was disregarded as a viable electrocatalyst for HER for a long time. However, Hinnemann et al. in 2005 noticed that the (1010) Mo-edge structure of MoS_2_ showed similarities to the active site of nitrogenase.^326^ In addition, they found that this MoS_2_ edge at a 50% hydrogen coverage possesses a Δ*G*_H_ of 0.08 eV, which is near the optimal value of 0 eV (Figure 14). In stark contrast, the basal plane exhibits a Δ*G*_H_ of 1.92 eV, which places it firmly on the nonbinding side of the volcano explaining the poor activity of bulk MoS_2_ crystals. In an attempt to expose many edges sites, they also synthesized MoS_2_ nanoparticles supported on a carbon black support, and thus provided the first experimental indication that the MoS_2_ edge structure serves as the active site for the HER. At that time, it was the most active precious metal free catalyst for HER reported in acidic conditions with an overpotential of ∼175 mV for 10 mA cm_geo_^–2^ geometric current density. In a subsequent study, Jaramillo et al. prepared single-layer MoS_2_ nanoparticles on a Au(111) surface.^327^ Using scanning tunneling microscopy (STM), the areas and edge lengths of the nanoparticles were measured, and it was found that the HER activity scaled linearly with the perimeter length of MoS_2_, rather than the surface area. The amalgamation of this combined theoretical and experimental research approach yielded the crucial verification that solely the edges of MoS_2_ are catalytically active, inspiring the creation of MoS_2_ catalysts possessing a significant proportion of exposed edge sites.

**Figure 15:**
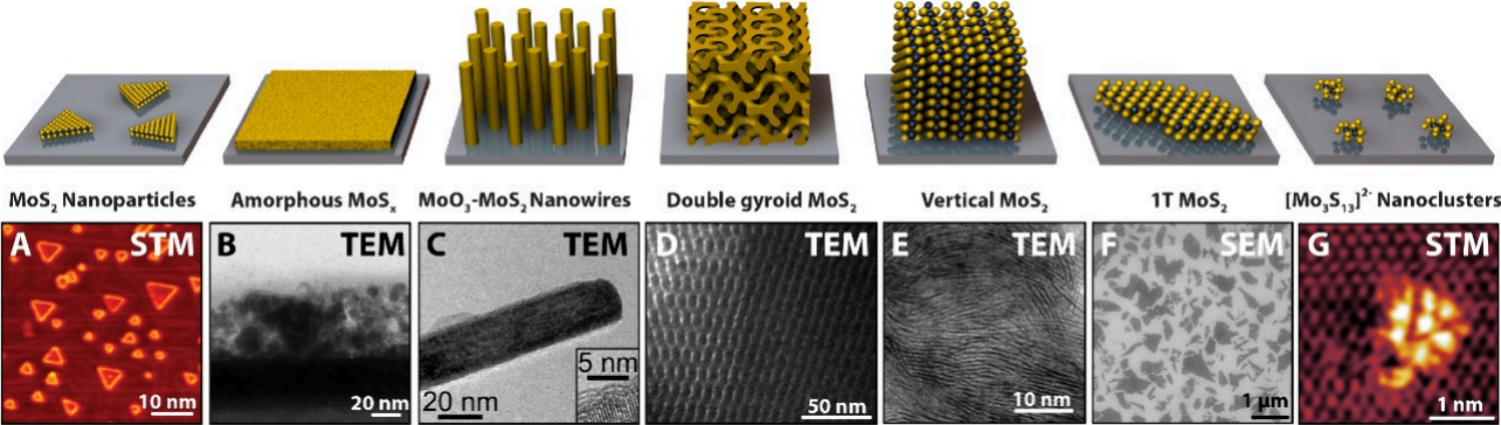
Representative schematics and microscopy images of a variety of MoS_2_-based catalyst morphologies. Microscopy images in panels A–G are reproduced with permissions from ref (327). Copyright 1979 American Association for the Advancement of Science; ref (328). Copyright 2012 Springer Nature; refs (329, 333). Copyright 2011 American Chemical Society and Copyright 2013 American Chemical Society; ref (332). Copyright 2013 the Author(s). Published by PNAS; ref (334). Copyright 2011 Royal Society of Chemistry; ref (335). Copyright 2014 Springer Nature.

A wide range of nanostructuring methods have been subsequently deployed to engineer various morphologies, which maximize the number of MoS_2_ edge sites exposed (Figure 15). One such example is a three-dimensional mesoporous MoS_2_ nanostructure with a double-gyroid morphology.^328^ The nanoscale curvature of the double-gyroid structure reduces the formation of extended basal planes, resulting in a high density of active edge sites. Consequently, the turnover frequency averaged across all surface sites (TOF_avg_) of double-gyroid MoS_2_ was 2 to 4-times higher than that of MoO_3_–MoS_2_ nanowires prepared using a similar sulfidation technique.^329^ However, the double-gyroid structure has the disadvantage of a long electron transport distance from the conductive substrate to the active site, which leads to increased resistive loss. The underlying cause of the increased resistive losses is the fact that the electron mobility perpendicular to the MoS_2_ basal planes is about 3 orders of magnitude lower than the in-plane electron mobility.^330^ To address the conductivity issue, Kong et al. synthesized vertically aligned MoS_2_ nanostructures, which not only exposed a large number of edge sites but also facilitated easy electron transport to the conductive substrate.^331,332^

Nanoparticles supported on high-surface area supports have been another popular route to facilitate a high number of edge-sites. Early examples here are MoS_2_ nanoparticles synthesized on reduced graphene oxide nanosheets,^336^ amorphous MoS_*x*_ layers directly bonded on vertical N-doped carbon nanotubes^337^ and ultrathin MoS_2_ on nitrogen-doped reduced graphene oxide.^338^

Amorphous molybdenum sulfides have also been investigated for the HER and often possess high overall electrode activities due to their high surface areas.^334,339,340^ For some applications, e.g., where heat-sensitive supports are used, amorphous molybdenum sulfides may be particularly advantageous, since they can be synthesized directly by electrodeposition or wet chemical reactions without thermal sulfidation treatment. Through physical and chemical characterization, it has been discovered that the amorphous material, in its initial synthesized state, exhibits a composition closely resembling MoS_3_. However, when a cathodic potential is applied, a transformation occurs, which alters the surface composition to MoS_2_.^341^

Two major conclusions from the early MoS_2_ research are that the thermodynamically stable semiconducting 2H-MoS_2_ phase suffers from a catalytically inert basal plane and limited conductivity. However, other polymorphs of MoS_2_ have been considered to mitigate these limitations, and special emphasis has been placed on the metallic 1T-phase. The 2H-MoS_2_ phase can be transformed into the 1T-MoS_2_ phase by intercalation of alkali ions, which has been known for decades.^342−344^ Early investigations by Lukowski et al. suggested that an observed increased HER activity of the 1T-phase over the 2H-phase is due to an increased density of active sites and improved charge transfer resistance of the metallic phase.^345^ Subsequent investigations have suggested that the electronic conductivity of 1T-MoS_2_ is ∼10^5^ higher than that of 2H-MoS_2_.^346^ Voiry et al. studied the nature of the active site on 1T-MoS_2_ by partially oxidizing both the 2H-phase and 1T-phase of MoS_2_.^333^ The results showed that the catalytic activity of the 2H-phase significantly decreased following oxidation, whereas the 1T-phase remained unaffected even after oxidation. Based on these findings, they proposed that the edges of 1T-phase MoS_2_ nanosheets are not the primary active sites and suggested that the basal plane could potentially be catalytically active. The significantly larger active surface area of 1T nanosheets with respect to the edge portion thus ensures enhanced HER activity. Since the first investigations of 1T-MoS_2_, much of the research into MoS_2_ as an HER catalyst has been directed toward the 1T-phase, not least improving the stability since 1T-MoS_2_ is only metastable and can revert to the stable 2H-phase,^347−349^ and specialized reviews on the 1T-phase are available.^350,351^

Although several experimental studies have investigated the reaction mechanism and pathway for MoS_2_-based HER catalyst, these are extremely challenging measurements to interpret.^44,352^ Theoretical studies have investigated the prominent mechanism on various MoS_2_-based materials, including theoretical studies probing doped 1T-MoS_2_ materials,^353^ the role of sulfur vacancies on the basal plane of MoS_2_,^354^ 2H/1T interfaces.^355,356^ These efforts have been reviewed within sections of several published reviews.^46,253,357^

The basal plane of 2H-MoS_2_ can also be activated by the introduction of defects. Li et al. theoretically predicted and experimentally demonstrated that strained S-vacancies in the basal plane of 2H-phase monolayer MoS_2_ are active catalytic sites for HER.^358,359^ The effect of the density of sulfur vacancies was further investigated and regions with a high concentration of surface S vacancies leading to undercoordinated Mo atoms were found to have higher activity than regions with low concentrations of surface “point-defect” S vacancies.^308^ Grain-boundaries in MoS_2_ have also been shown to possess catalytic activity.^360,361^ Li et al. experimentally ranked the intrinsic turnover frequencies toward HER as TOF(edge sites) > TOF(sulfur vacancies) > TOF(grain boundaries).^360^

Another strategy deployed to tune the HER activity of MoS_2_ is through doping of heteroatoms. Theoretical investigations have suggested that dopants can fine-tune the adsorption energy of hydrogen, thus promoting the HER activity.^362,363^ Several different metals have been tried experimentally. For example, cobalt doping of amorphous molybdenum increased the active surface area albeit not the intrinsic activity.^364^ However, other studies have found that single Co-atoms in 1T-MoS_2_^365^ and CoMoS_*x*_ chalcogel structures^366^ increases the activity. Several studies also investigated the effect of vanadium-doping and find increased activity over similar prepared undoped MoS_2_.^367−369^ Doping with nonmetals like nitrogen,^370^ oxygen^371^ and phosphorus^372^ has also been suggested to facilitate structural changes and improve the conductivity of MoS_2_.

Finally, molybdenum sulfide nanocluster compounds, which serve as a bridge between molecular and solid-state electrocatalysis when supported on electrode surfaces, represent another intriguing class of catalysts. These inorganic clusters feature an abundance of undercoordinated sulfur atoms on their surfaces resembling the edges of MoS_2_. Examples of these small clusters are incomplete [Mo_3_S_4_]^4+^ cubanes^373,374^ and thiomolybdate [Mo_3_S_13_]^2–^ nanoclusters.^240,335,375−377^

#### 3.3.2 Phosphides

**Figure 16:**
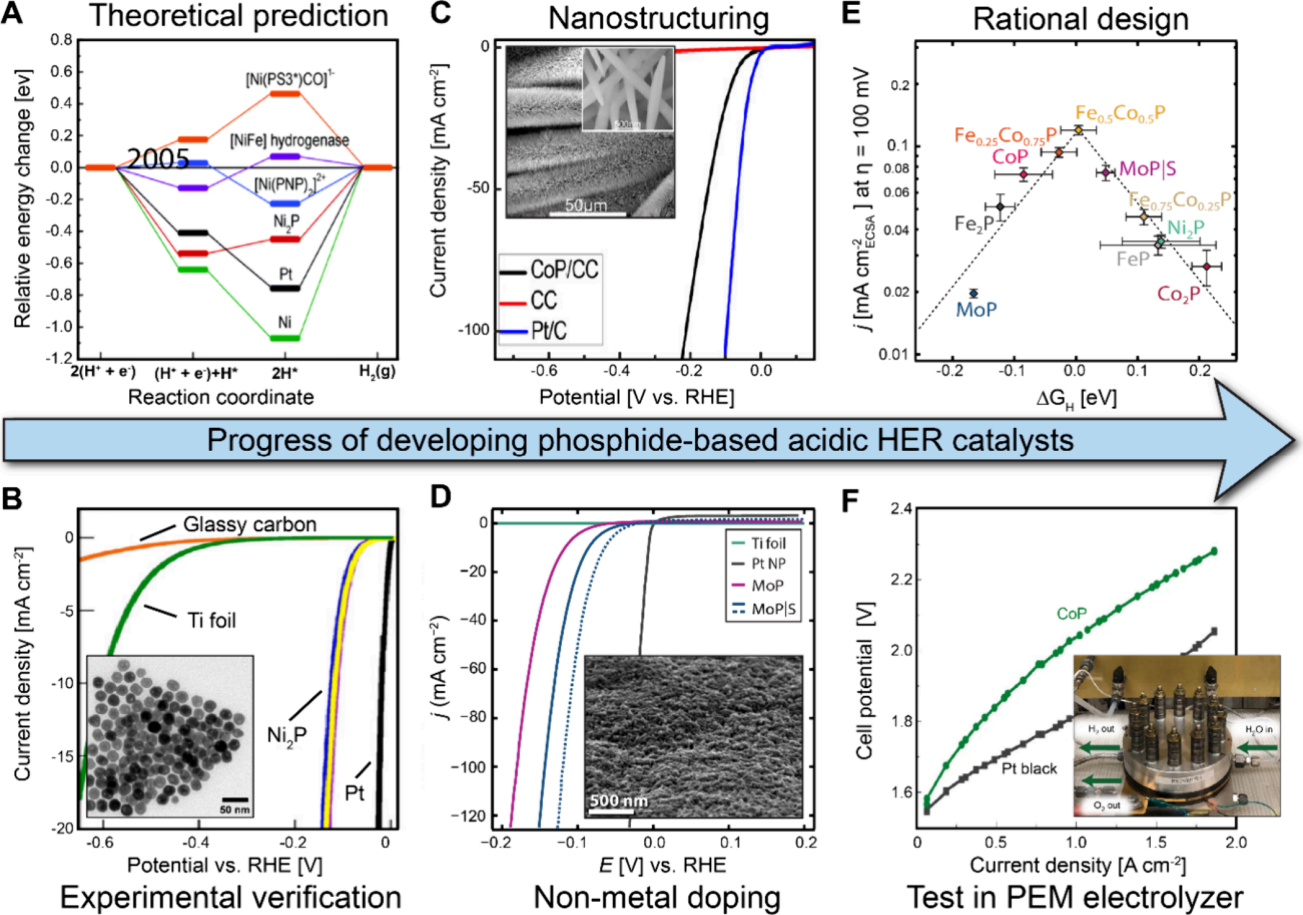
Selection of pioneering and highly cited work highlighting the progress of phosphide-based HER catalysts. A) Theoretical prediction of Ni_2_P as an HER catalyst. B) Experimental verification of Ni_2_P as a good HER catalyst. C) Nanostructuring to increase the surface area and thus number of active sites. D) Doping by anion (e.g., sulfur) substitution, E) Rational design of new phosphide-based catalysts. F) Testing in a commercial-scale PEM electrolyzer. Panels A–F are reproduced with permissions from refs (380, 381, 384). Copyright 2005 American Chemical Society, Copyright 2022 American Chemical Society, and Copyright 2014 American Chemical Society; ref (95). Copyright 2014 Wiley-VCH; ref (64). Copyright 2015 Royal Society of Chemistry; ref (156). Copyright 2019 Springer Nature.

Transition metal phosphides are among the most investigated precious metal free catalysts for HER (Figure 13). While a few early papers saw hydrogen evolution in alkaline conditions on NiP electrodes,^378,379^ the beginning of the acidic phosphide field is often ascribed to the theoretical DFT study by Liu and Rodriguez who, guided by theoretical calculations on [NiFe] hydrogenase and its molecular analogues, predicted Ni_2_P(001) to be a good HER catalyst (Figure 16A).^380^ Although this increased the level of interest in phosphide A-WE research, it is worth noting that this finding is more likely applicable to acidic electrolytes as the H-binding energy is not a suitable stand-alone descriptor for evaluating HER activity in alkaline environments, as described in Section 1.3. Nonetheless, the prediction was verified by Popczun et al., who demonstrated that Ni_2_P nanoparticles supported on a Ti foil have excellent HER activity in both acidic and alkaline electrolytes (Figure 16B).^381^ At the same time FeP nanosheets were also found to exhibit good HER activity.^382^ Soon after, a multitude of different transition metal phosphides were investigated, including: CoP,^383,384^ Co_2_P,^385^ Ni_5_P_4_,^386^ Cu_3_P,^387^ MoP,^95,388,389^ and WP.^390^

Transition metal phosphides exist in an extensive range of stoichiometries spanning binary, ternary, and higher-order metal phosphides. Thus, not only does the choice of the transition metal affect the catalytic activity but also the phosphorus-to-metal ratio. In general, several studies suggest that a higher phosphorus-to-metal ratio is beneficial for the HER activity. For example, Pan et al. prepared different nanostructured nickel phosphide and compared their activities for HER under similar conditions and found that the trend of increasing activity follows: Ni_5_P_4_ > Ni_2_P > Ni_12_P_5_.^391^ Similar finding were reported by Laursen et al., who showed Ni_5_P_4_ to be more active than Ni_2_P,^386^ and Kucernak et al., who found Ni_2_P to be more active than Ni_12_P_5_ and further showed that materials with higher phosphorus content are more corrosion resistant.^392^ Similarly, CoP was found to exhibit a higher activity than Co_2_P,^393,394^ and MoP better activity than Mo_3_P.^389^

To rationalize general trends in HER activity, Kibsgaard et al. studied a wide range of different transition metal phosphides in a combined experimental–theoretical approach.^64^ The HER activity was found to follow a volcano relationship (Figure 16E) when the hydrogen adsorption free energy was used as descriptor. Based on the combined experimental–theoretical model, a mixed metal phosphide, Fe_0.5_Co_0.5_P, should have a near-optimal Δ*G*_H_ and was found exhibits the highest HER activity in the study. In a later study Tang et al. also investigated Fe_*x*_Co_1–x_P-based catalysts with a nanowire morphology in a combined experimental–theoretical work and confirmed Fe_0.5_Co_0.5_P to have the best HER activity.^395^

Another approach investigated to enhance the hydrogen evolution activity of transition metal phosphides is anion substitution. Kibsgaard and Jaramillo saw that introduction of sulfur into a molybdenum phosphide to form a phosphosulfide, MoP|S, led to significant improvements in the electrocatalytic HER activity over MoP films (Figure 16D).^95^ Similarly, Cabán-Acevedo et al. found cobalt phosphosulfide (CoPS) was more active than CoP_*x*_ (CoP with a minor CoP_2_ phase impurity).^396^

Similar to the sulfide-based catalysts, a major effort in the development of phosphide-based catalyst has been centered on increasing the number of active sites through high-loadings and nanostructuring. An early example is a nanoporous cobalt phosphide nanowire array reported by Tain et al.,^384^ and several studies have since then reported sub-50 mV overpotentials for 10 mA cm_geo_^-2^.^386,397−402^ Albeit some of these studies do not obey best practices,^49^ such as avoiding a platinum counter electrode and ensuring a H2-saturated electrolyte (discussed in Section 2.2.3), the reported low overpotentials show why phosphides have been considered as one of the best candidates to replace platinum.).

Most of the work on developing phosphide-based HER catalysts have been performed in small-scale laboratory setups. As discussed in Section 2.4, observations made with idealized half-cell laboratory settings do not necessarily translate into high performance upscaled devices necessitating tests in real PEM electrolyzer setups. King et al. tested the performance of a CoP-based catalyst in commercial-scale 86 cm^2^ PEM electrolyzer and showed >1,700 h of stable operation at elevated temperature and pressure, while paying a 12–18% power penalty under the operating conditions as compared to a platinum-based PEM (Figure 16F).^156^

#### 3.3.3 Carbides

**Figure 17:**
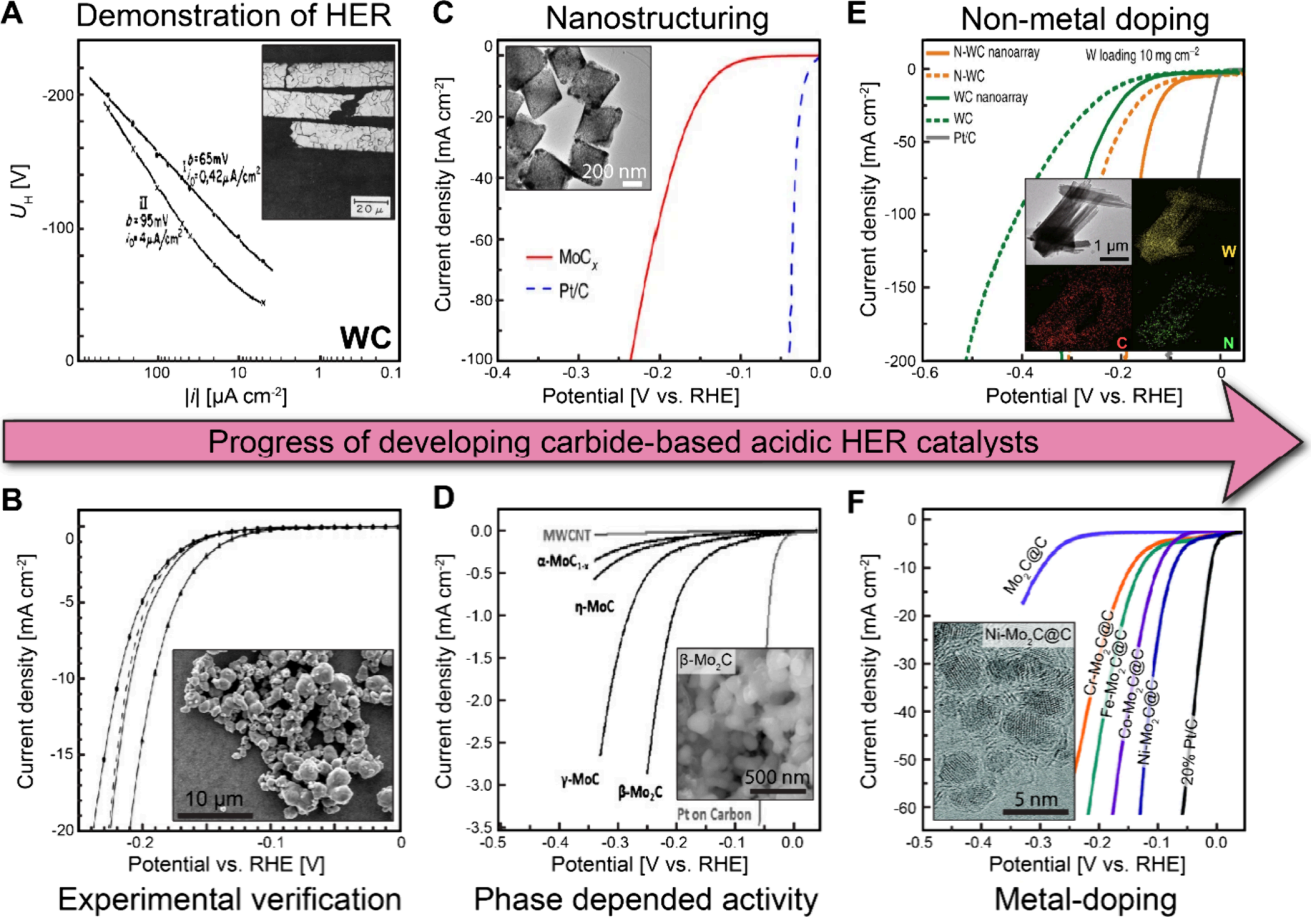
Selection of pioneering and highly cited work highlighting the progress of developing carbide-based HER catalysts. (A) Experimental demonstration of HER activity on tungsten carbide. (B) Demonstration of Mo_2_C as a good HER catalyst. (C) Nanostructuring to increase the surface area and thus number of active sites. (D) Demonstration of phase-depended HER activity. (E) Increasing HER activity with nonmetal doping. (F) Tuning of HER activity by introducing metal dopants. Panels A–F are reproduced with permissions from ref (405). Copyright 1970 Elsevier; refs (406, 407). Copyright 2012 Wiley-VCH and. Copyright 2014 Wiley-VCH; refs (408, 409). Copyright 2015 Springer Nature and. Copyright 2018 Springer Nature; ref (410). Copyright 2018 Royal Society of Chemistry.

The study of carbides for the HER is thought to have been inspired from thermal catalysis in 1970s, when Levy and Boudart^403^ found that the addition of carbon to non-noble metals (e.g., W and Mo) can lead to catalytic behaviors that only noble metals (e.g., Pt, Pd) otherwise show. Specifically, this study focused on 3 reactions for which Pt shows reasonable activity, but W metal is catalytically inactive, i.e., chemisorption of hydrogen, H_2_ dissociation and isomerization of 2,2-dimethylpropane. Interestingly, these reactions can take place at WC, although with lower activities than Pt. Levy and Boudart thus postulated tungsten in WC has a Pt-like electronic structure, and the possible descriptor for isomerization of 2,2-dimethylpropane was electronegativity. In addition to WC, Mo_2_C behaves more like a noble metal than Mo for ethane hydrogenolysis. Since then, many studies and reviews have been reported on the electronic and structural properties of carbide surfaces and their thermocatalytic properties.^404^ As an early attempt, in 1970, WC was tested as an electrocatalyst for acidic HER/HOR albeit having lower activity than Pt (Figure 17A).^405^ These early observations indicated that combining non-noble metals with carbon to form carbides, might be a promising way of replacing precious metal catalysts for some reactions, including the HER.

HER studies on carbides accelerated during the 2010s. The pioneering work from Hu’s group measured HER activities of β-Mo_2_C in both acid and alkaline, which inspired great efforts to develop various carbides (M_*x*_C, M = Mo, W, Co, V, Ta, Fe, Ni, Ti, etc.), with Mo_2_C as the most widely reported catalyst (Figure 17B and C). Here we briefly introduce the designing strategies, including crystal phase, nanostructure, support materials, and doping. For more detailed analysis and examples, we refer readers to previous reviews.^3,310,411,412^ The HER activity can vary with crystal structures, as exemplified by molybdenum carbides, whose geometric activities follow the order α-MoC_1–*x*_ < η-MoC < γ-MoC < β-Mo_2_C (Figure 17D).^407^ Similarly, W_2_C is more active than WC.^408^ Nanostructuring carbides into nano- or 2D-morphologies^408^ can enhance HER activity by increasing the number of surface active sites (Figure 17C). As the formation of carbide phase requires a high temperature, nanostructuring is usually with the aid of a supporting material, such as graphitized carbon shells,^407^ carbon nanotubes (CNTs).^413^ The HER activity of carbides can be tuned by introducing a dopant (Figure 17E and F), which could be either a metal (Fe, Co, Ni, etc.) or nonmetal elements (N, S, B, P and O). Examples can be found on Ni-, Co-, Fe-, Cr-doped Mo_2_C,^410^ P-doped Mo_2_C,^305^ N-doped WC.^409^ Doping the carbon supporting material has also been explored, e.g., N-doped CNTs^414^ and carbon nitrides^415^ as a strategy to further increase the HER activity. Varying the termination atom on carbide surface has also been explored. For example, terminating carbide surfaces (Ti_2_C and Mo_2_C) with OH and O can tune the binding energy of hydrogen to the surface.^67^

As a result of these design strategies, the mass activity and geometric activity of carbides have been driven closer to Pt. However, the intrinsic activity of carbides, relative to Pt, remains barely improved over these years, indicating these designing principles fail to break some limitation originating from the intrinsic difference between Pt and carbide surface. Possibly, carbides have the same problem as 2D-transition-metal dichalcogenides,^416^ whose activation energies of HER rate-determining step scale with Δ*G*_H_ (hydrogen adsorption energy) in exactly the same fashion as metals but systematically shifts ∼0.4 eV higher.

#### 3.3.4 Activity Metrics

**Figure 18:**
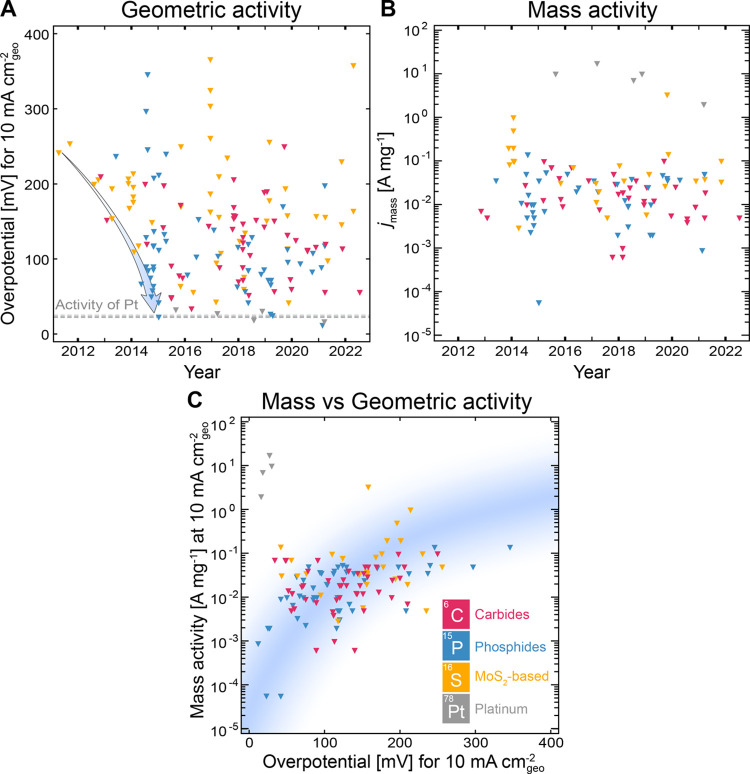
(A) Chronological trend in overpotential for a geometric current density of 10 mA cm^–2^_geo_ of carbide, phosphide and MoS_2_-based HER catalysts assessed in acidic electrolyte. Pt-based HER catalysts are shown for comparison; the dashed line is the average value of the data points. Blue arrow to guide the eye. (B) Chronological trend in the mass activity (evaluated at *j* = 10 mA cm^–2^_geo_) of the catalysts in (A). (C) Correlation of the mass activity in B and geometric activity in A. Shading to guide the eye. The data used in this figure and associated references are provided in the Supporting Information.

During the first few years of exploration, the geometric activity of precious metal free catalysts for HER under acidic conditions showed great improvement, as seen from Figure 18A where the chronological progression of overpotential for 10 mA cm_geo_^-2^ is shown. However, it is also clear from the figure that since 2015–2016, the reported overpotentials have reached a point of stagnation, exhibiting negligible advancements from then on and essentially have approached the geometric performance levels of platinum. Consequently, the pursuit of further improvements in this regard appears to be unnecessary. However, the improvement in geometric activity has to a large extent been driven by increasing the number of active sites often accompanied by an increased catalyst loading. Consequently, the improvements observed in geometric activity have not translated into improved mass activity as seen from Figure 18B, which shows the chronological trend in the mass activity of the catalysts in Figure 18A. When plotting the mass activity as a function of geometric activity it is evident that the overall trend is that improvements in geometric activity are indeed accompanied by a decreased mass activity as seen by the blue shaded guide to the eye in Figure 18C. The approach of significantly increasing the mass loading to enhance geometric activity may ultimately prove not to be viable, since the catalyst cost in the end may be higher than a platinum-based catalyst. Therefore, solely evaluating a catalyst based on its geometric activity can provide a misleading impression of its suitability for large-scale applications.

**Figure 19:**
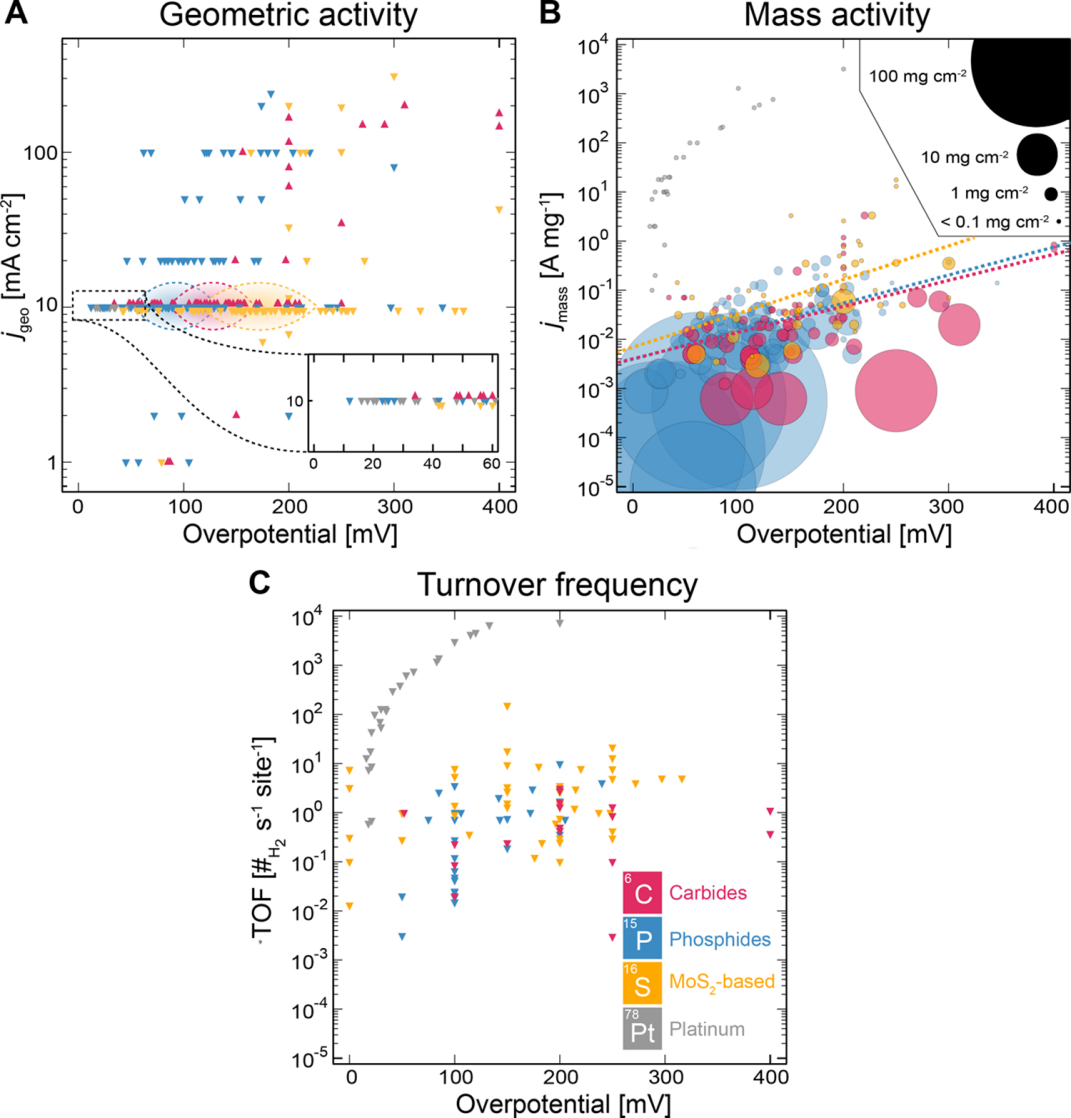
Plot of (A) geometric activity (B) mass activity and (C) urnover frequency (TOF) of carbide-, phosphide- and MoS_2_-based HER catalysts as a function of overpotential. Data for platinum is also shown for reference. (A) Notice data for carbides/MoS_2_-based catalyst at 10 mA cm^–2^ have been shifted slightly up/down, respectively, to limit overlap of data points. The blue, red and yellow shaded areas are contour plots encompassing 50% of the 10 mA cm^–2^ data points for phosphides, carbides and MoS_2_-based catalyst, respectively. (B) The areas of the data points are proportional to the mass loading per cm^2^. To ensure visibility a lower minimum data point size has been imposed on points with extremely low mass loading, e.g., some sulfide and Pt points. The dashed lines represent linear fits to the data points. Notice data points with extreme mass loadings above 30 mg cm^–2^ have been excluded from the fit. The data used in this figure (including mass loadings) and associated references are provided in the Supporting Information.

To further explore activity trends and compare with state-of-the-art platinum for HER, we have collected a large set of reported activity data from the literature that includes multiple geometric current densities beyond 10 mA cm^–2^_geo_. The resulting plots are shown in Figure 19, which summarizes the past decade of research into carbide-, phosphide- and MoS_2_-based HER catalysts for acidic conditions. While some of these studies do not follow best practices (as described in Section 2.2.),^49^ and the absolute position of individual point thus can be questioned, trends from the collective information can be discerned.

Figure 19A shows reported geometric activities as a function of overpotential. It is evident that the most commonly reported geometric activity is at 10 mA cm_geo_^–2^. The insert (zoom-in) confirms that some precious metal free catalysts, especially phosphide-based, have essentially achieved platinum-like HER activity on a geometric basis when measured at a current density of 10 mA cm_geo_^–2^. Statistically, phosphides in general report lower overpotentials than carbides and MoS_2_-based catalysts as seen from the blue, red and yellow shaded areas in Figure 19A, which are contour plots encompassing 50% of the 10 mA cm^–2^ data points for phosphides, carbides and MoS_2_-based catalyst, respectively.

As previously described, the improvements in geometric activity are overwhelmingly associated with increased mass loadings and a corresponding decrease in mass activity, as is evident in Figure 19B where the area of the data points have been scaled to reflect the mass loading. Note that a lower minimum data point size has been imposed on points with extremely low mass loading, e.g., some sulfide and Pt points, to ensure visibility in the plot. The actual mass loadings and other data can be found in Supporting Information along with the associated references from where the data have been collected. From Figure 19B it is clear that the mass activity of precious metal free catalysts are still 3–4 orders of magnitude lower than that of platinum. Interestingly, the dashed lines in Figure 19B represent a linear fits to the data points (excluding data points with extreme mass loadings above 30 mg cm^–2^) and the coincidence shows that carbide-, phosphide- and MoS_2_-based HER catalysts largely follow the same trend in mass activity as a function of overpotential. The line for the MoS_2_-based catalysts is slightly higher than the carbide and phosphide lines, which could be a result of typically lower loadings, as seen from the generally smaller data point size for the MoS_2_-data. This coincidence could suggest almost identical intrinsic activity. Scientifically, the relevant metric to evaluate intrinsic activity is the turnover frequency (TOF), i.e., the number of evolved H_2_ molecules per s per active site at a specific overpotential. Figure 19C plots the TOF of the carbide-, phosphide- and MoS_2_-based HER catalysts. Compared to state-of-the-art Pt the precious metal free catalyst are ∼3–4 orders of magnitude lower. Notice the MoS_2_ TOFs at 0 mV are based on extrapolated exchange current density values,^331,358,360^ which can shift the TOF to higher values compared to TOFs directly measured at overpotentials close to the reversible potential where the backward reaction can be significant. While being the scientifically most relevant metric, as discussed in Section 2.1, the TOF is much less often reported, likely due to the difficulty in accurately measuring the electrochemically active surface area. This difficulty also likely plays a substantial role in the significant scatter in the reported TOF values as observed in Figure 19C.

The difference in intrinsic activity between platinum and the precious metal free catalysts in general is possibly linked to commonalities in the activation energies of HER rate-determining step of precious metal free catalysts. For 2D-transition-metal dichalcogenides it was shown that the activation energy of the HER rate-determining step scale with Δ*G*_H_ (hydrogen adsorption energy) in exactly the same fashion as metals but systematically shifts ∼0.4 eV higher.^416^ Thus, even for precious metal free catalyst with an optimal Δ*G*_H_ ≈ 0 similar to Pt, the higher activation energy of the rate-determining step limits the HER activity.

It is clear from our review of precious metal free HER catalyst literature that a primary driver for development has been the geometric activity metrics. And while the obvious catalyst to compare precious metal free HER catalysts up against is platinum, we recommend shifting the focus from attempting to achieve “platinum-like” geometric activity at the cost of extreme mass loadings, to focus on mass activity and intrinsic activity of precious metal free catalysts. The open question remains whether precious metal free catalysts can be tailored to demonstrate true platinum-like activity on a turnover frequency basis.

### 3.4 Alkaline HER Electrocatalysts

**Figure 20:**
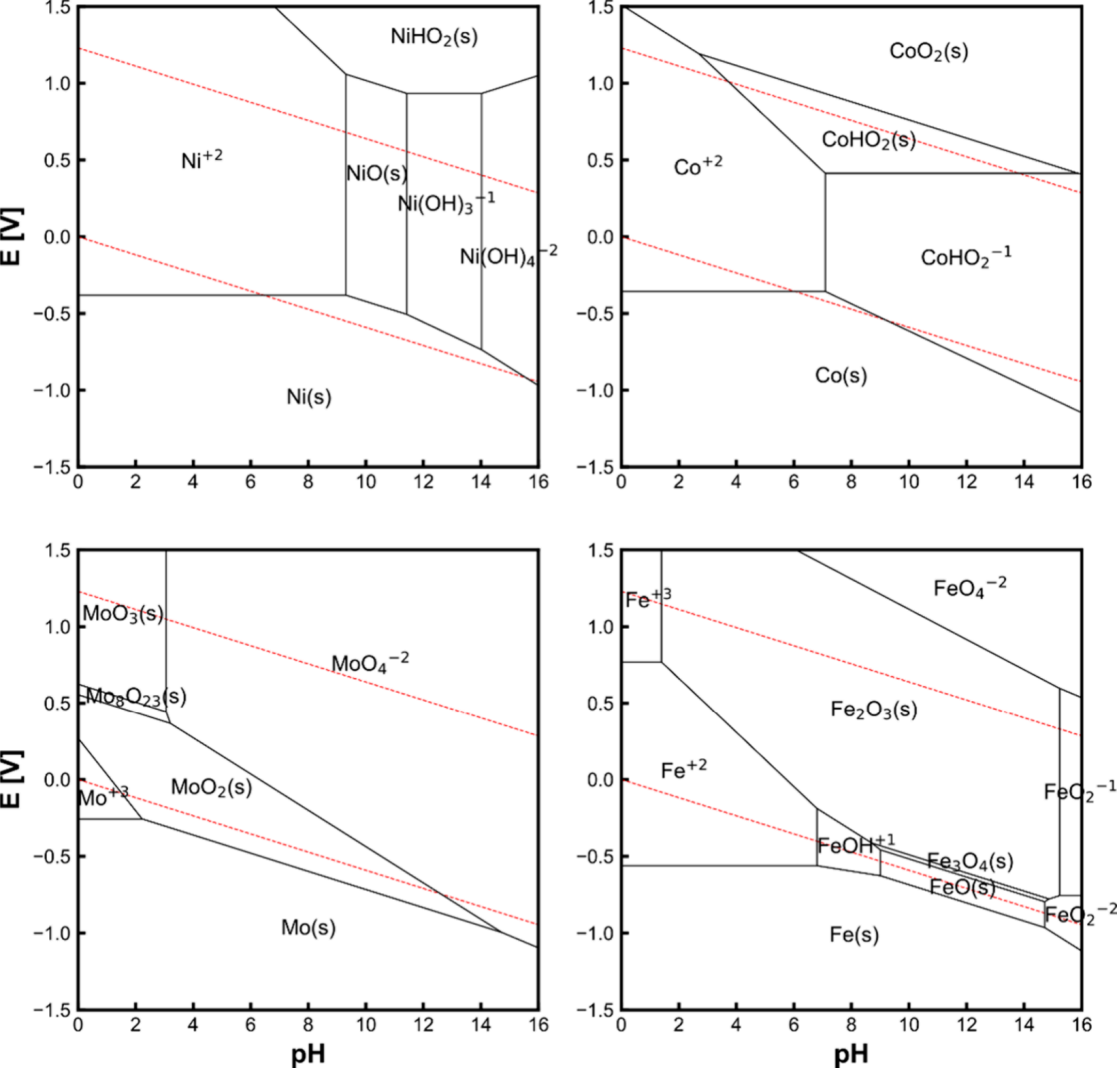
Pourbaix diagrams for the four most common metals used for HER in A-WE. At pH = 14, [Metal] = 10^–6^ M, and 0 V vs RHE, only Ni and Co show stable metallic phases. Ni, Co, and Mo have dissolving phases at or >0.1 V vs HER, leading to the assumption that these materials will dissolve if cycled from HER to >0.1 V vs HER. All four metals (Fe, Co, Ni and Mo) show instability regions between HER and OER at pH = 14. The figures were generated using the Materials Project API.^417,419,421−423^

**Figure 21:**
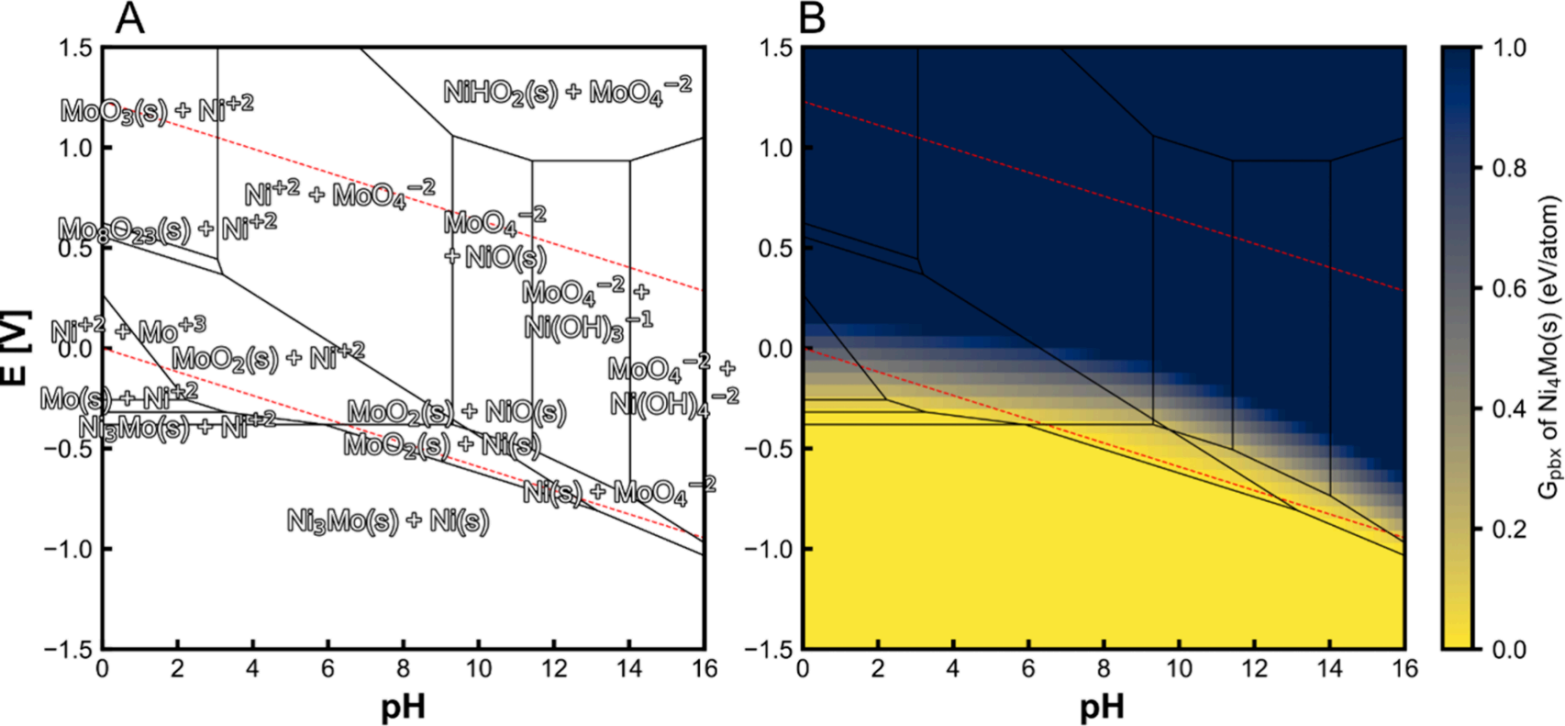
Pourbaix diagram for the Ni–Mo system. Without (A) and with (B) a heat map overlay depicting the theoretical likelihood of metastability of Ni_4_Mo. A) The Pourbaix diagram for the Ni–Mo system, which shows the thermodynamically stable phases. B) The heat map quantifies the Gibbs free energy differences between Ni_4_Mo and the thermodynamically stable phases across various pH and applied potentials. Analysis of the heat map reveals that Ni_4_Mo is likely metastable at HER conditions (the yellow areas). However, it is prone to degrade upon oxidation beyond the water reduction line into the regime marked in blue. The ion concentration for both elements in each diagram is set at 10^–6^ M. The Ni_4_Mo entry ID is mp-11507.^419,421−423^

The primary advantage of alkaline water electrolysis (A-WE) over PEM is the broader range of materials that can maintain stability within the electrolyte. Indeed, this is illustrated in Figure 20 where Pourbaix diagrams for the four metals most common in A-WE for HER is shown. These diagrams are valuable for understanding the stability regions of various metals and alloys, but they do not always convey the entire picture. For example, nickel was predicted to corrode between approximately 100–800 mV vs RHE at pH 14,^417^ yet it remains stable and plays a critical role in commercial A-WE components/catalysts.^418^ Pourbaix diagrams show only the thermodynamically stable phases, another tool is needed to assess the stability of metastable phases. In 2017, Singh et al., implemented a theoretical framework for calculating the thermodynamical driving force for degradation of metastable phases and implemented the calculation tool into the Materials Project tool at https://materialsproject.org.^419^ The tool works by visualizing the thermodynamic driving force for decomposition of one’s specific phase, via a heat map of the phase’s relative Gibbs free energy, Δ*G*_pbx_, compared to the thermodynamically favored phase(s) at various pH and voltage. Figure 21 provides an example of this, with an heat map overlay of Ni_4_Mo on the Ni–Mo Pourbaix diagram. Singh et al., state that when a phase’s Δ*G*_pbx_ is less than 0.5 eV/atom it is likely metastable at that given pH and potential. A higher driving force renders metastability unlikely. This tool bridges corrosion science and thermodynamical data, and may aid in the selecting new catalysts to study. It may also help elucidate what happens when an HER catalyst is submerged in the electrolyte under applied potentials, which is notoriously difficult to study.^420^

In this section, we review nickel molybdenum and transition metal phosphides as alkaline electrocatalysts for HER, providing a succinct review of notable studies. Detailed reviews of nickel molybdenum, transition metal phosphides, and more general reviews in alkaline electrolyte have been published elsewhere.^47,424−428^

#### 3.4.1 Nickel Molybdenum

**Figure 22:**
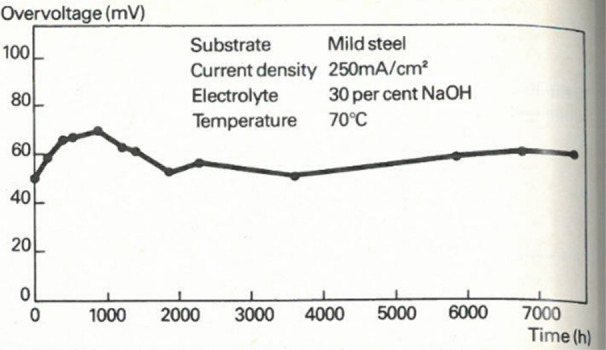
A chronopotentiometric measurement of Ni_*x*_Mo_*y*_ coated mild steel in 30 wt % NaOH at 250 mA/cm^2^ run for 7500 h showcasing long-term activity toward HER for the treated catalysts. Figure reproduced with permission from ref (430). Copyright 1984 John Wiley and Sons.

Ni_*x*_Mo_*y*_ is arguably the most renowned catalyst family for nonprecious metal HER under alkaline conditions, although it has, to the best of our knowledge, not yet reached commercial application. Interest in this family of materials was originally sparked by industrial research carried out by British Petroleum in the early 1980s.^429^ Their objective was to discover inexpensive and efficient catalysts for alkaline HER in the context of the chlor-alkali industry. The first reported Ni_*x*_Mo_*y*_ samples were synthesized by Mahmood et al. by spray and dip pyrolysis of nickel and molybdenum salts, creating oxide coatings which were subsequently reduced in hydrogen atmospheres.^429^ Compared to pure nickel’s overpotential of 455 mV at identical conditions, these samples demonstrated markedly lower overpotentials of 84 mV vs RHE to achieve a current density of 1 A cm^–2^ and 70 °C. This early evidence highlighted the significant overpotential reductions that could be achieved in the water-splitting reaction using Ni_*x*_Mo_*y*_. Within two years of these initial studies, stability tests conducted on Ni_*x*_Mo_*y*_ coated mild steel displayed stable operation for approximately 7500 h at a current density of 250 mA cm^–2^, with an overpotential ranging between 50 and 70 mV versus RHE (70 °C, 30 wt % NaOH), as shown in Figure 22.^430^

A variety of synthesis methods to produce Ni_*x*_Mo_*y*_ catalysts of different compositions exists with notable methods including ball-milling metal powders,^431^ electrodeposition,^432^ incorporation of Mo in Raney-Ni,^432^ and self-templating hydrothermal reaction.^433−437^

To date, the active site of Ni_*x*_Mo_*y*_ catalysts remains largely elusive, which is part of a continued debate about the intrinsic activity enhanced of Ni_*x*_Mo_*y*_ versus pure Ni.^438^ These discussions likely stem from the observation that many Ni_*x*_Mo_*y*_ synthesis methods generate very high surface areas, complicating the differentiation between improvements in intrinsic catalytic activity and those arising from a higher density of active sites. As an example, Zu et al. directly grew NiMo_2_ on high surface area porous carbon nanorods, and reported an η_10_ of 179 mV with a catalyst loading of 0.50 mg cm^–2^ and an ECSA of 11.2 mF cm^–2^.^439^ Indeed, most enhancements in Ni_*x*_Mo_*y*_ overpotential are likely attributable to an increased surface area, achieved either through higher catalyst loadings or synthesis optimizations that yield a higher density of material on the electrode surface.

Schalenbach et al. attempted to address this experimental issue by comparing arc-melted samples of Ni, Mo, NiMo, Ni_2_Mo, Ni_3_Mo, Ni_4_Mo, and Ni_6_Mo, with identical electrochemical surface areas, concluding that the activity benefits of alloying Ni with Mo are indeed limited.^147^ Conversely, results from McKone et al. show, on a mass-specific basis, that Ni_*x*_Mo_*y*_ nanopowders are, in fact, more active per surface atom than their pure Ni counterparts.^440^ Moreover, Schiller et al. found that by integrating Mo into a Raney Ni framework, the overpotential was substantially reduced, from 134 mV to 60 mV, at a current density of 1000 mA cm^–2^_geo_ at 70 °C in 25 wt % KOH.^441^

**Figure 23:**
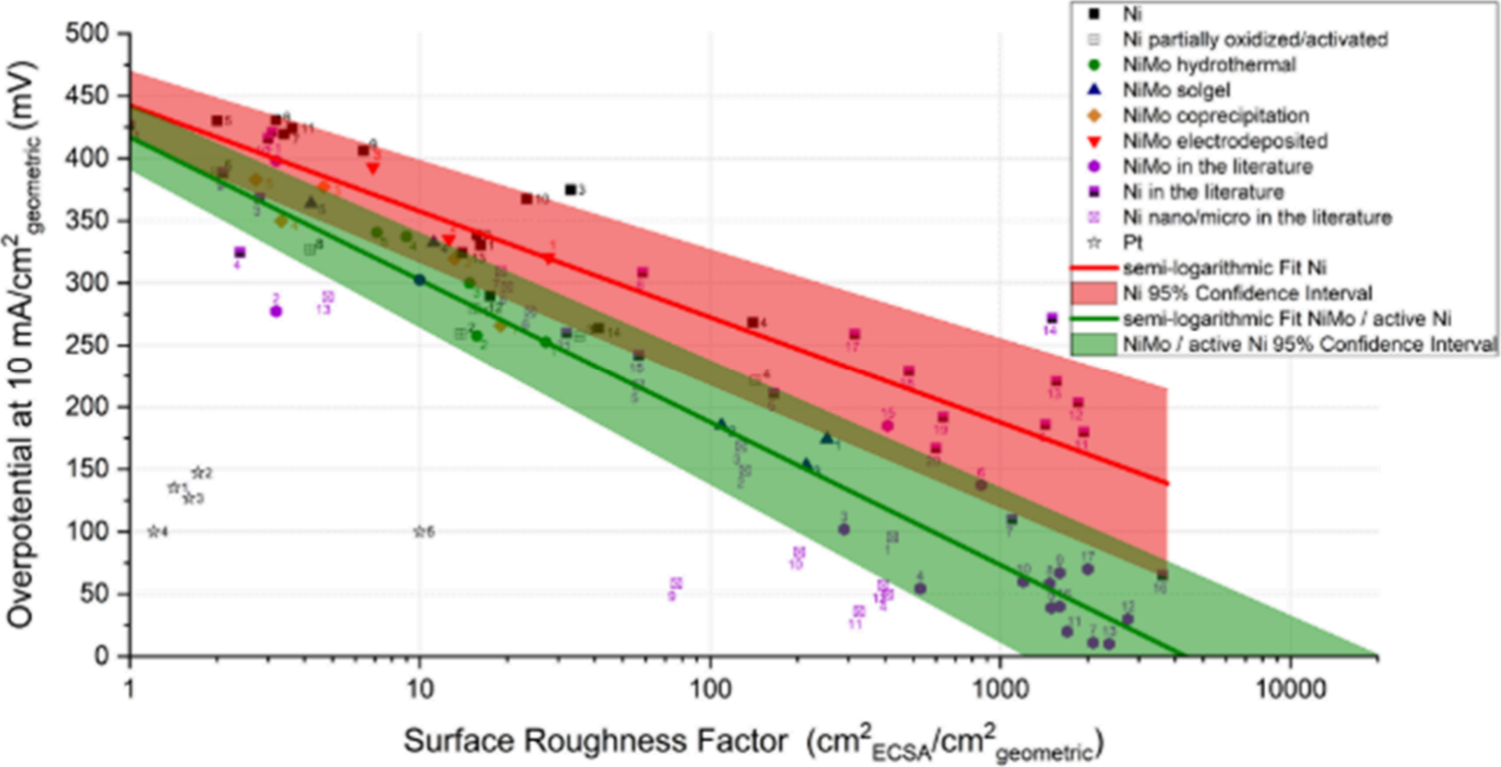
Plot of Ni_*x*_Mo_*y*_ and Ni overpotentials versus their ECSA divided by the electrode geometric area. The green and red lines and shaded areas are semilogarithmic fits of the activity versus surface roughness factor and the 95% confidence interval, illustrating that data from the literature also indicate that Ni_*x*_Mo_*y*_ is intrinsically more active than Ni. Figure reproduced with permission from ref (442). Copyright 2021 F Gellrich at the Technical University of Denmark.

To examine the trends between overpotential and surface roughness, Gellrich plotted η_10_ against surface roughness factor for various Ni_*x*_Mo_*y*_ and Ni electrodes, as shown in Figure 23. The data clearly show an improvement of the overpotential from alloying Mo with Ni, even for high surface area Ni catalysts.^442^

Doping Ni_*x*_Mo_*y*_ materials is another route toward lower overpotentials. Zhang et al. reacted NiMo with nitrogen using a N_2_ plasma, a technique that facilitated rapid, low-temperature manipulation of the d-band and significantly increased the catalyst’s surface area. This was also proposed to improve mass transport, the catalyst’s adhesion to the substrate, and its conductivity.^443^ Xiong et al. demonstrated that NiMoO_4_ and MoO_2_ from a hydrothermal reaction subsequently doped with phosphorus increased the electrochemical surface area and reduced the potential of the electrode compared to the same electrode without P-doping.^444^

An alternative approach to improve the apparent electrode overpotential of a NiMo-treated electrode has demonstrated by Patil et al., who improved the catalyst layer’s conductivity by incorporating carbon black, up to 50 wt %, amplifying both the geometric and mass activities of the NiMo electrode for HER 5-fold.^445^ Their results suggests that a promising avenue for enhancing Ni_*x*_Mo_*y*_ performance could be to increase the conductivity of the catalyst layer.

**Figure 24:**
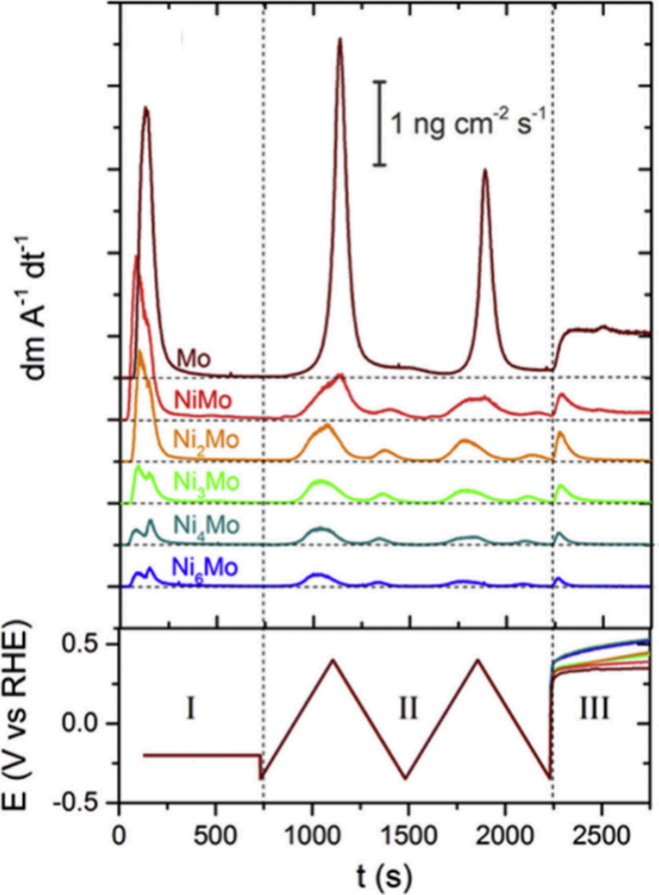
Mo dissolution from different Ni_*x*_Mo_*y*_ samples in 0.05 M NaOH measured using a scanning flow cell coupled to an ICP-MS. Below the dissolution graphs, the operational pattern is shown. A constant operation step at −200 mV vs RHE is followed by two triangular wave cyclic voltammetry measurements at 2 mV/s and finally the open circuit potential is measured for 500 s. All samples show Mo leaching. Reproduced with permission from ref (147). Copyright 2017 Elsevier.

The Pourbaix diagram for molybdenum (Figure 20) clearly shows that pure molybdenum lacks stability under highly alkaline conditions (specifically, pH 13 and above) for the HER. While alloying with nickel offers some degree of stabilization, computational analyses reveal that this stabilizing effect is relatively modest, amounting to only a small shift of 0.12 eV. The same calculations indicate that Ni_4_Mo lacks thermodynamic stability above −100 mV versus RHE.^152^ Schalenbach et al. conducted pivotal experimental work on the stability of Mo, NiMo, Ni_2_Mo, Ni_3_Mo, Ni_4_Mo, and Ni_6_Mo, using in-line scanning flow-cell ICP-MS setup.^147^ They studied the catalysts in two distinct potential windows; −300 mV vs RHE and up to +400 mV vs RHE finding that Mo begins to leach above −150 mV vs RHE at room temperature (Figure 24). These experiments demonstrate that Ni_*x*_Mo_*y*_ catalysts will leach during intermittent operation, if the electrodes go to OCP, unless preventative measures are deployed.

Despite the lack of stability for Ni_*x*_Mo_*y*_ catalysts above −150 mV vs RHE (Figure 24), studies have demonstrated that improved overpotentials are observed and sustained even during intermittent operation, which may seem to contradict the stability measurements. However, where Schalenbach et al. focused on leaching during an experiment with high control of variable, the experiments claiming stability are based in the continued low overpotentials, without quantification of dissolved products or electrochemically active surface area. Nevertheless, the very long experiments show continued activity toward HER in industrially relevant test conditions and -setups. For example, in a study by Mahmood et al. in 1984, Ni_*x*_Mo_*y*_ dip-coated electrodes were evaluated in terms of their resistance to electrolyzer stack off-periods resembling electricity blackouts and scheduled maintenance.^430^ The overpotential was measured over an 1100-h chronopotentiometry test, with a one-, two-, and three-day off-period during the experiment. During the off-periods, the anode and cathode terminals were short-circuited, which likely induced reverse current flow. Their data concluded enhanced activity was sustained even after long periods in the dissolving potential regime. In another study, Raj et al. conducted accelerated degradation tests, which involved running multiple slow CVs at 0.5 mV s^–1^ between −100 mV and 200 mV vs RHE, and found sustained activity of the NiMo samples.^446^ A noteworthy experiment in 1998 involved operating a model A-WE electrolyzer for 15,000 h under various operation patterns, using Mo-doped Raney Ni cathodes. Interestingly, the total cell voltage decreased–likely due to leaching or uptake of metal impurities–over this duration, suggesting that the functional stability of NiMo may be sufficient for specific application schemes.^447^

**Figure 25:**
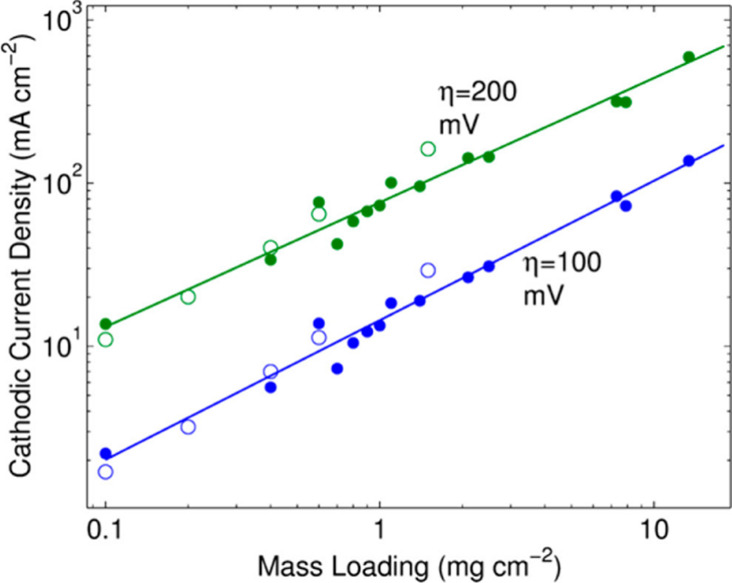
Current density at −100 mV and −200 mV vs RHE for Ti substrates coated with a range of mass loadings of Ni–Mo nanopowder in 2 M KOH. Open and closed circles represent data from two different sets of electrodes. Reproduced with permission from ref (440). Copyright 2012 American Chemical Society.

Importantly, the majority of published studies base their assessment of catalyst stability on the measured overpotential or cell voltage over time, and if these stay constant or improve, the Ni_*x*_Mo_*y*_ electrodes are declared stable. Unfortunately, this is insufficient evidence of catalyst stability, although it does provide some merit to the discussion on the possibility of implementing Ni_*x*_Mo_*y*_ for industrial use. Despite the instability of Ni_*x*_Mo_*y*_ subjected to potentials above −150 mV vs RHE, it can still be feasible to create electrodes with sufficiently high loadings to allow for long-term stability. Higher loadings have the added benefit of likely lowering the initial overpotential of the catalyst, as shown in Figure 25.^440^

Despite the significant interest in Ni_*x*_Mo_*y*_, controversy remains regarding the intrinsic activity of this catalyst family. The electrochemical stability of Ni_*x*_Mo_*y*_ electrodes also remains undeniably flawed, and the continuously record-breaking overpotentials are predominantly based on enhanced electrode surface areas and higher catalyst loadings. However, Ni_*x*_Mo_*y*_ compounds do consistently outperform other high-surface area Ni electrodes, excelling in activity and operational longevity.^83^ For continued Ni_*x*_Mo_*y*_ electrode development, overpotential reductions may be achieved by improving the catalyst layer’s conductivity. Furthermore, a clearer understanding of the relationship between synthesis parameters, loading, and electrode lifetime is needed.

#### 3.4.2 Phosphides

As discussed previously (Section 3.3.2.), the field of transition metal phosphides for the HER began with studies of phosphides in alkaline electrolyte. Inspired by Budniok’s work on NiP and CoP catalysts in 5 M KOH for isobutyl alcohol oxidation,^448^ Paseka in 1995,^449^ and Shervedani and Lasia in 1997,^379^ synthesized metal phosphides as an alternative to the well-established NiS catalysts already in use for alkaline HER. More than a decade later, Liu and Rodriguez’s DFT study reignited the interest in the field,^380^ although their findings better apply to acidic electrolytes as is also described in Section 3.3.2.^428^

**Figure 26:**
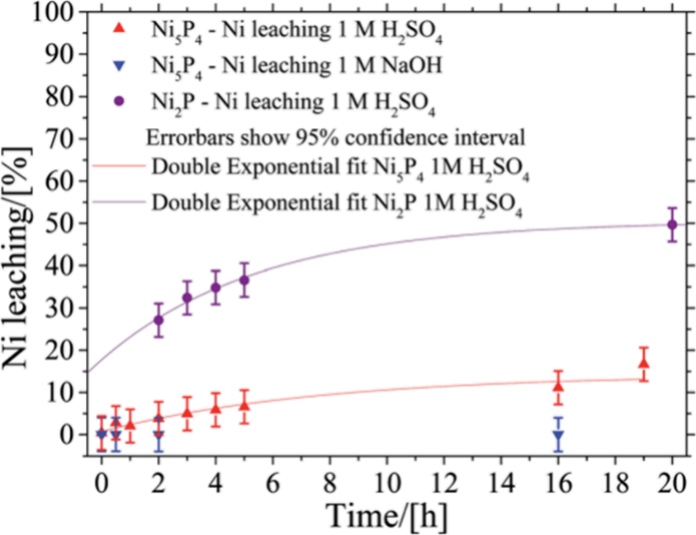
Leached Ni during chronopotentiometric analyses of Ni_2_P in 1 M H_2_SO_4_ and Ni_5_P_4_ in 1 M H_2_SO_4_ and 1 M NaOH. The measurement shows that Ni does not leach from the Ni_5_P_4_ in alkaline solutions. Reproduced with permission from ref (386). Copyright 2015 Royal Society of Chemistry.

Popczun et al. verified the predicted high HER activity of Ni_2_P in both acidic and alkaline electrolytes but also noted that the catalyst degraded rapidly in 1 M KOH.^381^ Feng and his colleagues later corroborated the issue with phosphide stability in alkaline electrolytes.^450^ They conducted a 48-h chronoamperometry experiment and observed that the current density of the Ni_2_P catalysts halved. Other researchers have reported semistable CP data on Ni_2_P and NiP_2_ electrodes for alkaline HER despite the early discouraging results.^451,452^ For example, Laursen et al. reported that the η_10_ remained between 24–28 mV vs RHE for 16 h for the Ni_5_P_4_ catalyst during a chronopotentiometry measurement in 1 M NaOH. Moreover, *ex situ* ICP measurements, as shown in Figure 26, showed no Ni-dissolution, even with a substantial catalyst loading of 177 mg cm^–2^.^386^ These stability findings are supported by other research groups.^453,454^ Ledendecker et al. also investigated the stability of Ni_5_P_4_ but conversely find a decline in activity (of almost 60%) during a 20-h constant potential measurement.^455^

The research into transition metal phosphides for alkaline electrolytes has since then mainly focused on Ni, Mo, and Co and combinations thereof. A range of compositions have been tested, most of which have highlighted severe stability issues when assessed with CP and CA experiments. Some of the most notable examples are highlighted below.

MoP was studied by Xiao et al., where, during a 40-h chronopotentiometry experiment, they observed a 60% reduction in activity.^389^ In contrast, a MoP/Ni_2_P composite on nickel foam reported by Du et al. demonstrated a greater level of stability, with only a 9% decline observed over a 24-h CP measurement.^456^

For CoP, one study showed >66% activity decay over 100 h, evidenced by CP and Tafel slopes.^457^ In another dedicated and thorough investigation into the stability of CoP, researchers found that phosphorus tends to leach more readily than cobalt, leading to the transformation of the CoP phase into Co(OH)_2,_ resulting in an increased overpotential for the HER.^135^

Bimetallic phosphide compositions have also been tested for HER properties in alkaline conditions. Guan et al. investigated Mo-doped CoP prepared through a reaction of Co-based metal–organic framework (MOF) with Na_2_MoO_4_ salt via a wet-synthesis method followed by annealing in an inert atmosphere.^458^ This process yielded a high-surface area catalyst, demonstrating semistable cathode operation with a 3% difference between the initial and final current densities in a 20-h chronoamperometry measurement. High-surface area NiCoP was tested in 2019 by Pan et al.^298^ As mentioned in Section 3.3.2, they found that it exhibited good stability in 0.5 M H_2_SO_4_, However, in 1 M KOH, the catalyst’s activity dropped from 13 mA cm^–2^ at η_100_ to 5 mA cm^–2^ within 21 h. Liang et al. reported similar results in their study on NiCoP. Initially, they reported a η_10_ of 32 mV, but after a 24-h CP measurement, the η_10_ increased to 75 mV. This activity drop represents more than a 10-fold activity drop considering their tafel slope of 37 mV dec^–1^.^459^ Co_0.59_Fe_0.41_P nanocubes, while demonstrating improved stability, still exhibited an activity drop of 25–30% during a 16-h chronoamperometry measurement.^460^

Man et al. conducted experimental and theoretical investigations into doping iron, cobalt, manganese, and molybdenum into a Ni_2_P hexagonal close-packed crystal lattice. Given each metal has a unique d-band properties, this variance enabled the modulation of a range of the final catalysts’ characteristics, such as η_10_, Tafel slope, and mass activity. Co-doped Ni_2_P was predicted to be the most active species for AWE applications at high current densities and to have the highest alloying stabilization energy of the calculated compositions. Mo-doped Ni_2_P was predicted to the most active catalyst at low current densities, which was confirmed experimentally.^461^ As stated in other sections of this review, it is crucial to note that electrochemical methods such as CA, CP, and CV serve as the most effective initial method for evaluating the stability of a catalyst. However, best practice always requires quantification of the catalyst loading and utilizing ICP quantification to determine the levels of dissolved elements during stability tests. Such data provides the most reliable means for comparing various research studies and offer the most accurate evaluation of a catalyst’s overall lifespan in a constant operation scenario.

In summary, the majority of transition metal phosphides for alkaline HER are active, but not stable. It is interesting to note that compared to acidic electrolyte, under alkaline conditions intrinsic activity metrics (e.g., TOF) are less commonly reported. We recommend that the wider community change this, to ensure catalyst loading and intrinsic activity metrics are always reported. Based on the electrochemical data, the most promising transition metal phosphides in alkaline electrolyte appears to be Ni_5_P_4_. Other Ni_*x*_P_*y*_ phases may prove alternative electrocatalysts candidates, while more research into MoCoP may also prove fruitful.

## 4 Outlook and Concluding Thoughts

Developing precious metal free HER catalyst has received significant attention from the academic community over the past decade. While there exist many different promising electrolyzer designs, this review focuses on precious metal free catalysts for low temperature water electrolyzers with potential relevance to the various commercial (PEM-WE and A-WE) and precommercial (AEM-WE) electrolyzer technologies. Accordingly, we focus on catalysts characterized in either acidic or alkaline electrolytes.

Various catalyst performance metrics are commonly used to assess and compare catalyst activity and stability for the HER. We find that there have been numerous best practices, editorials and perspectives published for half-cell 3-electrode measurements for the HER, with guidance published on many experimental factors ranging from the choice of electrochemical cell (glass, polymeric etc.), electrolyte, electrode selection (e.g., reference and counter electrodes), cell configurations and electrochemical measurement protocols (e.g., *iR* correction, electrochemical stability measurements). We note that papers are regularly published which do not follow these recommended best-practices. We highly recommend that the community reconsider their experimental design to align with these recommendations. Activity is commonly reported through geometric activity metrics (e.g., overpotential required for a catalyst to generate 10 mA cm^–2^_geo_). We note that throughout the HER literature, the mass loading of the catalyst on the working electrode is often not measured or reported properly, rendering the work highly speculative with regards to assessing differences between catalyst electrodes. We strongly recommend that the field makes every effort to assess catalyst loadings and report HER catalysts with electrochemical surface area normalized currents, mass activities and turnover frequencies. Looking forward, the intrinsic activities of HER catalysts (in one form or another) should become the central metric for comparing precious metal free catalysts.

Beyond assessing catalyst activity, electrocatalyst stability is also essential for commercial deployment. Given the numerous mechanisms by which an HER catalyst can degrade, various electrochemical measurements (typically for a few hours only) are often deployed. Such studies are typically insufficient yet frequently lead to claims of a “highly stable” catalyst. When we consider the aspects of stability, it becomes clear that there is not a one-size-fits-all approach that can describe all scenarios, from RDE-scale measurements to electrolyzer stack applications. Therefore, the type of stability studies conducted, and resultant stability claims should be clearly labeled and put into context with the literature to better clarify the prudent conclusions arising from the selected measurement type. Furthermore, given the absence of literature studies which systematically and quantitively compare the various techniques used to probe stability (e.g., CP, CA, LSVs, etc.) across a series of catalysts the community should consider undertaking such studies to enable recommendations of which technique are superior for describing durability.

Recently, some stability measurements have been coupled to ICP measurements. ICP enables quantification of catalyst dissolution and is commonly deployed through *ex situ* measurements of the electrolyte. While *in situ* measurements require complex and expensive dedicated setups, when designed effectively, they can offer significant and critical insight regarding catalyst stability. From such studies, it is clear that precious metal free HER catalysts are particularly prone to dissolution at open circuit voltage, rather than while under reducing potentials (turning over the HER). To reliably examine catalyst dissolution alone, however, we need to take into consideration the factors that may influence the catalyst dissolution measurement. The factors range from catalyst to ionomer ratios, the introduction of catalysts into the electrolyte under potential control, and the electrolyte volume used in the electrochemical cell. Furthermore, the electrochemical cell design deployed for ICP dissolution studies requires careful attention to ensure it can mimic conditions in commercial electrolyzers. For this purpose, a systematic benchmarking exercise to quantify the effect of each of these experimental factors on catalyst activity and catalyst dissolution would be helpful. In summary, assessing an electrocatalyst stability is a complex and challenging undertaking, but efforts must be made to enable more insightful comparisons between different catalysts.

Throughout the precious metal free HER literature, we frequently encounter reports that do not follow most up to date published best practices with regards to the electrochemical setup. We encourage the community to periodically review their standard operating protocols to incorporate the latest adjustments within the field. For example, usage of Pt counter electrodes was ubiquitous early on. The observation that Pt from a counter electrode can migrate to the working electrode led to separation of counter and working electrode compartments and eventually to additionally replace the counter electrodes with graphitic carbons. Specific recommendations are now clearly rationalized for nearly every component of the 3-electrode electrochemical measurements for HER, covering reference electrodes, electrolyte choice, cell design etc.

Across the precious metal free HER catalysis literature, the overwhelming majority of HER studies are tested in a 3-electrode lab-scale half-cell configuration, which enables a relatively simple, rapid, convenient and cost-effective platform for the study of the fundamental properties of HER catalysts. Device testing (e.g., MEAs or A-WE), however, represents a critical experimental platform to enable assessment of the translatability of developed catalysts into electrolyzer technologies. The low rates of translation from half-cell to device is understandable, as the industrially relevant cell environment is notably harsher, often featuring higher electrolyte concentrations, higher current measurements, elevated temperatures, and increased gas pressures. Consequently, it is experimentally and financially challenging to emulate these conditions in academic settings, necessitating certain simplifications and compromises. While we do not consider it possible (or appropriate) for all research groups to translate catalysts into working 2-electrode device testing, a clear recommendation is that the research community who do work with such devices standardize their testing protocols as far as possible. For example, during MEA investigations aiming to understand the translatability of HER catalysts into devices, where possible, all components beyond the cathode are purchased as half MEAs from commercial suppliers. Such practices will facilitate a true comparison between different HER catalysts, rather than differences owing to membrane thickness, catalyst deposition methods, or ink formulations etc from other components than cathode catalyst layers. Additionally, there are numerous critical investigations yet to be undertaken, even for the most frequently reported HER catalysts such as MoS_2_, Mo_2_C, and CoP, which would enable assessment of the translatability of precious metal free catalyst to device deployment. For example, studies investigating the role of catalyst loadings, ink formulations, deposition methods, etc. on the performance of an electrolyzer with PGM free HER catalyst as the cathode electrode.

Numerous different catalysts and catalyst structures have been synthesized and characterized for the HER. The structures were derived via various design strategies including nanostructuring, binder-free or self-supported architectures, catalyst–supports, spatial confinement, encapsulated catalysts, single atom catalysts, heterostructured catalysts, and defect engineering. We conclude that the primary objective of such catalyst designs is to boost the catalyst performance through enhancing one of the following: the density of active sites on the catalyst surface, the intrinsic activity of the catalyst, catalyst stability, and/or through improvements in the catalyst (or catalyst layer) conductivity. We recommend that the community should seek careful design of control samples when seeking to claim enhanced HER activity or stability to ensure such catalyst designs are reported appropriately and accurately.

With regards to HER material choice, we highlight that nearly the entire periodic table has been explored in some capacity as an electrocatalyst for the HER. To assess the progress within the field, we focus our review on transition metal sulfides, phosphides and carbides as precious metal free HER catalysts in acidic electrolyte. In alkaline electrolyte we focus on Ni_*x*_Mo_*y*_ and transition metal phosphides. To explore activity trends across precious metal free HER catalysts, we collated a large set of published activity data for the sulfides, phosphides and carbides in acidic electrolyte. It is clear from these data that some materials can reach high geometric activities. However, when activity is normalized by catalyst loading (mass activities) the vast majority of geometric activity improvements are evidently due to increased catalyst loading, rather than any improvement to the intrinsic activity of the precious metal free catalysts. Indeed, when mass activities and turnover frequencies are compared to platinum-base catalysts, it is evident that precious metal free HER catalysts are significantly (3–4 orders of magnitude) inferior. We therefore recommend that future 3-electrode lab-scale testing emphasize mass activity or turnover frequency to benchmark the catalyst performance, rather than seeking higher geometric activities. Furthermore, across the HER literature (and more broadly electrocatalysis), we recommend that activity and stability metrics should include error bars, with average overpotentials with standard deviations used to compare series of catalysts.

Beyond the electrolyzer technologies discussed in this review (A-WE, PEM-WE, and AEM-WE) there remain a wealth of potential alternate applications and opportunities for the precious metal free HER catalysis community. For example, SO-WE technology is on the verge of commercialization. Also undoubtably, commercial electrolyzers of the future may have entirely different cell designs to those deployed today, expanding the operating window with regards to pH. Furthermore, recent literature has highlighted interest in seawater electrolysis, and unprocessed (nondeionized) water feeds may also become important in electrolysis. Hence, the need to quantify, develop and benchmark HER catalyst performance with typical water impurities may indeed prove critical. There are also potential opportunities for the HER beyond water splitting, e.g., through tandem catalysis for electrochemical hydrogenation of CO_2_ or coupling to an electro-organic oxidation reaction. Looking forward, we encourage continued innovations beyond the scope set out in this review and encourage the community to seek alternative materials and designs that may not be suitable for what is considered state-of-the-art technology currently. However, we remind the community to also ensure that they provide an honest assessment of the activity and stability of the catalyst to enable true comparison to state-of-the-art catalysts; this is essential whether the work is motivated by developing fundamental understanding, new electrolyzer designs, utilizing seawater or developing alternative applications of HER catalysts beyond the water splitting.

## Supporting Information Available

Catalyst names, activity metrics, mass loadings, and other data, associated references from where the data was collected to generate Figure 18 and Figure 19 (PDF)
