# Supplementary material for: Precious Metal Free Hydrogen Evolution Catalyst Design and Application
Source: Chem Rev. 2024 Apr 25;124(9):5617–67. doi: 10.1021/acs.chemrev.3c00712 (PMC11082907; doi:10.1021/acs.chemrev.3c00712)
Supplement: Supplementary file 1 — cr3c00712_si_001.pdf [file cr3c00712_si_001.pdf]

Supporting Information

Precious Metal Free Hydrogen Evolution Catalyst Design and Application

Anders A. Feidenhans'l [1], Yagya N Regmi [2,3], Chao Wei [1], Dong Xia [2,3], Jakob Kibsgaard [1], Laurie A. King[2,3*]

[1]Department of Physics, Technical University of Denmark, 2800 Kongens Lyngby, Denmark

[2]Faculty of Science and Engineering, Manchester Metropolitan University, Manchester M1 5GD, UK

[3]Manchester Fuel Cell Innovation Centre, Manchester Metropolitan University, Manchester M1 5GD, UK

*l.king@mmu.ac.uk

Table of contents

Table S1. Transition metal phosphide hydrogen evolution reaction electrocatalyst activities.

Table S2. Transition metal sulfide hydrogen evolution reaction electrocatalyst activities.

Table S3. Transition metal carbide hydrogen evolution reaction electrocatalyst activities.

Table S4. Platinum-based hydrogen evolution reaction electrocatalyst activities.

The data provided in this supporting information file was used to prepare Figures 18 and 19 in the main text.

Table S1. Transition metal phosphide hydrogen evolution reaction electrocatalyst activities.

Date of publication	Catalyst	Electrolyte	Loading [mg cm^{-2}]	Overpotential to achieve TOF [mV] vs RHE	TOF [H_2 s^{-1} $site^{-1}$]	Overpotential to achieve geometric current density and mass activity [mV] vs RHE	Geometric current density [mA cm^{-2}]	Mass activity [A mg^{-1}]	DOI
03/06/2013	Nanoporous FeP nanosheets	0.5 M H2SO4	0.28			237	10	3.57E-02	https://doi.org/10.1016/j.electacta.2019.134798
13/06/2013	Nanostructured Ni2P/Ti	0.5 M H2SO4	1.00	100.00	1.50E-02	130	20	2.00E-02	https://doi.org/10.1021/ja403440e
13/06/2013	Nanostructured Ni2P/Ti	0.5 M H2SO4	1.00	200.00	5.00E-01	180	100	1.00E-01	https://doi.org/10.1021/ja403440e
11/04/2014	Nanostructured CoP	0.5 M H2SO4	0.90	100.00	4.20E-02	95	20	2.22E-02	https://doi.org/10.1002/anie.201402646
11/04/2014	Nanostructured CoP	0.5 M H2SO4	2.00			85	20	1.00E-02	https://doi.org/10.1002/anie.201402646
15/05/2014	Cobalt Phosphide Nanowire Arrays	0.5 M H2SO4	0.92	75.00	7.25E-01	67	10	1.09E-02	https://doi.org/10.1021/ja503372r
15/05/2014	Cobalt Phosphide Nanowire Arrays	0.5 M H2SO4	0.92	240.00	4.00E+00	100	20	2.17E-02	https://doi.org/10.1021/ja503372r
15/05/2014	Cobalt Phosphide Nanowire Arrays	0.5 M H2SO4	0.92			204	100	1.09E-01	https://doi.org/10.1021/ja503372r
21/07/2014	CoP nanotube	0.5 M H2SO4	0.20	106.00	1.00E+00	72	2	1.00E-02	https://doi.org/10.1039/C4TA02368D

21/07/2014	CoP nanotube	0.5 M H2SO4	0.20			129	10	5.00E-02	https://doi.org/10.1039/C4TA02368D
21/07/2014	CoP nanoparticles	0.5 M H2SO4	0.20	172.00	1.00E+00	200	2	1.00E-02	https://doi.org/10.1039/C4TA02368D
21/07/2014	CoP nanoparticles	0.5 M H2SO4	0.20			297	10	5.00E-02	https://doi.org/10.1039/C4TA02368D
23/07/2014	CoP	0.5 M H2SO4	2.00			90	10		https://doi.org/10.1021/acscatal.8b04291
23/07/2014	CoP	0.5 M H2SO4	2.00			146	100		https://doi.org/10.1021/cm501273s
01/08/2014	Ni2P nanoparticle films	1.0 M H2SO4	2.00	205.00	7.25E-01	120	10	5.00E-03	https://doi.org/10.1039/C4NR03037K
01/08/2014	Ni2P nanoparticle films	1.0 M H2SO4	2.00			138	20	1.00E-02	https://doi.org/10.1039/C4NR03037K
01/08/2014	Ni2P nanoparticle films	1.0 M H2SO4	2.00			188	100	5.00E-02	https://doi.org/10.1039/C4NR03037K
11/08/2014	MoP	0.5 M H2SO4	0.07	100.00	4.80E-02	246	10	1.41E-01	https://doi.org/10.1039/C4CC05936K
11/08/2014	Ni2P	0.5 M H2SO4	0.07	100.00	2.50E-02	346	10	1.41E-01	https://doi.org/10.1039/C4CC05936K
20/08/2014	FeP Nanorod array/Ti	0.5 M H2SO4	0.60	85.00	2.58E+00	85	10	1.67E-02	https://doi.org/10.1039/C4TA03638G
20/08/2014	FeP Nanorod array/Ti	0.5 M H2SO4	0.60			183	240	4.00E-01	https://doi.org/10.1039/C4TA03638G
26/08/2014	Co2P nanorod	0.5 M H2SO4	1.00	143.00	7.25E-01	167	20	2.00E-02	https://doi.org/10.1016/j.nanoen.2014.08.013
04/09/2014	Amorphous W doped nickel P	0.5 M H2SO4	1.50	100.00	7.25E-01	110	20	1.33E-02	https://doi.org/10.1039/C4TA04434G
04/09/2014	Amorphous W doped nickel P	0.5 M H2SO4	1.50			180	100	6.67E-02	https://doi.org/10.1039/C4TA04434G

18/09/2014	NiP2nanosheet arrays	0.5 M H2SO4	4.30			75	10	2.33E-03	https://doi.org/10.1039/C5TA02128F
18/09/2014	NiP2nanosheet arrays	0.5 M H2SO4	4.30			99	20	4.65E-03	https://doi.org/10.1039/C4NR04866K
18/09/2014	NiP2nanosheet arrays	0.5 M H2SO4	4.30			204	100	2.33E-02	https://doi.org/10.1039/C4NR04866K
01/10/2014	Nanostructured FeP/Ti	0.5 M H2SO4	1.00	100.00	2.77E-01	50	10	1.00E-02	https://doi.org/10.1021/nn5048553
01/10/2014	Nanostructured FeP/Ti	0.5 M H2SO4	1.00			61	20	2.00E-02	https://doi.org/10.1021/nn5048553
17/10/2014	FeP Nanorod Array	0.5 M H2SO4	1.50			58	10	6.67E-03	https://doi.org/10.1039/C4TA04867A
30/10/2014	MoP|S	0.5 M H2SO4	1.00	100.00	1.20E-01	86	10	1.00E-02	https://doi.org/10.1002/anie.201408222
30/10/2014	MoP|S	0.5 M H2SO4	1.00	150.00	7.50E-01			0.00E+00	https://doi.org/10.1002/anie.201408222
30/10/2014	MoP|S	0.5 M H2SO4	3.00			64	10	3.33E-03	https://doi.org/10.1002/anie.201408222
30/10/2014	MoP|S	0.5 M H2SO4	3.00			78	20	6.67E-03	https://doi.org/10.1002/anie.201408222
30/10/2014	MoP|S	0.5 M H2SO4	3.00			120	100	3.33E-02	https://doi.org/10.1002/anie.201408222
30/10/2014	MoP in H2 anneal	0.5 M H2SO4	1.00			90	10	1.00E-02	https://doi.org/10.1002/anie.201408222
30/10/2014	MoP	0.5 M H2SO4	1.00	100.00	2.40E-02	117	10	1.00E-02	https://doi.org/10.1002/anie.201408222
30/10/2014	MoP	0.5 M H2SO4	1.00	150.00	1.90E-01	180	100	1.00E-01	https://doi.org/10.1002/anie.201408222
04/11/2014	Ni12P5 NC	0.5 M H2SO4	1.99			208	10	5.03E-03	https://doi.org/10.1039/C4NR04866K
04/11/2014	Ni2P NC	0.5 M H2SO4	1.99			137	10	5.03E-03	https://doi.org/10.1039/C4TA04867A

04/11/2014	Ni5P4 NC	0.5 M H2SO4	1.99			118	10	5.03E-03	https://doi.org/10.1039/C4TA04867A
09/01/2015	Ni5P4 (pellet)	1.0 M H2SO4	176.56	100.00	3.50E+00	23	10	5.66E-05	https://doi.org/10.1039/C4EE02940B
09/01/2015	Ni5P4 (pellet)	1.0 M H2SO4	176.56	200.00	9.80E+00	62	100	5.66E-04	https://doi.org/10.1039/C4EE02940B
09/01/2015	Ni2P (pellet)	1.0 M H2SO4	177.27	100.00	1.50E-02	42	10	5.64E-05	https://doi.org/10.1039/C4EE02940B
09/01/2015	Ni2P (pellet)	1.0 M H2SO4	177.27	200.00		101	100	5.64E-04	https://doi.org/10.1039/C4EE02940B
12/01/2015	CoP-OMC	0.5 M H2SO4	0.29	200.00	2.70E+00	112	10	3.51E-02	https://doi.org/10.1039/C4TA06630H
12/01/2015	CoP NPs	0.5 M H2SO4	0.29	200.00	1.70E+00	212	10	3.51E-02	https://doi.org/10.1039/C4TA06630H
16/01/2015	Highly Branched CoP/Ti	0.5 M H2SO4	1.00	100.00	1.90E-02	117	20	2.00E-02	https://doi.org/10.1039/C4TA06642A
27/03/2015	Ni2P nanowires	1.0 M H2SO4	1.42			133	10	7.04E-03	https://doi.org/10.1002/adma.201800140
20/04/2015	Ni2P/CNT	0.5 M H2SO4	0.18			98	2	1.09E-02	https://doi.org/10.1021/acs.nanolett.6b02203
20/04/2015	Ni2P/CNT	0.5 M H2SO4	0.18			124	10	5.43E-02	https://doi.org/10.1039/C5TA02128F
20/04/2015	Ni12P5/CNT	0.5 M H2SO4	0.18			240	10	5.43E-02	https://doi.org/10.1039/C5TA02128F
05/02/2016	CoP/NCNTs	0.5 M H2SO4	0.20			79	10	5.03E-02	https://doi.org/10.1039/C5CP01065A
05/02/2016	CoP/NCNTs	0.5 M H2SO4	0.20			99	20	1.01E-01	https://doi.org/10.1039/C6TA00575F
05/02/2016	Co2P/NCNTs	0.5 M H2SO4	0.20			171	20	1.01E-01	https://doi.org/10.1039/C6TA00575F

07/06/ 2016	Co2P@NPG	0.5 M H2SO4	0.50			45	1	2.00E-03	https://doi.org/10.1002/smll.201602873
07/06/ 2016	Co2P@NPG	0.5 M H2SO4	0.50			103	10	2.00E-02	https://doi.org/10.1021/acs.nanolett.6b02203
07/06/ 2016	Co2P@NPG	0.5 M H2SO4	0.50			128	20	4.00E-02	https://doi.org/10.1021/acs.nanolett.6b02203
07/06/ 2016	Co2P@NPG	0.5 M H2SO4	0.50			220	100	2.00E-01	https://doi.org/10.1021/acs.nanolett.6b02203
07/07/ 2016	MoP@PC	0.5 M H2SO4	0.41			153	10	2.44E-02	https://doi.org/10.1021/acscatal.7b00555
01/02/ 2017	CoP–CNTs	0.5 M H2SO4	0.28			139	10	3.53E-02	https://doi.org/10.1039/C6TA00575F
29/03/ 2017	MoP/SN nanoparticles	0.5 M H2SO4	90.50			57	1	1.10E-05	https://doi.org/10.1021/acscatal.7b00555
29/03/ 2017	MoP/SN nanoparticles	0.5 M H2SO4	0.50			104	10	2.00E-02	https://doi.org/10.1021/acscatal.7b00555
17/12/ 2017	Ni2P@NPCNFs	0.5 M H2SO4	0.34			63	10	2.97E-02	https://doi.org/10.1002/anie.201710150
17/12/ 2017	Ni2P@NPCNFs	0.5 M H2SO4	0.34			85	20	5.93E-02	https://doi.org/10.1002/anie.201710150
17/12/ 2017	Ni2P@NPCNFs	0.5 M H2SO4	0.34			173	100	2.97E-01	https://doi.org/10.1002/anie.201710150
26/12/ 2017	Co2P@NPC/CC	0.5 M H2SO4	5.00			116	10	2.00E-03	https://doi.org/10.1039/C7NR08148K
26/12/ 2017	Co2P@NPC/CC	0.5 M H2SO4	5.00			134	20	4.00E-03	https://doi.org/10.1039/C7NR08148K
26/12/ 2017	Co2P@NPC/CC	0.5 M H2SO4	5.00			154	50	1.00E-02	https://doi.org/10.1039/C7NR08148K
03/04/ 2018	PANI/CoP HNWs-CF	0.5 M H2SO4	0.80			57	10	1.25E-02	https://doi.org/10.1021/jacs.7b12968
03/04/ 2018	PANI/CoP HNWs-CF	0.5 M H2SO4	0.80			82	20	2.50E-02	https://doi.org/10.1021/jacs.7b12968

03/04/ 2018	PANI/CoP HNWs-CF	0.5 M H2SO4	0.80			101	50	6.25E-02	https://doi.org/10.1021/jacs.7b12968
03/04/ 2018	PANI/CoP HNWs-CF	0.5 M H2SO4	0.80			122	100	1.25E-01	https://doi.org/10.1021/jacs.7b12968
16/05/ 2018	Co2P@CP	0.5 M H2SO4	3.20			120	10	3.13E-03	https://doi.org/10.1021/acsenergylett.8b00514
17/05/ 2018	CoP	0.5 M H2SO4	1.12	50.00	3.10E-03	85	10	8.93E-03	https://doi.org/10.1021/cm501273s
17/05/ 2018	N-doped CoP	0.5 M H2SO4	1.08	50.00	1.99E-02	42	10	9.26E-03	https://doi.org/10.1002/adma.201800140
05/07/ 2018	MoP@HCC	0.5 M H2SO4	0.26			129	10	3.85E-02	https://doi.org/10.1002/anie.201604315
08/11/ 2018	3D-NiCoP	0.5 M H2SO4				80	10		https://doi.org/10.1007/s12274-018-2226-2
19/12/ 2018	S-MoP NPL	0.5 M H2SO4				86	10		https://doi.org/10.1021/acscatal.8b04291
19/12/ 2018	S-MoP NPL	0.5 M H2SO4				104	20		https://doi.org/10.1021/acscatal.8b04291
19/12/ 2018	S-MoP NPL	0.5 M H2SO4				145	100		https://doi.org/10.1021/acscatal.8b04291
15/02/ 2019	Ni-doped FeP	0.5 M H2SO4	0.40			72	10	2.50E-02	https://doi.org/10.1126/sciadv.aav6009
15/02/ 2019	Ni-doped FeP	0.5 M H2SO4	0.40			89	20	5.00E-02	https://doi.org/10.1126/sciadv.aav6009
15/02/ 2019	Ni-doped FeP	0.5 M H2SO4	0.40			115	50	1.25E-01	https://doi.org/10.1126/sciadv.aav6009
15/02/ 2019	Ni-doped FeP	0.5 M H2SO4	0.40			138	100	2.50E-01	https://doi.org/10.1126/sciadv.aav6009
15/03/ 2019	N-Co2P/CC	0.5 M H2SO4	5.00			27	10	2.00E-03	https://doi.org/10.1021/acscatal.9b00407
20/03/ 2019	Ni5P4@Nickel hydr(oxy)oxide	0.5 M H2SO4				66	10		https://doi.org/10.1016/j.apcatb.2019.03.037

25/03/2019	CoP@PC-750	0.5 M H2SO4	1.00			72	10	1.00E-02	https://doi.org/10.1002/smll.201900550
15/04/2019	N-CoP/CC	0.5 M H2SO4	5.00			25	10	2.00E-03	https://doi.org/10.1016/j.apcatb.2019.04.038
02/09/2019	LC-WP	0.5 M H2SO4	0.21	200.00	3.60E-01	105	1	4.69E-03	https://doi.org/10.1016/j.apcatb.2019.118358
02/09/2019	LC-WP	0.5 M H2SO4	0.21			170	10	4.69E-02	https://doi.org/10.1016/j.electacta.2019.134798
02/09/2019	LC-WP	0.5 M H2SO4	0.21			300	81	3.78E-01	https://doi.org/10.1016/j.electacta.2019.134798
02/11/2019	MoP@NPCS	0.5 M H2SO4	0.25			113	10	4.00E-02	https://doi.org/10.1016/j.apcatb.2019.118352
08/11/2019	MoP@NC	0.5 M H2SO4	0.28			96	10	3.57E-02	https://doi.org/10.1039/C8NR04246B
25/04/2020	CP@NCNT)	0.5 M H2SO4	0.27			94	10	3.70E-02	https://doi.org/10.1016/j.jechem.2020.04.005
25/04/2020	CP@NCNT)	0.5 M H2SO4	0.27			174	50	1.85E-01	https://doi.org/10.1016/j.jechem.2020.04.005
06/10/2020	hierarchical porous Ni12P5-Ni2P	0.5 M H2SO4				83	10		https://doi.org/10.1016/j.apcatb.2020.119609
15/10/2020	NiCoP/NPC	0.5 M H2SO4				108	10		https://doi.org/10.1016/j.apcatb.2020.119635
17/11/2020	Co2P/Ni2P nanohybrid	0.5 M H2SO4				46	20		https://doi.org/10.1016/j.mtphys.2020.100314
08/02/2021	P-MoP/Mo2N	0.5 M H2SO4		100.00	6.50E-02	89	10		https://doi.org/10.1002/anie.202016102
24/02/2021	Ni-graphene-CNTs-Ni2P–CuP2 heterostructure	0.5 M H2SO4	11.25	100.00	1.00E+00	12	10	8.89E-04	https://doi.org/10.1021/acsnano.1c00647
24/02/2021	Ni-graphene-CNTs-Ni2P–	0.5 M H2SO4	11.25	142.00	2.00E+00	124	100	8.89E-03	https://doi.org/10.1021/acsnano.1c00647

	CuP2 heterostructure								
24/02/2021	Ni-graphene-CNTs-Ni2P–CuP2 heterostructure	0.5 M H2SO4	11.25	174.00	3.00E+00	174	200	1.78E-02	https://doi.org/10.1021/acsnano.1c00647
27/03/2021	CoP@N,S-3D-GN	0.5 M H2SO4	0.20			118	10	5.00E-02	https://doi.org/10.1016/j.electacta.2021.138262
27/03/2021	CoP@3D-GN	0.5 M H2SO4	0.20			198	10	5.00E-02	https://doi.org/10.1016/j.electacta.2021.138262

Table S2. Transition metal sulfide hydrogen evolution reaction electrocatalyst activities.

**Date of publication**	**Catalyst**	**Electrolyte**	**Loading [mg cm^{-2}]**	**Overpotential for TOF [mV] vs RHE**	**TOF [H_2 s^{-1} site^{-1}]**	**Overpotential to achieve geometric current density and mass activity [mV] vs RHE**	**Geometric current density [mA cm^{-2}]**	**Mass activity [A mg^{-1}]**	**DOI**
06/07/2007	UHV MoS2 - edges	0.5 M H2SO4	0.00	150	9.37E+00	150	0	3.30E+00	https://doi.org/10.1126/science.1141483
06/07/2007	UHV MoS2 - all sites	0.5 M H2SO4		150	2.70E+00				https://doi.org/10.1126/science.1141483
14/04/2011	Amorphous molybdenum sulfide	0.5 M H2SO4				242	10		https://doi.org/10.1039/C1SC00117E
21/04/2011	MoS2 RGO	0.5 M H2SO4	0.28	200	1.00E-01	200	33	1.18E-01	https://doi.org/10.1021/ja201269b
21/04/2011	MoS2 RGO	0.5 M H2SO4	0.28	250	3.00E-01	250	101	3.60E-01	https://doi.org/10.1021/ja201269b

14/09/ 2011	Core–shell MoO3–MoS2 Nanowires	0.5 M H2SO4				254	10		https://doi.org/10.1021/nl2020476
14/09/ 2011	Core–shell MoO3–MoS2 Nanowires	0.5 M H2SO4		272	4.00E+00	272	20		https://doi.org/10.1021/nl2020476
10/08/ 2012	Amorphous Molybdenum Sulfide	0.5 M H2SO4		200	3.00E-01	200	10		https://doi.org/10.1021/cs300451q
07/10/ 2012	DG MoS2	0.5 M H2SO4				206	10		https://doi.org/10.1038/nmat3439
12/02/ 2013	MoS2	0.5 M H2SO4	0.01	0	1.30E-02	300	1	7.06E-02	https://doi.org/10.1021/nl400258t
11/04/ 2013	MoSx/GP	0.5 M H2SO4				194	10		https://doi.org/10.1039/C3CC41945B
11/04/ 2013	MoS2/piranha GP	0.5 M H2SO4				175	6		https://doi.org/10.1039/C3CC41945B
11/04/ 2013	MoS2/piranha GP	0.5 M H2SO4				154	10		https://doi.org/10.1039/C3CC41945B
06/08/ 2013	Electro deposited MoS2+x	1.0 M H2SO4	0.02	200	7.60E-01	200	12	7.67E-01	https://doi.org/10.1021/cs400441u
06/08/ 2013	Electro deposited MoS2+x	1.0 M H2SO4	0.02	250	1.30E+01	250	196	1.31E+01	https://doi.org/10.1021/cs400441u
21/11/ 2013	1T basal plane	0.5 M H2SO4	0.05	150	1.55E+00	201	10	2.00E-01	https://doi.org/10.1021/nl403661s
21/11/ 2013	1T basal plane	0.5 M H2SO4	0.05	180	8.72E+00	217	20	4.00E-01	https://doi.org/10.1021/nl403661s
03/12/ 2013	Lithiated ALDMoS2t	0.5 M H2SO4	0.12			168	10	8.33E-02	https://doi.org/10.1073/pnas.1316792110

03/12/2013	Lithiated ALDMoS2t	0.5 M H2SO4	0.12			216	100	8.33E-01	https://doi.org/10.1073/pnas.1316792110
03/12/2013	lithiated MoS2on MPGC	0.5 M H2SO4	0.02			200	7	3.06E-01	https://doi.org/10.1073/pnas.1316792110
03/12/2013	lithiated MoS2on MPGC	0.5 M H2SO4	0.02			113	0	4.55E-03	https://doi.org/10.1073/pnas.1316792110
26/01/2014	Mo3S13|HOPG	0.5 M H2SO4	0.00	200	3.12E+00	200	0	2.58E+00	https://doi.org/10.1038/nchem.1853
26/01/2014	Mo3S13|HOPG	0.5 M H2SO4	0.00	250	2.15E+01	250	0	1.77E+01	https://doi.org/10.1038/nchem.1853
26/01/2014	Mo3S13|GP	0.5 M H2SO4	0.01	214	1.21E+00	214	10	1.00E+00	https://doi.org/10.1038/nchem.1853
26/01/2014	Mo3S13|GP	0.5 M H2SO4	0.02	196	6.08E-01	196	10	5.00E-01	https://doi.org/10.1038/nchem.1853
26/01/2014	Mo3S13|GP	0.5 M H2SO4	0.05	183	2.44E-01	183	10	2.00E-01	https://doi.org/10.1038/nchem.1853
26/01/2014	Mo3S13|GP	0.5 M H2SO4	0.10	176	1.21E-01	176	10	1.00E-01	https://doi.org/10.1038/nchem.1853
06/02/2014	MoSx /N-CNT	0.5 M H2SO4	0.10	200	3.50E+00	110	10	9.80E-02	https://doi.org/10.1021/nl404108a
09/04/2014	1T MoS2	0.5 M H2SO4	3.40			118	10	2.94E-03	https://doi.org/10.1021/nn500959v
09/04/2014	1T MoS2	0.5 M H2SO4	3.40			200	200	5.88E-02	https://doi.org/10.1021/nn500959v
08/10/2014	Mo3S13 anodized GP	0.5 M H2SO4				149	10		ttps://doi.org/10.1021/cs500923c
09/01/2015	ALD MoS2 film	0.5 M H2SO4		200	1.45E+00	200	1		https://doi.org/10.1021/la504162u
09/01/2015	ALD MoS2 film	0.5 M H2SO4		215	3.00E+00				https://doi.org/10.1021/la504162u
03/11/2015	1T@2H-MoS2	0.5 M H2SO4	0.32			64	10	3.13E-02	https://doi.org/10.1039/C5TA08520A

09/11/2015	SV-MoS2	0.5 M H2SO4		0	3.10E-01				https://doi.org/10.1038/nmat4465
09/11/2015	SV-MoS2	0.5 M H2SO4		50	9.88E-01	170	10		https://doi.org/10.1038/nmat4465
09/11/2015	SV-MoS2	0.5 M H2SO4		100	5.42E+00				https://doi.org/10.1038/nmat4465
09/11/2015	SV-MoS2	0.5 M H2SO4		150	1.79E+01				https://doi.org/10.1038/nmat4465
09/11/2015	V-MoS2	0.5 M H2SO4		50	2.78E-01	250	10		https://doi.org/10.1038/nmat4465
09/11/2015	V-MoS2	0.5 M H2SO4		100	9.03E-01				https://doi.org/10.1038/nmat4465
09/11/2015	V-MoS2	0.5 M H2SO4		150	3.32E+00				https://doi.org/10.1038/nmat4465
09/11/2015	S-MoS2	0.5 M H2SO4		237	1.01E+00				https://doi.org/10.1038/nmat4465
09/11/2015	S-MoS2	0.5 M H2SO4		297	5.00E+00				https://doi.org/10.1038/nmat4465
09/11/2015	MoS2	0.5 M H2SO4		248	1.00E+00				https://doi.org/10.1038/nmat4465
09/11/2015	MoS2	0.5 M H2SO4		316	5.01E+00				https://doi.org/10.1038/nmat4465
25/04/2016	MoS2/N-RGO-180	0.5 M H2SO4	0.14			56	10	7.14E-02	https://doi.org/10.1002/aenm.201600116
15/12/2016	MoS2 - edge sites	0.5 M H2SO4		0	7.50E+00				https://doi.org/10.1021/jacs.6b05940
15/12/2016	MoS2 - sulfur vacancies	0.5 M H2SO4		0	3.20E+00				https://doi.org/10.1021/jacs.6b05940
15/12/2016	MoS2 - grain boundaries	0.5 M H2SO4		0	1.00E-01				https://doi.org/10.1021/jacs.6b05940

15/12/2016	7% densities of sulfur vacancies	0.5 M H2SO4				163	10		https://doi.org/10.1021/jacs.6b05940
15/12/2016	10% densities of sulfur vacancies	0.5 M H2SO4				187	10		https://doi.org/10.1021/jacs.6b05940
15/12/2016	12% densities of sulfur vacancies	0.5 M H2SO4				261	10		https://doi.org/10.1021/jacs.6b05940
15/12/2016	14% densities of sulfur vacancies	0.5 M H2SO4				304	10		https://doi.org/10.1021/jacs.6b05940
15/12/2016	4% densities of sulfur vacancies	0.5 M H2SO4				325	10		https://doi.org/10.1021/jacs.6b05940
15/12/2016	2% densities of sulfur vacancies	0.5 M H2SO4				366	10		https://doi.org/10.1021/jacs.6b05940
02/03/2017	P-doped MoS2 (P3)	0.5 M H2SO4	0.32	100	1.40E+00	43	10	3.13E-02	https://doi.org/10.1021/acsenergylett.7b00111
07/03/2017	MoS2 nanowall	0.5 M H2SO4	0.88			95	10	1.14E-02	https://doi.org/10.1007/s12274-017-1421-x
07/03/2017	MoS2 nanowall	0.5 M H2SO4	0.88			300	311	3.53E-01	https://doi.org/10.1007/s12274-017-1421-x
12/04/2017	MoS2 foam	0.5 M H2SO4	0.50			210	10	2.00E-02	https://doi.org/10.1038/ncomms14430
12/04/2017	Co-MoS2 foam	0.5 M H2SO4	0.50			156	10	2.00E-02	https://doi.org/10.1038/ncomms14430
24/04/2017	MoS2/Ti3C2-MXene@C	0.5 M H2SO4	0.40			135	10		https://doi.org/10.1002/adma.201607017
08/05/2017	1TMoS2/SWNT	0.5 M H2SO4				108	10		https://doi.org/10.1021/acs.chemmater.7b00446

03/08/2017	NiS2/MoS2 HNW	0.5 M H2SO4	0.20			235	10	5.00E-02	https://doi.org/10.1021/acscatal.7b02225
18/01/2018	MoS2/HG	0.5 M H2SO4	0.13	220	7.80E+00	124	10	7.87E-02	https://doi.org/10.1021/acscatal.7b03316
07/02/2018	MoSx polymer brush composite	1.0 M H2SO4	0.04	200	1.30E+00	211	1	1.43E-02	https://doi.org/10.1021/acsami.7b16679
07/02/2018	MoSx polymer brush composite	1.0 M H2SO4	0.04	250	4.90E+00				https://doi.org/10.1021/acsami.7b16679
18/03/2018	Co-MoS2-0.5	0.5 M H2SO4	2.00			60	10	5.00E-03	https://doi.org/10.1039/C8CC00766G
20/03/2018	1T-MoS2	0.5 M H2SO4	0.29	200	2.50E-01	76	10	3.51E-02	https://doi.org/10.1007/s12274-018-2026-8
20/03/2018	1T-MoS2	0.5 M H2SO4	0.29			164	100	3.51E-01	https://doi.org/10.1007/s12274-018-2026-8
02/04/2018	1T'-MoS2	0.5 M H2SO4				400	607		https://doi.org/10.1038/s41557-018-0035-6
02/04/2018	2H-MoS2	0.5 M H2SO4				400	43		https://doi.org/10.1038/s41557-018-0035-6
02/04/2018	1T'-MoS2	0.5 M H2SO4				175	10		https://doi.org/10.1038/s41557-018-0035-6
02/04/2018	1T'-MoS2	0.5 M H2SO4				400	607		https://doi.org/10.1038/s41557-018-0035-6
17/01/2019	MoS2/Mo2C	0.5 M H2SO4	0.30			227	1000	3.33E+00	https://doi.org/10.1038/s41467-018-07792-9
23/01/2019	1T-MoS2	0.5 M H2SO4	1.70			151	10	5.88E-03	https://doi.org/10.1016/j.apcatb.2019.01.062
01/03/2019	L-MoS2	0.5 M H2SO4	0.20	250	7.67E+00	178	10	5.00E-02	https://doi.org/10.1021/acssuschemeng.8b06717
01/03/2019	P-MoS2	0.5 M H2SO4	0.20	250	4.19E-01	256	10	5.00E-02	https://doi.org/10.1021/acssuschemeng.8b06717

05/10/2019	V-doped MoS2	0.5 M H2SO4	0.50			194	10	2.00E-02	https://doi.org/10.1016/j.apcatb.2019.04.028
02/11/2019	MoS2@NSCS	0.5 M H2SO4	0.25			158	10	4.00E-02	https://doi.org/10.1016/j.apcatb.2019.118352
06/11/2019	N-MoS2/CN	0.5 M H2SO4	0.29	114	3.62E-01	114	10		https://doi.org/10.1021/jacs.9b09932
19/11/2019	Co1|1T-MoS2	0.5 M H2SO4	0.07	100	7.82E+00	42	10	1.43E-01	https://doi.org/10.1038/s41467-019-12997-7
01/12/2020	1T-2H MoS2	0.5 M H2SO4	0.29			79	1	3.51E-03	https://doi.org/10.1021/acsami.0c16537
01/12/2020	1T-2H MoS2	0.5 M H2SO4	0.29	150	1.51E+02	157	10	3.51E-02	https://doi.org/10.1021/acsami.0c16537
01/12/2020	1T-2H MoS2	0.5 M H2SO4	0.29			211	100	3.51E-01	https://doi.org/10.1021/acsami.0c16537
01/04/2021	V0.05Mo0.95S2	0.5 M H2SO4	0.26			156	10	3.85E-02	https://doi.org/10.1016/j.cej.2020.128158
05/05/2021	MoS2-Ti3C2 MXene	0.5 M H2SO4	0.38	150	1.28E+00	98	10	2.67E-02	https://doi.org/10.1016/j.apcatb.2019.04.028
05/05/2021	MoS2-Ti3C2 MXene	0.5 M H2SO4	0.38	200	2.60E+00				https://doi.org/10.1016/j.apcatb.2019.04.028
12/11/2021	1T-MoS2@Ti	0.5 M H2SO4	0.10			230	10	1.00E-01	https://doi.org/10.1016/j.jechem.2021.10.031
12/11/2021	1T-MoS2/TiO2−x@Ti	0.5 M H2SO4	0.30			146	10	3.33E-02	https://doi.org/10.1016/j.jechem.2021.10.031
22/04/2022	Frenkel-defected monolayer MoS2	0.5 M H2SO4				164	10		https://doi.org/10.1038/s41467-022-29929-7
22/04/2022	pristine MoS2	0.5 M H2SO4				358	10		https://doi.org/10.1038/s41467-022-29929-7

Table S3. Transition metal carbide hydrogen evolution reaction electrocatalyst activities.

Date of publication	Catalyst	Electrolyte	Loading [mg cm^{-2}]	Overpotential for TOF [mV] vs RHE	TOF [H_2 s^{-1} site^{-1}]	Overpotential to achieve geometric current density and mass activity [mV] vs RHE	Geometric current density [mA cm^{-2}]	Mass activity [A mg^{-1}]	DOI
09/11/2012	Mo2C	1 M H2SO4	1.40			210	10	7.14E-03	https://doi.org/10.1002/anie.201207111
31/01/2013	Mo2C/CNT	1 M HClO4	2.00			152	10	5.00E-03	https://doi.org/10.1039/C2EE23891H
17/10/2013	np-Mo2C NW	0.5 M H2SO4	0.21			200	60	2.86E-01	https://doi.org/10.1039/C3EE42441C
23/05/2014	MoxC/Ni-30-725-5	0.5 M H2SO4	38.40	250	8.60E-01	250	35	9.04E-04	https://doi.org/10.1002/cctc.201402000
08/07/2014	Mo2C/GCSs	0.5 M H2SO4	0.36			200	10	2.78E-02	https://doi.org/10.1021/cs5005294
08/07/2014	Mo2C/GCSs	0.5 M H2SO4	0.36			150	2	5.56E-03	https://doi.org/10.1021/cs5005294
30/07/2014	WP/Ti	0.5 M H2SO4	1.00			120	10	1.00E-02	https://doi.org/10.1039/C4CC04709E
30/07/2014	WP/Ti	0.5 M H2SO4	1.00			149	20	2.00E-02	https://doi.org/10.1039/C4CC04709E
11/03/2015	MoCx nano-octahedrons	0.5 M H2SO4	0.80			87	1	1.25E-03	https://doi.org/10.1038/ncomms7512
11/03/2015	MoCx nano-octahedrons	0.5 M H2SO4	0.80			142	10	1.25E-02	https://doi.org/10.1038/ncomms7512
20/03/2015	a-Mo2C	0.5 M H2SO4	0.10	200	5.00E-01	198	10	9.80E-02	https://doi.org/10.1039/C5TA00139K
30/06/2015	Fe3C-GNRs	0.5 M H2SO4	0.14			49	10	7.09E-02	https://doi.org/10.1021/acsnano.5b02420

30/06/2015	Co3C-GNRs	0.5 M H2SO4	0.14			91	10	7.09E-02	https://doi.org/10.1021/acsnano.5b02420
30/06/2015	Ni3C-GNRs	0.5 M H2SO4	0.14			48	10	7.09E-02	https://doi.org/10.1021/acsnano.5b02420
30/06/2015	Fe3C-GNRs	0.5 M H2SO4	0.14			200	167	1.18E+00	https://doi.org/10.1021/acsnano.5b02420
30/06/2015	Co3C-GNRs	0.5 M H2SO4	0.14			200	80	5.65E-01	https://doi.org/10.1021/acsnano.5b02420
30/06/2015	Ni3C-GNRs	0.5 M H2SO4	0.14			200	116	8.26E-01	https://doi.org/10.1021/acsnano.5b02420
16/10/2015	Mo2C NP	0.5 M H2SO4	0.25			78	10	4.00E-02	https://doi.org/10.1002/anie.201506727
22/10/2015	MoSx@Mo2C	0.5 M H2SO4	0.21	400	1.10E+00	400	146	6.85E-01	https://doi.org/10.1021/acscatal.5b01803
22/10/2015	pure Mo2C	0.5 M H2SO4	0.21	400	3.70E-01	400	178	8.36E-01	https://doi.org/10.1021/acscatal.5b01803
05/11/2015	β-Mo2C Nanotubes	0.5 M H2SO4	0.75			172	10	1.33E-02	https://doi.org/10.1002/anie.201508715
05/11/2015	β-Mo2C Nanotubes	0.5 M H2SO4	0.75			197	20	2.67E-02	https://doi.org/10.1002/anie.201508715
01/12/2015	0.27Mo2.4Ni@900	0.5 M H2SO4	1.10			75	10	9.09E-03	https://doi.org/10.1021/jacs.5b07924
01/04/2016	Mo2C@NPC/NPRGO	0.5 M H2SO4	0.14			34	10	7.14E-02	https://doi.org/10.1038/ncomms11204
22/11/2016	Mo2C/NCF	0.5 M H2SO4	0.28			85	1	3.57E-03	https://doi.org/10.1021/acsnano.6b06580
22/11/2016	Mo2C/NCF	0.5 M H2SO4	0.28			144	10	3.57E-02	https://doi.org/10.1021/acsnano.6b06580
19/04/2017	P-Mo2C@C nanowires	0.5 M H2SO4	1.30			89	10	7.69E-03	https://doi.org/10.1039/C7EE00388A
13/10/2017	Mo2C nanobelts	0.5M H2SO4	16.00			140	10	6.25E-04	https://doi.org/10.1016/j.apcatb.2017.10.025

31/10/2017	Ni2PMo2C	0.5 M H2SO4	0.20	200	2.78E+00	154	10	5.00E-02	https://doi.org/10.1021/acs.chemmater.7b03377
31/10/2017	CoPMo2C	0.5 M H2SO4	0.20	200	1.61E+00	158	10	5.00E-02	https://doi.org/10.1021/acs.chemmater.7b03377
31/10/2017	FePMo2C	0.5 M H2SO4	0.20	200	1.30E+00	169	10	5.00E-02	https://doi.org/10.1021/acs.chemmater.7b03377
31/10/2017	Mo2C	0.5 M H2SO4	0.20	200	4.30E-01	206	10	5.00E-02	https://doi.org/10.1021/acs.chemmater.7b03377
22/11/2017	Mo2N-Mo2C	0.5M H2SO4	0.34	100	8.60E-02	157	10	2.97E-02	https://doi.org/10.1002/adma.201704156
03/01/2018	Mo2C-carbon	0.5M H2SO4	0.57			70	10	1.75E-02	https://doi.org/10.1002/advs.201700733
02/03/2018	N-doped WC	0.5M H2SO4	16.00			89	10	6.25E-04	https://doi.org/10.1038/s41467-018-03429-z
02/03/2018	N-WC nanoarray	0.5 M H2SO4	10.00			113	10	1.00E-03	https://doi.org/10.1038/s41467-018-03429-z
02/03/2018	N-WC nanoarray	0.5 M H2SO4	10.00			310	200	2.00E-02	https://doi.org/10.1038/s41467-018-03429-z
05/03/2018	N-Mo2C@C	0.5M H2SO4	0.53			72	10	1.88E-02	https://doi.org/10.1016/j.apcatb.2017.10.025
05/03/2018	Co-Mo2C@C	0.5M H2SO4	0.53			122	10	1.88E-02	https://doi.org/10.1016/j.apcatb.2017.10.025
05/03/2018	Fe-Mo2C@C	0.5M H2SO4	0.53			129	10	1.88E-02	https://doi.org/10.1016/j.apcatb.2017.10.025
05/03/2018	Cr-Mo2C@C	0.5M H2SO4	0.53			147	10	1.88E-02	https://doi.org/10.1016/j.apcatb.2017.10.025
24/05/2018	Ni/WC	0.5M H2SO4	0.70			52	10	1.43E-02	https://doi.org/10.1039/C8EE01129J
10/06/2018	W-C single atom catalyst	0.5M H2SO4	0.41	52	1.00E+00	105	10	2.45E-02	https://doi.org/10.1002/adma.201800396
01/09/2018	MoS2/Ti3C2Tx	0.5M H2SO4	0.28			152	10	3.53E-02	https://doi.org/10.1016/j.apcatb.2018.08.083

20/12/2018	Mo2C-basal functionalized	0.5M H2SO4	1.00	250	3.00E-03	189	10	1.00E-02	https://doi.org/10.1021/acsaem.7b00054
21/12/2018	Co-carbon@Mo2C	0.5M H2SO4	0.83			143	10	1.20E-02	https://doi.org/10.1016/j.nanoen.2018.12.060
17/01/2019	MoS2/Mo2C	0.5M H2SO4	0.30			220	1000	3.33E+00	https://doi.org/10.1038/s41467-018-07792-9
26/01/2019	Ti3C2Ox	0.5M H2SO4	0.40			190	10	2.50E-02	https://doi.org/10.1002/cssc.201803032
06/05/2019	Fe3W3C NRs/RGO	0.5 M H2SO4	0.82	100	2.30E-01	57	10	1.22E-02	https://doi.org/10.1016/j.nanoen.2019.05.009
06/05/2019	WC NRs/RGO	0.5 M H2SO4	0.82	100	2.00E-02	151	10	1.22E-02	https://doi.org/10.1016/j.nanoen.2019.05.009
20/09/2019	Mo2C-Co	1M H2SO4	0.10	250	1.00E-01	250	10	1.00E-01	https://doi.org/10.1021/jacs.9b08897
02/11/2019	Mo2C	0.5M H2SO4	0.25			132	10	4.00E-02	https://doi.org/10.1016/j.apcatb.2019.118352
02/11/2019	Mo2C@NPCS	0.5 M H2SO4	0.25			132	10	4.00E-02	https://doi.org/10.1016/j.apcatb.2019.118352
13/12/2019	MoSe2 –Mo2C	0.5M H2SO4				73	10		https://doi.org/10.1016/j.apcatb.2019.118531
27/12/2019	Mo2C-MoOx/CC	1M HClO4	1.80			60	10	5.56E-03	https://doi.org/10.1002/anie.201914752
25/02/2020	Co-doped Mo2C	1M HClO4	0.39			125	10	2.55E-02	https://doi.org/10.1002/adfm.202000561
02/08/2020	Ni/vanadium carbide	0.5 M H2SO4	2.11	150	2.40E-01	111	10	4.74E-03	https://doi.org/10.1002/aenm.202002260
02/08/2020	Ni/Fe3C	0.5 M H2SO4	2.53			112	10	3.95E-03	https://doi.org/10.1002/aenm.202002260
02/08/2020	Ni/vanadium carbide	0.5 M H2SO4	2.11			270	150	7.11E-02	https://doi.org/10.1002/aenm.202002260
02/08/2020	Ni/Fe3C	0.5 M H2SO4	2.53			291	150	5.93E-02	https://doi.org/10.1002/aenm.202002260

02/08/2020	Ni/VC	0.5M H2SO4	2.11	150	2.40E-01	111	10	4.74E-03	https://doi.org/10.1002/aenm.202002260
02/08/2020	Ni/Fe3C	0.5M H2SO4	2.53			112	10	3.95E-03	https://doi.org/10.1002/aenm.202002260
24/11/2020	Co-Mo2C/N-doped carbon	0.5M H2SO4	1.13			116	10	8.83E-03	https://doi.org/10.1016/j.apcatb.2020.119738
29/03/2021	Mo2C/CFP	0.5M H2SO4	2.00			56	10	5.00E-03	https://doi.org/10.1039/D1NR00169H
07/04/2021	WC650	0.5 M H2SO4	0.53			120	10	1.89E-02	https://doi.org/10.1088/2632-959X/abf2ad
13/11/2021	β-Mo2C	0.5 M H2SO4	0.41	200	3.09E+00	156	100	2.46E-01	https://doi.org/10.1021/acsanm.1c02770
22/11/2021	MoC-Mo2C-790 oC	0.5M H2SO4		250	1.30E+00	114	10		https://doi.org/10.1038/s41467-021-27118-6
18/07/2022	Ni-doped Mo2C@CFP	0.5M H2SO4	2.00			56	10	5.00E-03	https://doi.org/10.1007/s40145-022-0610-6

Table S4. Platinum-based hydrogen evolution reaction electrocatalyst activities.

**Date of publication**	**Catalyst**	**Electrolyte**	**Loading [mg cm^{-2}]**	**Overpotential for TOF [mV] vs RHE**	**TOF [H2 s^{-1} $site^{-1}$]**	**Overpotential to achieve geometric current density and mass activity [mV] vs RHE**	**Geometric current density [mA cm^{-2}]**	**Mass activity [A mg^{-1}]**	**TOF [H_2 s^{-1} $site^{-1}$] at j = 10mA cm^{-2}**	**DOI**
05/07/1905	Pt on Floating electrode	4 M HClO4	8.40E-04	1.00E-01	3.01E+03	0.10	1260	1.29E+03		https://doi.org/10.1149/05801.0039ecst
05/07/1905	Pt on Floating electrode	4 M HClO4	8.40E-04	2.00E-01	7.43E+03	0.20	3040	3.18E+03		https://doi.org/10.1149/05801.0039ecst

27/08/2015	Pt-TiO2–Ti-pn+Si	1 M HClO4	1.00E-03			32.00	10	1.00E+01		https://doi.org/10.1039/C5EE02188J
17/03/2017	400-SWNT/Pt	0.5 M H2SO4	5.70E-04			27.00	10	1.75E+01		https://doi.org/10.1021/acscatal.7b00199
30/07/2018	Pt-GT-1	0.5 M H2SO4	1.40E-03			18.00	10	7.14E+00	7.20E+00	https://doi.org/10.1038/s41560-018-0209-x
22/11/2018	PtNP/OMC	0.5 M H2SO4	1.10E-03			33.00	10	9.09E+00		https://doi.org/10.1016/j.jcat.2018.11.006
12/12/2018	$Mo_2TiC_2T_x$–Pt_{SA}	0.5 M H2SO4	1.20E-02			30.00	10	8.33E-01		https://doi.org/10.1038/s41929-018-0195-1
19/03/2021	UHV Pt NP - Small raster	0.5 M H2SO4	1.30E-05	1.50E-02	3.10E+02	133.00	10	7.69E+02	6.60E+03	https://doi.org/10.1021/acsenergylett.1c00246
19/03/2021	UHV Pt NP - Small raster	0.5 M H2SO4	1.90E-05	1.50E-02	2.60E+02	115.00	10	5.26E+02	4.20E+03	https://doi.org/10.1021/acsenergylett.1c00246
19/03/2021	UHV Pt NP - Small raster	0.5 M H2SO4	5.00E-05	1.50E-02	1.10E+02	85.00	10	2.00E+02	1.40E+03	https://doi.org/10.1021/acsenergylett.1c00246
19/03/2021	UHV Pt NP - Small raster	0.5 M H2SO4	1.00E-04	1.50E-02	7.90E+01	61.00	10	1.00E+02	7.50E+02	https://doi.org/10.1021/acsenergylett.1c00246
19/03/2021	UHV Pt NP - Small raster	0.5 M H2SO4	1.70E-05	1.50E-02	2.40E+02	120.00	10	5.88E+02	4.60E+03	https://doi.org/10.1021/acsenergylett.1c00246
19/03/2021	UHV Pt NP - Small raster	0.5 M H2SO4	5.00E-05	1.50E-02	9.80E+01	83.00	10	2.00E+02	1.20E+03	https://doi.org/10.1021/acsenergylett.1c00246

19/03/2021	UHV Pt NP - Small raster	0.5 M H2SO4	1.00E-04	1.50E-02	7.30E+01	54.00	10	1.00E+02	6.30E+02	https://doi.org/10.1021/acsenergylett.1c00246
19/03/2021	UHV Pt NP - Small raster	0.5 M H2SO4	2.00E-04	1.50E-02	4.80E+01	41.00	10	5.00E+01	3.00E+02	https://doi.org/10.1021/acsenergylett.1c00246
19/03/2021	UHV Pt NP - Small raster	0.5 M H2SO4	2.00E-04	1.50E-02	5.80E+01	48.00	10	5.00E+01	3.90E+02	https://doi.org/10.1021/acsenergylett.1c00246
19/03/2021	UHV Pt NP - Small raster	0.5 M H2SO4	5.00E-04	1.50E-02	2.80E+01	35.00	10	2.00E+01	1.20E+02	https://doi.org/10.1021/acsenergylett.1c00246
19/03/2021	UHV Pt NP - Small raster	0.5 M H2SO4	5.00E-04	1.50E-02	3.60E+01	34.00	10	2.00E+01	1.30E+02	https://doi.org/10.1021/acsenergylett.1c00246
19/03/2021	UHV Pt NP - Small raster	0.5 M H2SO4	1.00E-03	1.50E-02	2.20E+01	29.00	10	1.00E+01	7.10E+01	https://doi.org/10.1021/acsenergylett.1c00246
19/03/2021	UHV Pt NP - Small raster	0.5 M H2SO4	5.00E-03	1.50E-02	9.00E+00	20.00	10	2.00E+00	1.80E+01	https://doi.org/10.1021/acsenergylett.1c00246
19/03/2021	UHV Pt NP - Large raster	0.5 M H2SO4	5.65E-04	1.50E-02	4.70E+01	24.00	10	1.77E+01	1.00E+02	https://doi.org/10.1021/acsenergylett.1c00246
19/03/2021	UHV Pt NP - Large raster	0.5 M H2SO4	1.00E-03	1.50E-02	2.40E+01	21.00	10	1.00E+01	4.40E+01	https://doi.org/10.1021/acsenergylett.1c00246
19/03/2021	UHV Pt NP - Large raster	0.5 M H2SO4	5.00E-03	1.50E-02	1.30E+01	16.00	10	2.00E+00	1.30E+01	https://doi.org/10.1021/acsenergylett.1c00246
19/03/2021	Commercial Pt/C	0.5 M H2SO4	5.00E-04	1.50E-02	4.00E+01	30.00	10	2.00E+01	1.30E+02	https://doi.org/10.1021/acsenergylett.1c00246

19/03/2021	Commercial Pt/C	0.5 M H2SO4	1.00E-03	1.50E-02	1.70E+01	30.00	10	1.00E+01	5.50E+01	https://doi.org/10.1021/acsenergylett.1c00246
19/03/2021	Commercial Pt/C	0.5 M H2SO4	5.00E-03	1.50E-02	5.00E+00	21.00	10	2.00E+00	8.80E+00	https://doi.org/10.1021/acsenergylett.1c00246
19/03/2021	Commercial Pt/C	0.5 M H2SO4	5.00E-02	1.50E-02	3.00E-01	21.00	10	2.00E-01	6.90E-01	https://doi.org/10.1021/acsenergylett.1c00246
19/03/2021	Commercial Pt/C	0.5 M H2SO4	1.00E-01	1.50E-02	4.00E-01	18.00	10	1.00E-01	6.00E-01	https://doi.org/10.1021/acsenergylett.1c00246
